# Supplementary figures and images for: The FAM104 proteins VCF1/2 promote the nuclear localization of p97/VCP (part 1 of 2)
Source: eLife. 2023 Sep 15;12:e92409. doi: 10.7554/eLife.92409 (PMC10541173; doi:10.7554/eLife.92409)

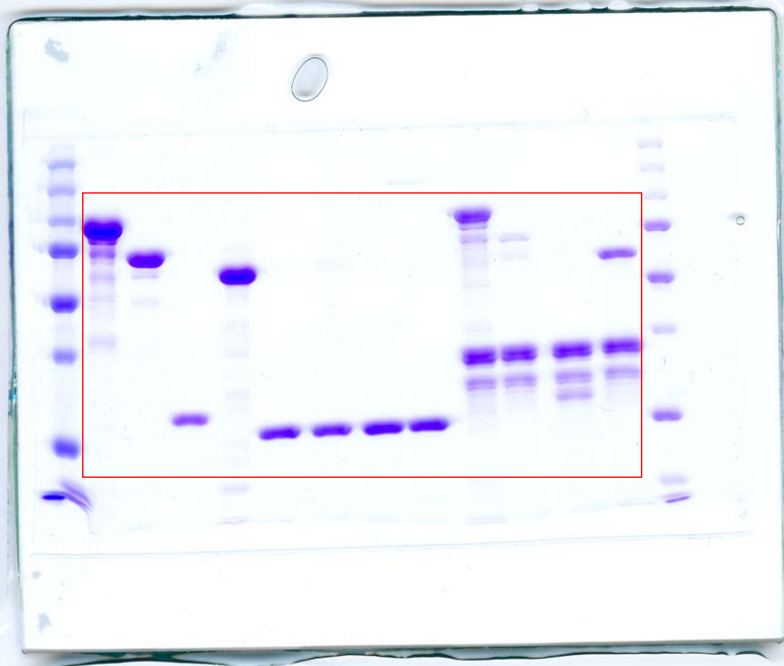

Supplement: Figure 1—source data 1. [file elife-92409-fig1-data1.zip › Figure 1-source data 1/Uncropped Labelled/Panel F - Coomassie.pdf]

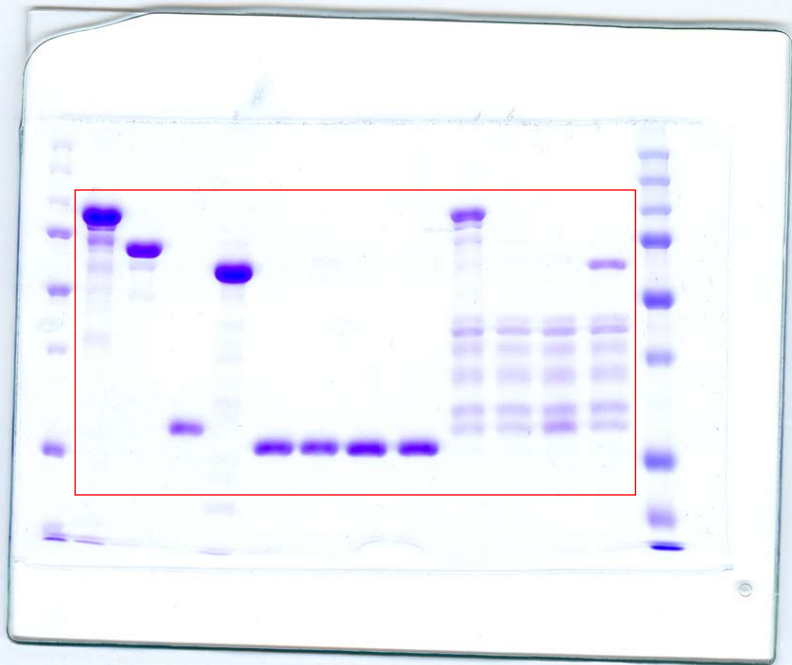

Supplement: Figure 1—source data 1. [file elife-92409-fig1-data1.zip › Figure 1-source data 1/Uncropped Labelled/Panel E - Coomassie.pdf]

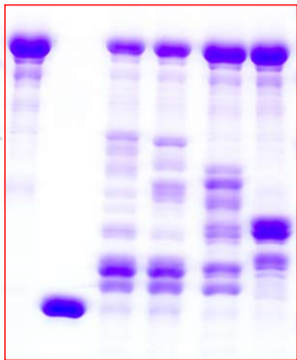

Supplement: Figure 1—source data 1. [file elife-92409-fig1-data1.zip › Figure 1-source data 1/Uncropped Labelled/Panel D - Coomassie.pdf]

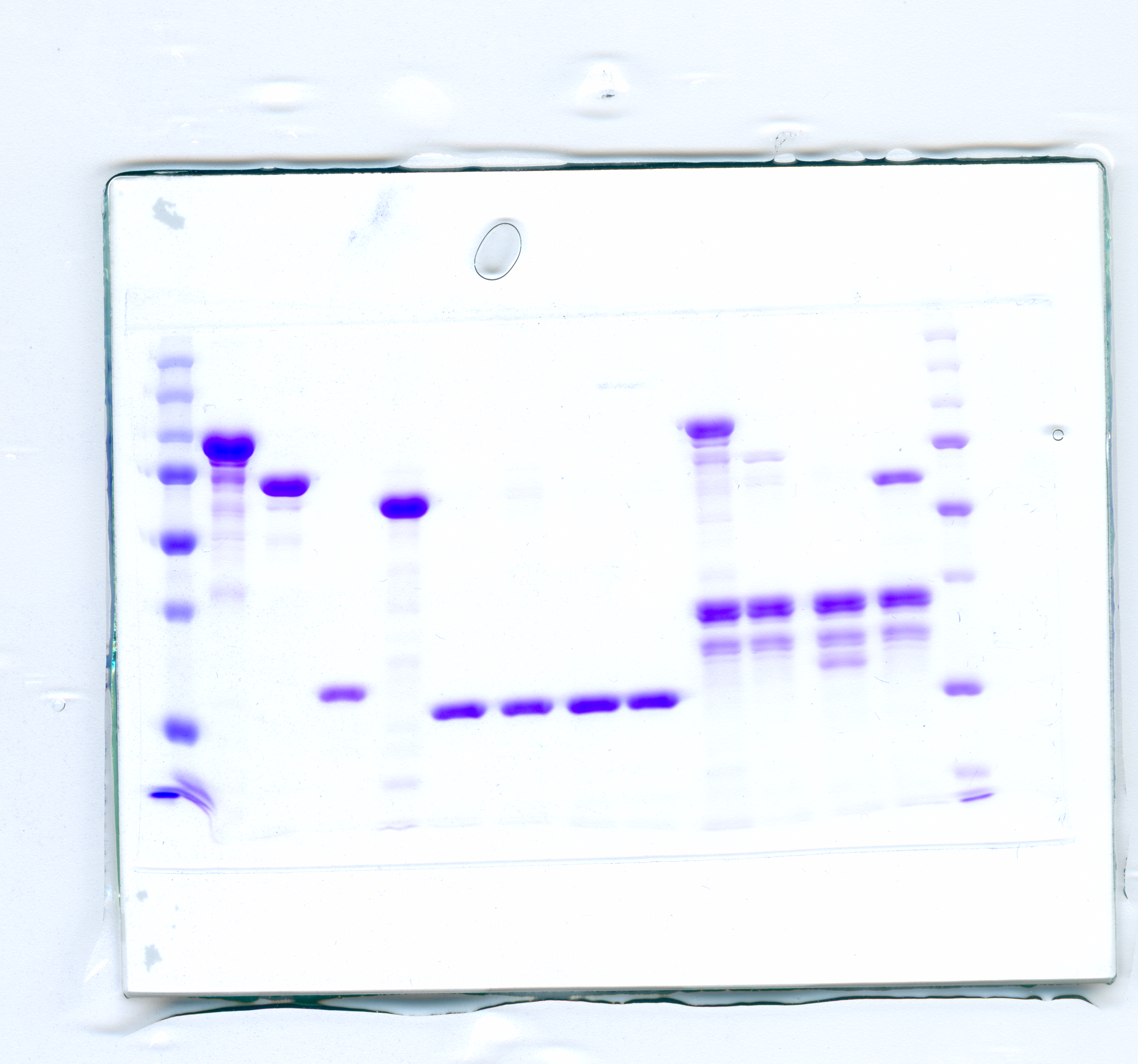

Supplement: Figure 1—source data 1. [file elife-92409-fig1-data1.zip › Figure 1-source data 1/Uncropped Originals/Panel F - Coomassie.tif]

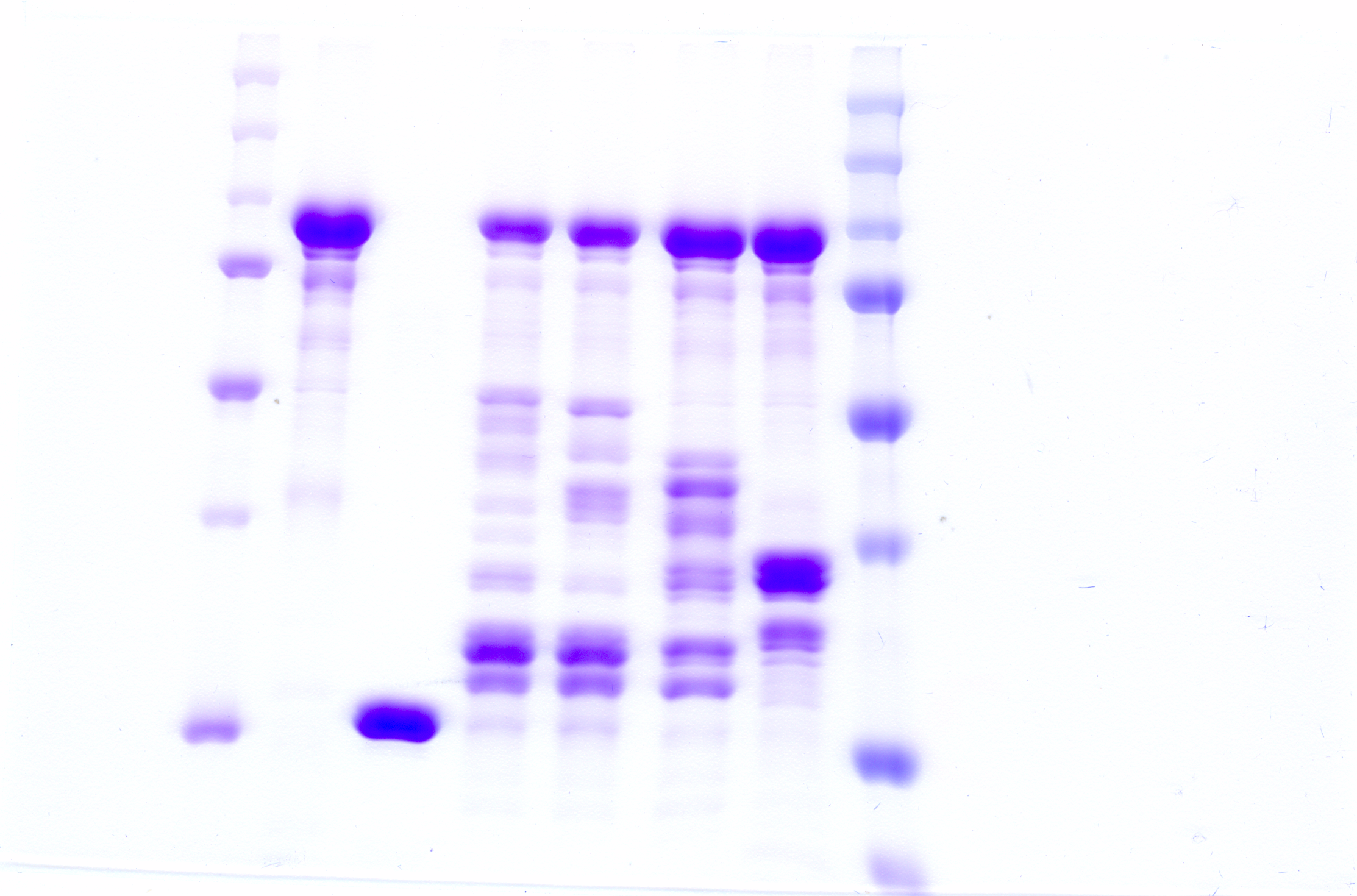

Supplement: Figure 1—source data 1. [file elife-92409-fig1-data1.zip › Figure 1-source data 1/Uncropped Originals/Panel D - Coomassie.tif]

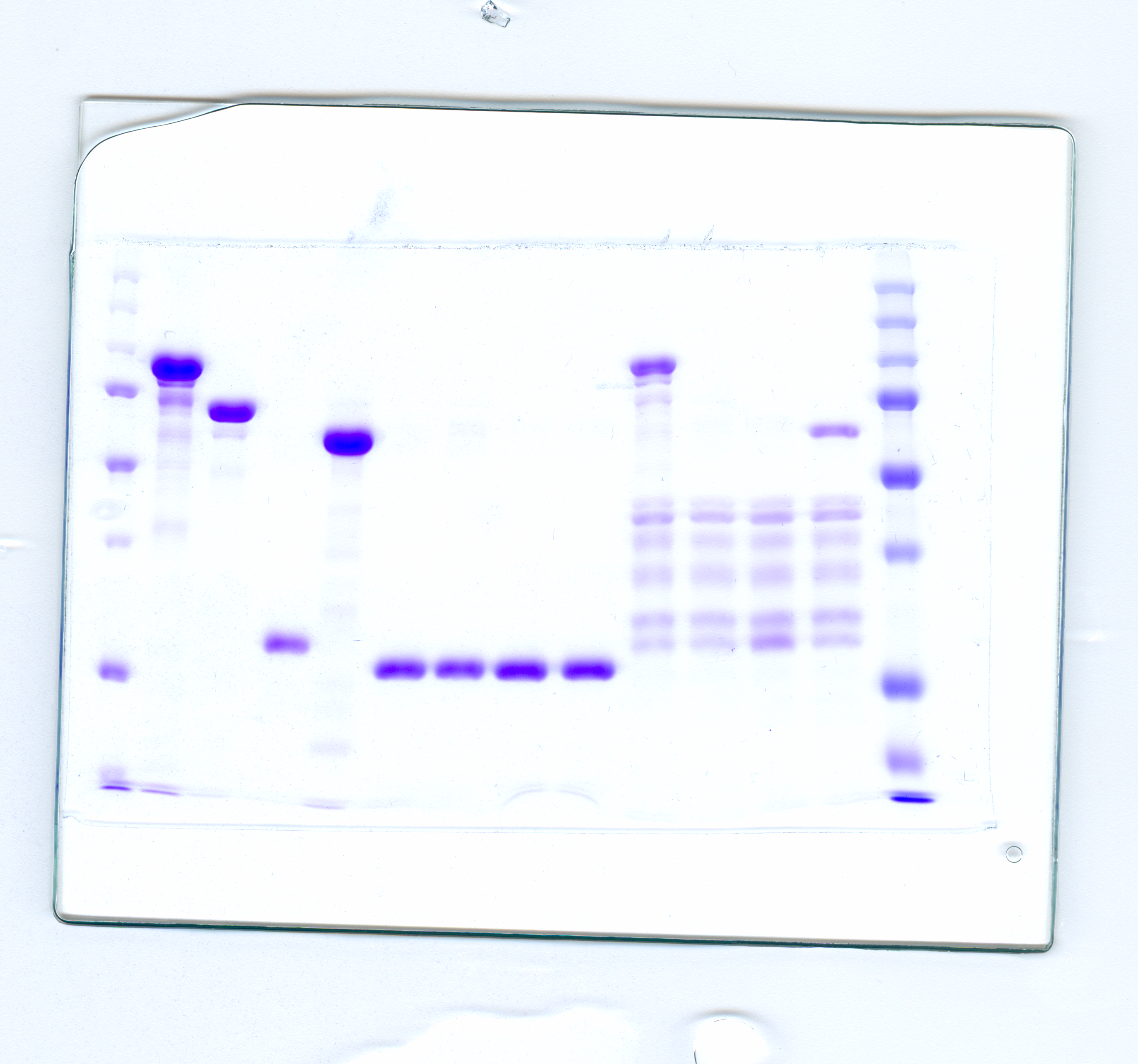

Supplement: Figure 1—source data 1. [file elife-92409-fig1-data1.zip › Figure 1-source data 1/Uncropped Originals/Panel E - Coomassie.tif]

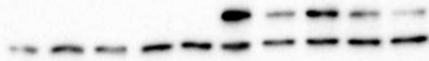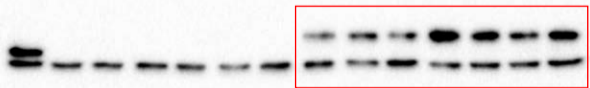

Supplement: Figure 2—source data 1. [file elife-92409-fig2-data1.zip › Figure 2-source data 1/Uncropped Labelled/Panel C - p97 blot.pdf]

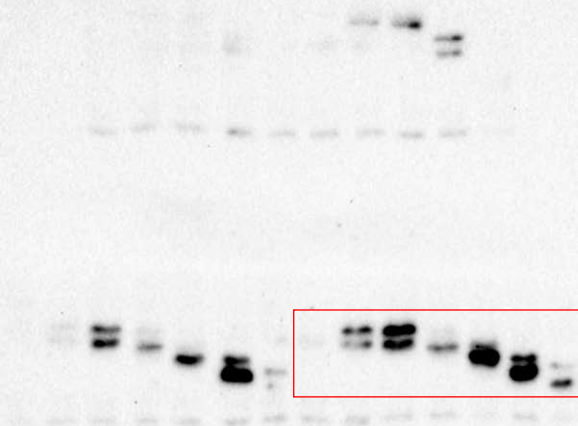

Supplement: Figure 2—source data 1. [file elife-92409-fig2-data1.zip › Figure 2-source data 1/Uncropped Labelled/Panel C - Gal4-TA blot.pdf]

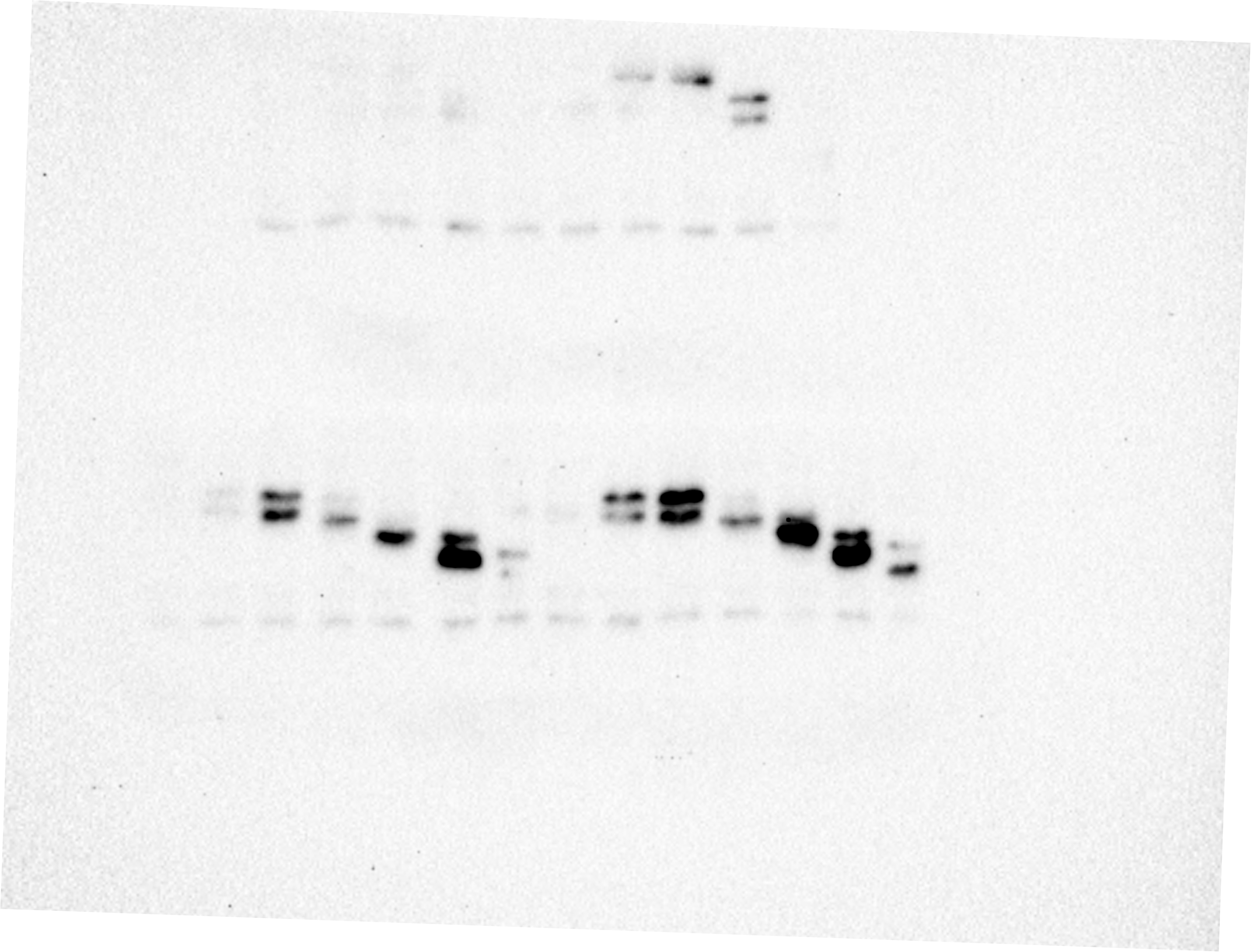

Supplement: Figure 2—source data 1. [file elife-92409-fig2-data1.zip › Figure 2-source data 1/Uncropped Originals/Panel C - Gal4-TA blot.tif]

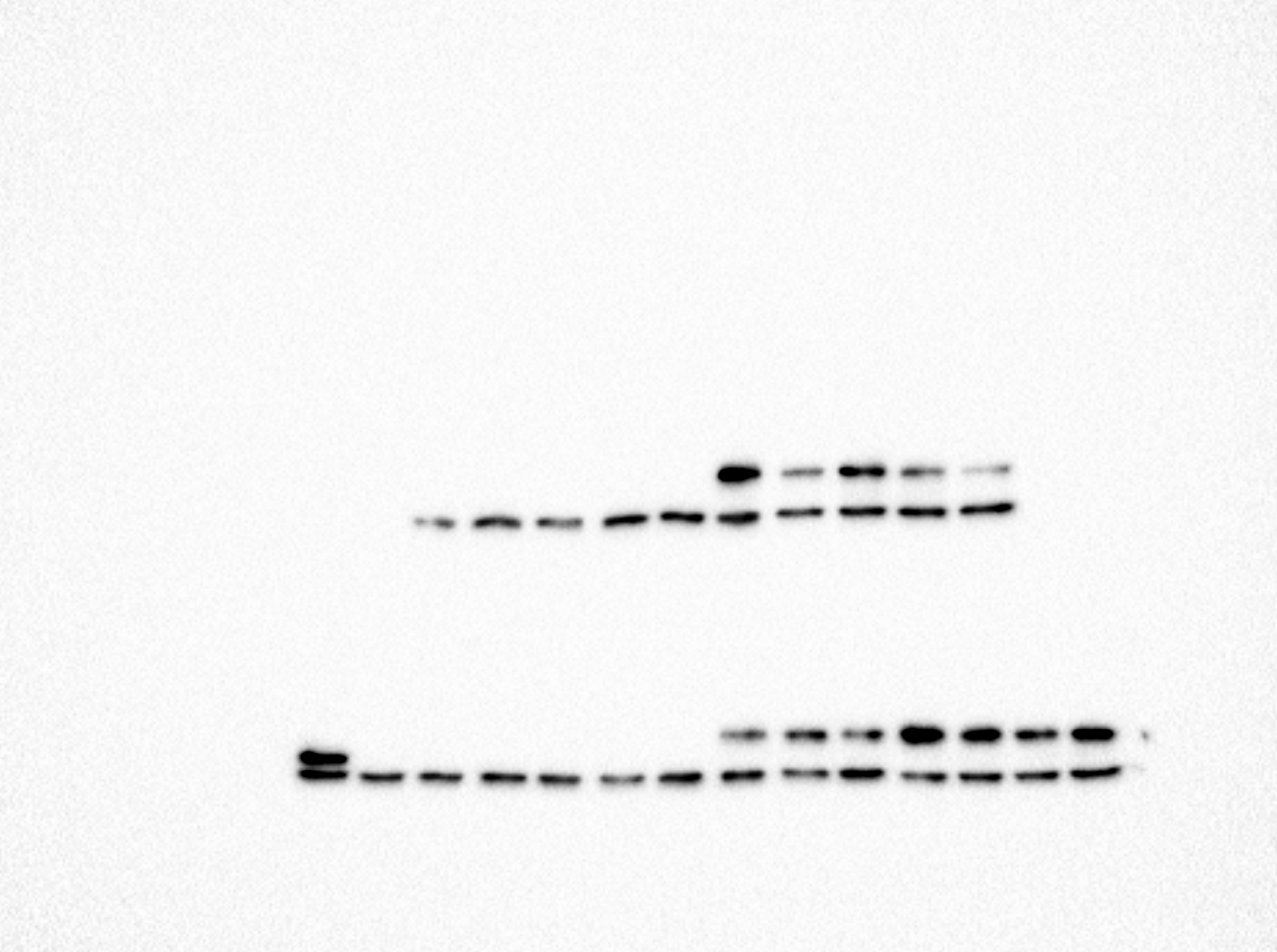

Supplement: Figure 2—source data 1. [file elife-92409-fig2-data1.zip › Figure 2-source data 1/Uncropped Originals/Panel C - p97 blot.tif]

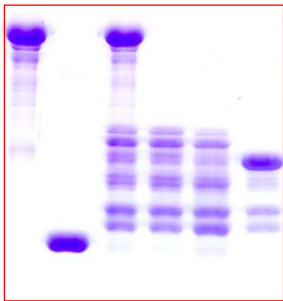

Supplement: Figure 2—source data 2. [file elife-92409-fig2-data2.zip › Figure 2-source data 2/Uncropped Labelled/Panel H - Coomassie.pdf]

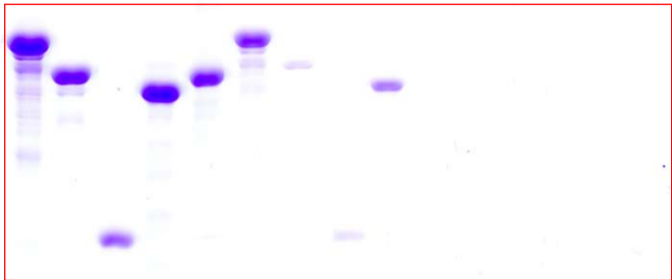

Supplement: Figure 2—source data 2. [file elife-92409-fig2-data2.zip › Figure 2-source data 2/Uncropped Labelled/Panel E - Coomassie.pdf]

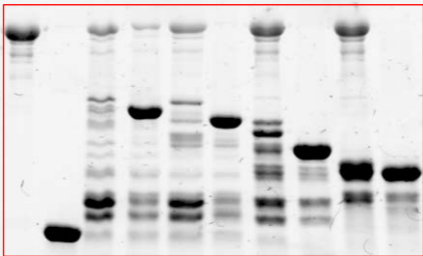

Supplement: Figure 2—source data 2. [file elife-92409-fig2-data2.zip › Figure 2-source data 2/Uncropped Labelled/Panel D - TCE.pdf]

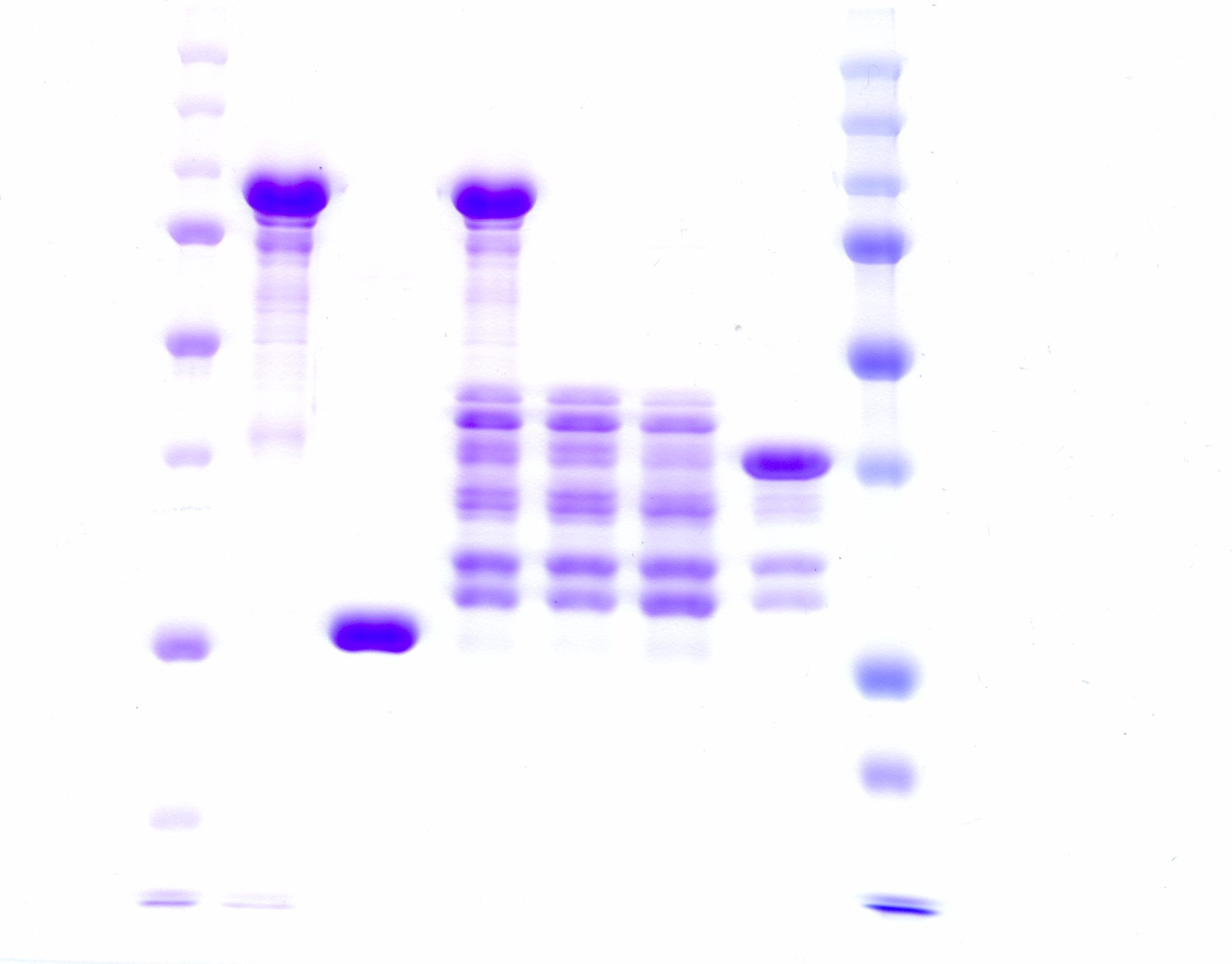

Supplement: Figure 2—source data 2. [file elife-92409-fig2-data2.zip › Figure 2-source data 2/Uncropped Originals/Panel H - Coomassie.tif]

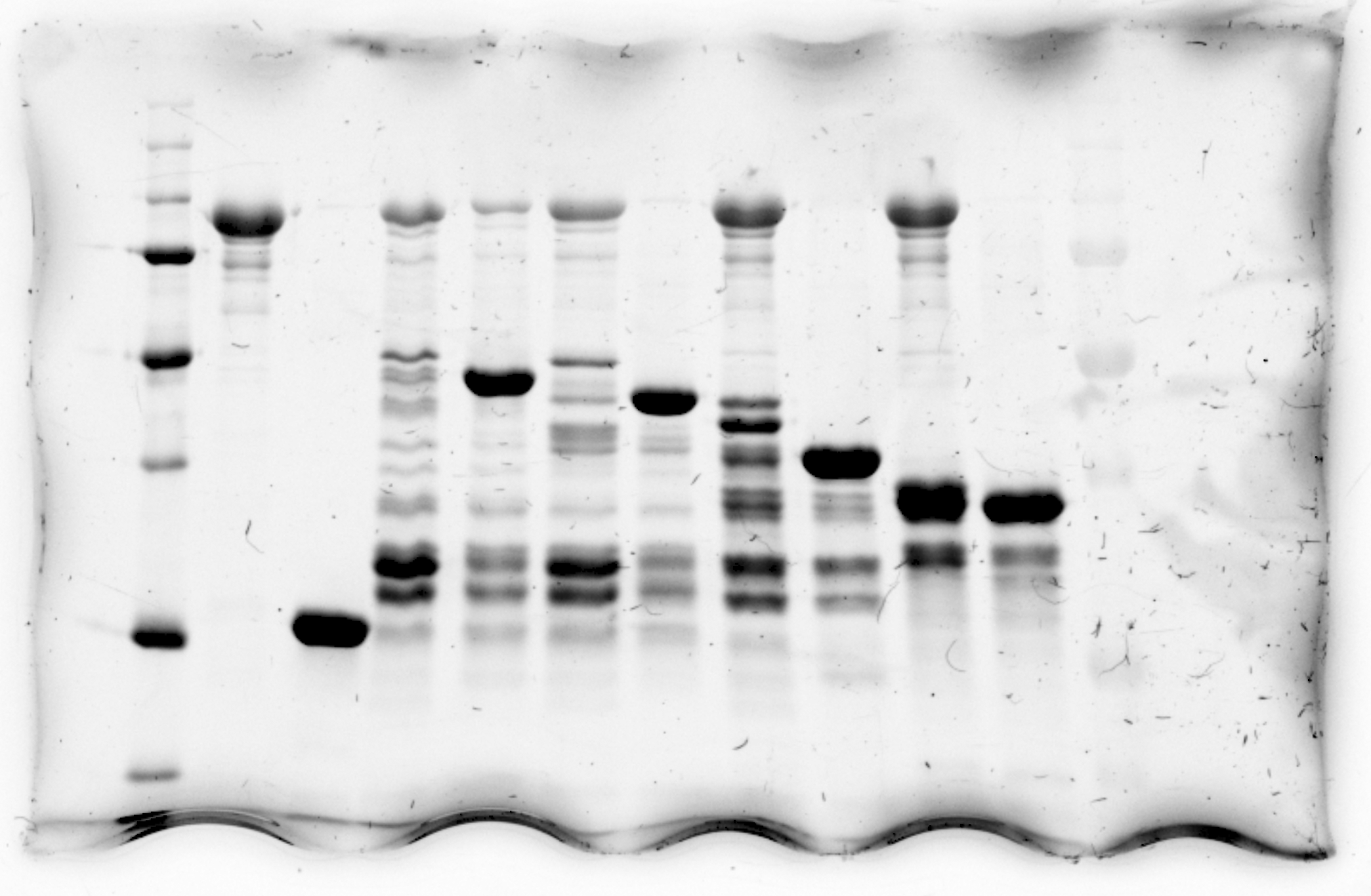

Supplement: Figure 2—source data 2. [file elife-92409-fig2-data2.zip › Figure 2-source data 2/Uncropped Originals/Panel D - TCE.tif]

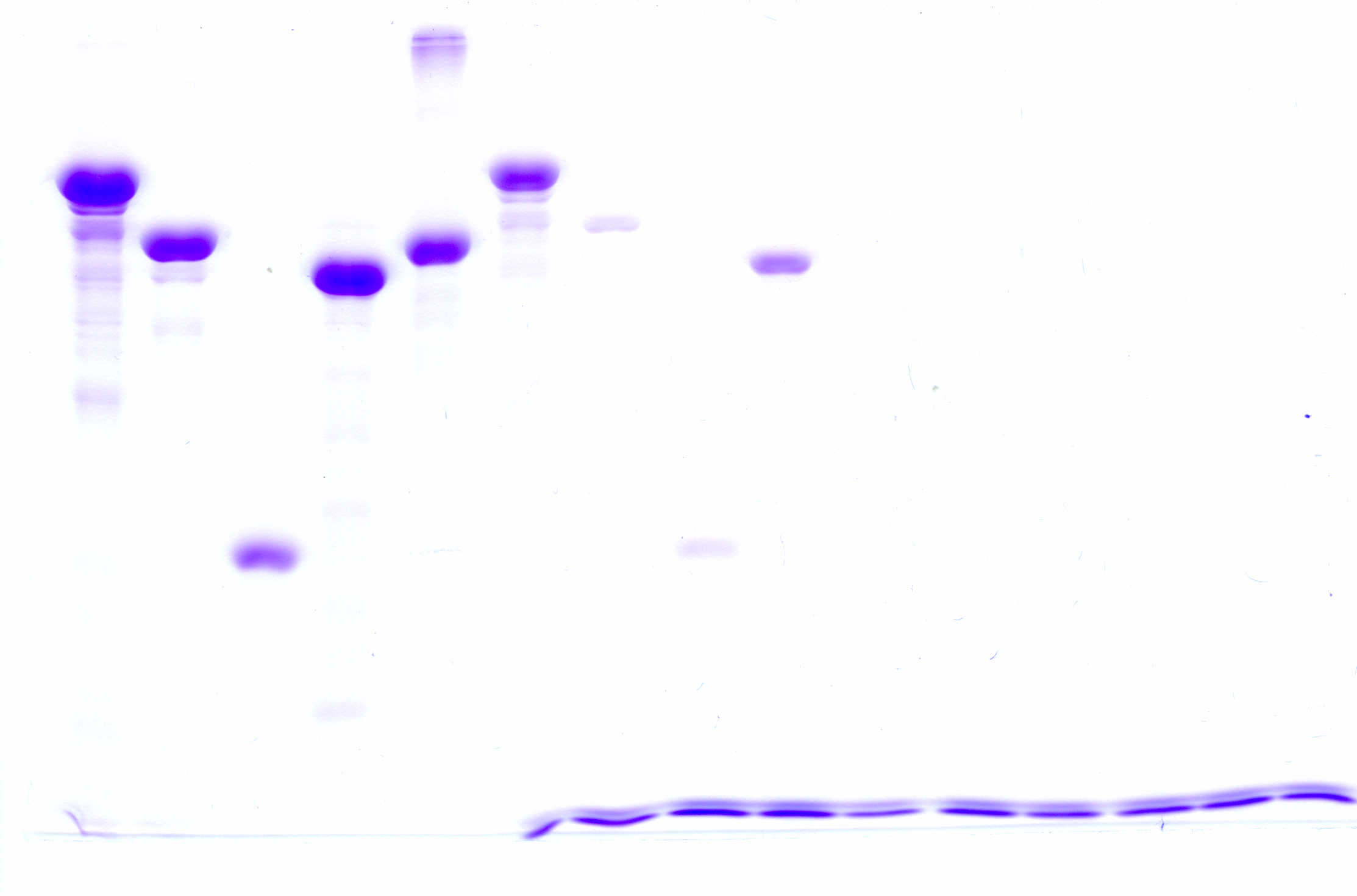

Supplement: Figure 2—source data 2. [file elife-92409-fig2-data2.zip › Figure 2-source data 2/Uncropped Originals/Panel E - Coomassie.tif]

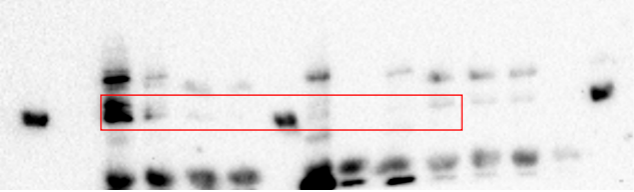

Supplement: Figure 3—source data 1. [file elife-92409-fig3-data1.zip › Figure 3-source data 1/Uncropped Labelled/PanelA - FAF1 IP blot.pdf]

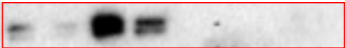

Supplement: Figure 3—source data 1. [file elife-92409-fig3-data1.zip › Figure 3-source data 1/Uncropped Labelled/PanelA - UBXN2B IP blot.pdf]

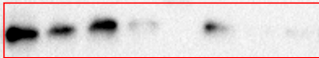

Supplement: Figure 3—source data 1. [file elife-92409-fig3-data1.zip › Figure 3-source data 1/Uncropped Labelled/PanelA - UBXN7 IP blot.pdf]

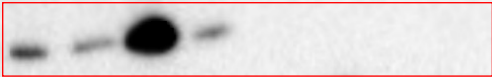

Supplement: Figure 3—source data 1. [file elife-92409-fig3-data1.zip › Figure 3-source data 1/Uncropped Labelled/PanelA - UFD1 IP blot.pdf]

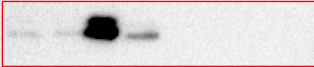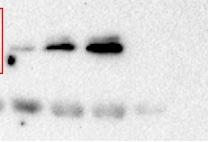

Supplement: Figure 3—source data 1. [file elife-92409-fig3-data1.zip › Figure 3-source data 1/Uncropped Labelled/PanelA - NPL4 IP blot.pdf]

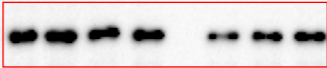

Supplement: Figure 3—source data 1. [file elife-92409-fig3-data1.zip › Figure 3-source data 1/Uncropped Labelled/PanelA - UFD1 Input blot.pdf]

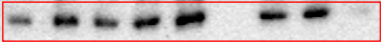

Supplement: Figure 3—source data 1. [file elife-92409-fig3-data1.zip › Figure 3-source data 1/Uncropped Labelled/PanelA - UBXN2B Input blot.pdf]

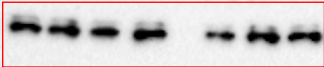

Supplement: Figure 3—source data 1. [file elife-92409-fig3-data1.zip › Figure 3-source data 1/Uncropped Labelled/PanelA - NPL4 Input blot.pdf]

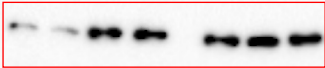

Supplement: Figure 3—source data 1. [file elife-92409-fig3-data1.zip › Figure 3-source data 1/Uncropped Labelled/PanelA - p97 Input blot.pdf]

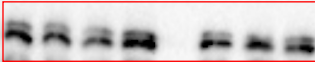

Supplement: Figure 3—source data 1. [file elife-92409-fig3-data1.zip › Figure 3-source data 1/Uncropped Labelled/PanelA - FAF1 Input blot.pdf]

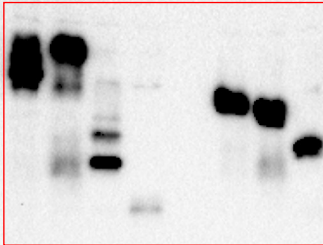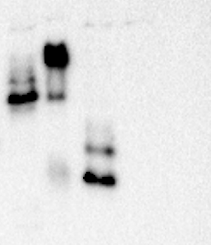

Supplement: Figure 3—source data 1. [file elife-92409-fig3-data1.zip › Figure 3-source data 1/Uncropped Labelled/PanelA - Flag Input blot.pdf]

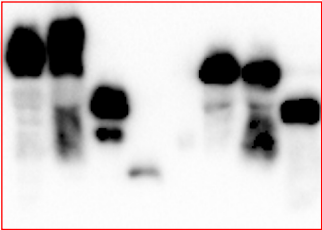

Supplement: Figure 3—source data 1. [file elife-92409-fig3-data1.zip › Figure 3-source data 1/Uncropped Labelled/PanelA - Flag IP blot.pdf]

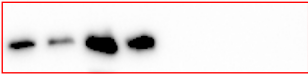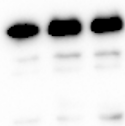

Supplement: Figure 3—source data 1. [file elife-92409-fig3-data1.zip › Figure 3-source data 1/Uncropped Labelled/PanelA - p97 IP blot.pdf]

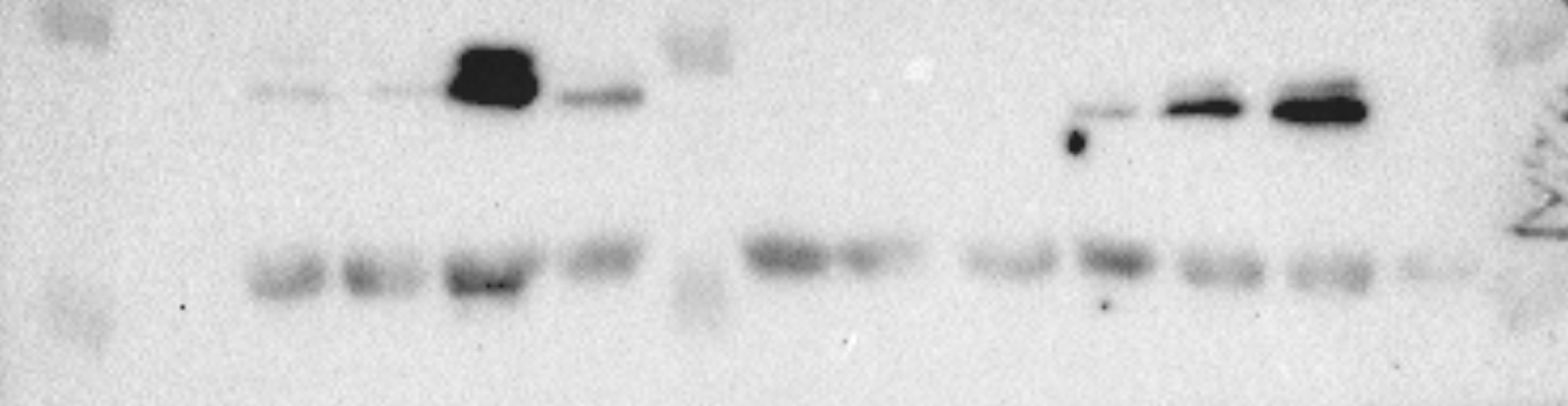

Supplement: Figure 3—source data 1. [file elife-92409-fig3-data1.zip › Figure 3-source data 1/Uncropped Originals /Originals merged with marker /PanelA - NPL4 IP blot.tif]

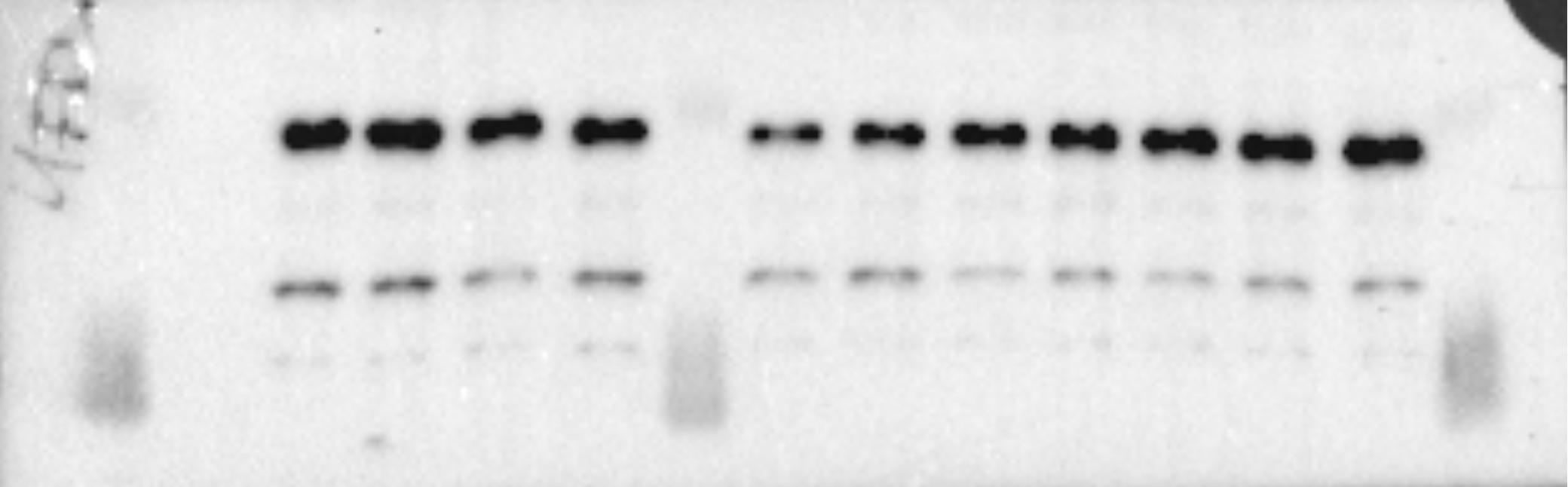

Supplement: Figure 3—source data 1. [file elife-92409-fig3-data1.zip › Figure 3-source data 1/Uncropped Originals /Originals merged with marker /PanelA - UFD1 input blot.tif]

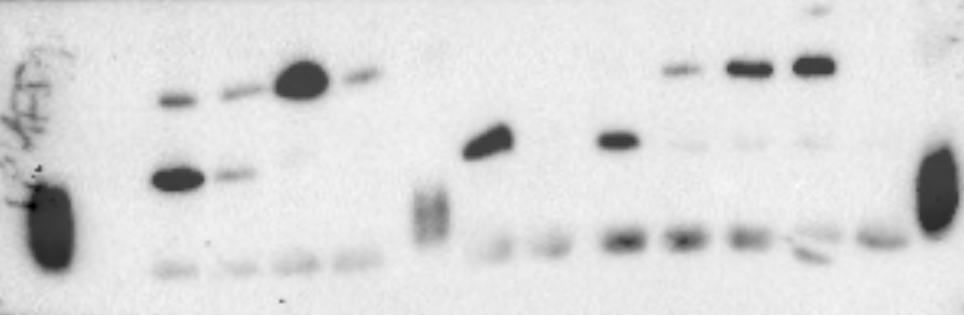

Supplement: Figure 3—source data 1. [file elife-92409-fig3-data1.zip › Figure 3-source data 1/Uncropped Originals /Originals merged with marker /PanelA - UFD1 IP blot.tif]

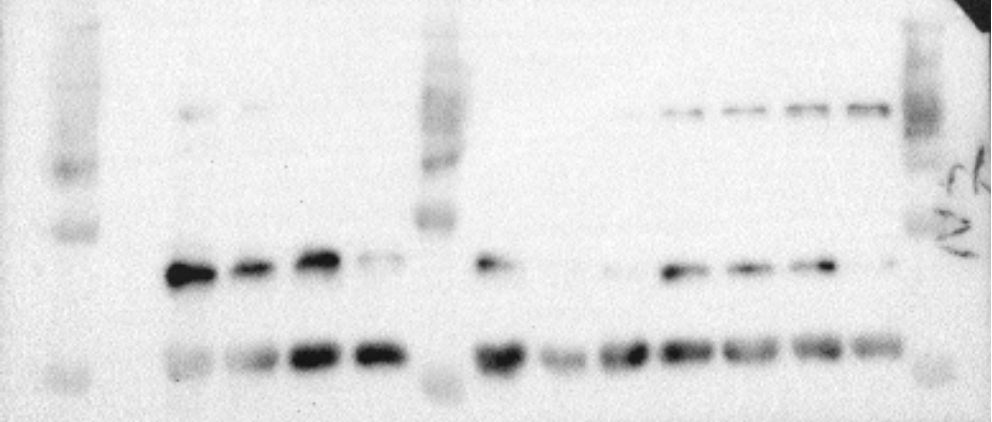

Supplement: Figure 3—source data 1. [file elife-92409-fig3-data1.zip › Figure 3-source data 1/Uncropped Originals /Originals merged with marker /PanelA - UBXN7 IP blot.tif]

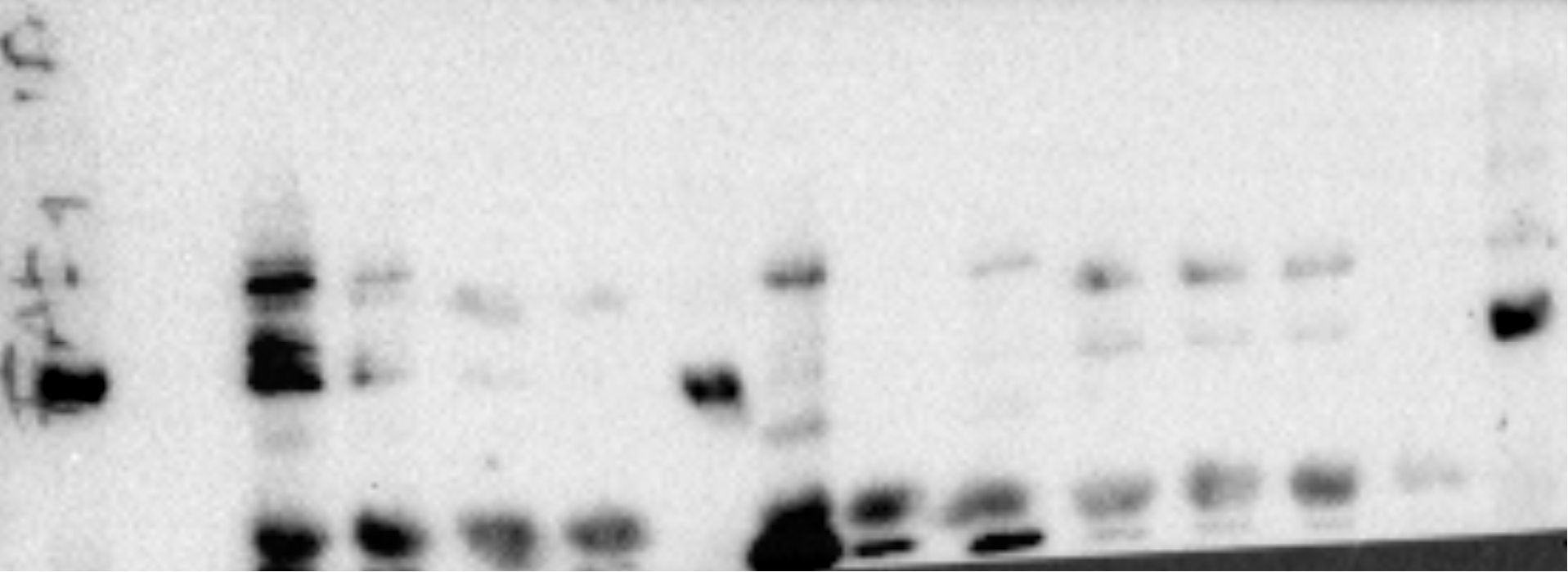

Supplement: Figure 3—source data 1. [file elife-92409-fig3-data1.zip › Figure 3-source data 1/Uncropped Originals /Originals merged with marker /PanelA - FAF1 IP blot.tif]

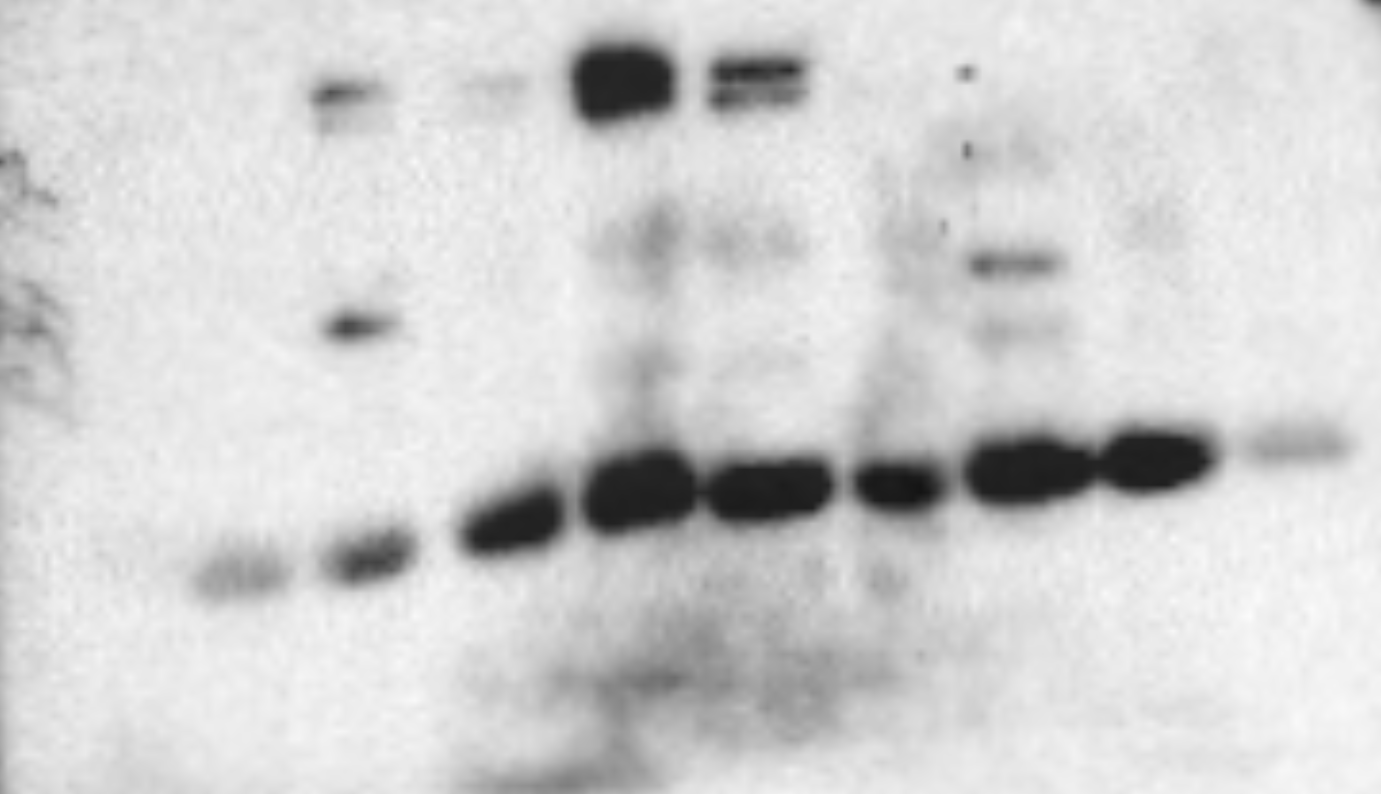

Supplement: Figure 3—source data 1. [file elife-92409-fig3-data1.zip › Figure 3-source data 1/Uncropped Originals /Originals merged with marker /PanelA - UBXN2B IP blot.tif]

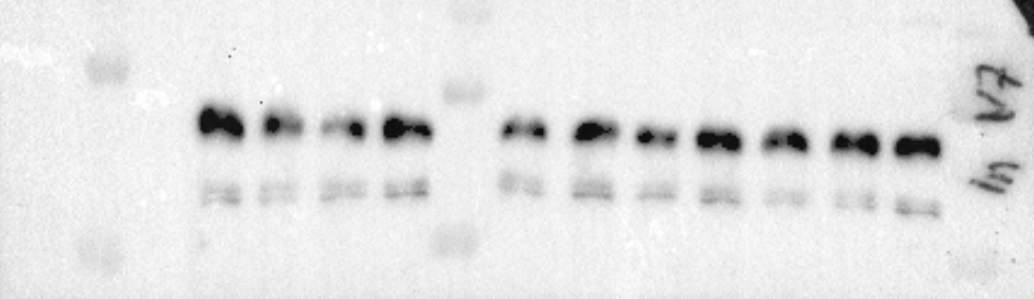

Supplement: Figure 3—source data 1. [file elife-92409-fig3-data1.zip › Figure 3-source data 1/Uncropped Originals /Originals merged with marker /PanelA - UBXN7 input blot.tif]

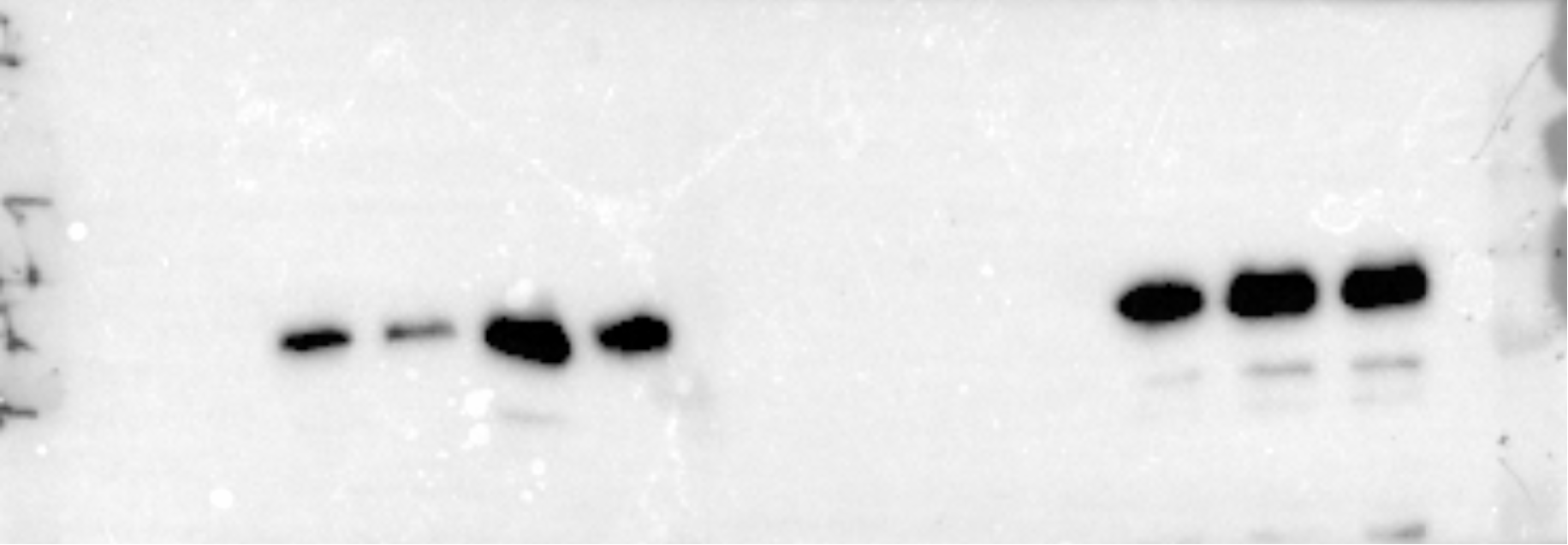

Supplement: Figure 3—source data 1. [file elife-92409-fig3-data1.zip › Figure 3-source data 1/Uncropped Originals /Originals merged with marker /PanelA - p97 IP blot.tif]

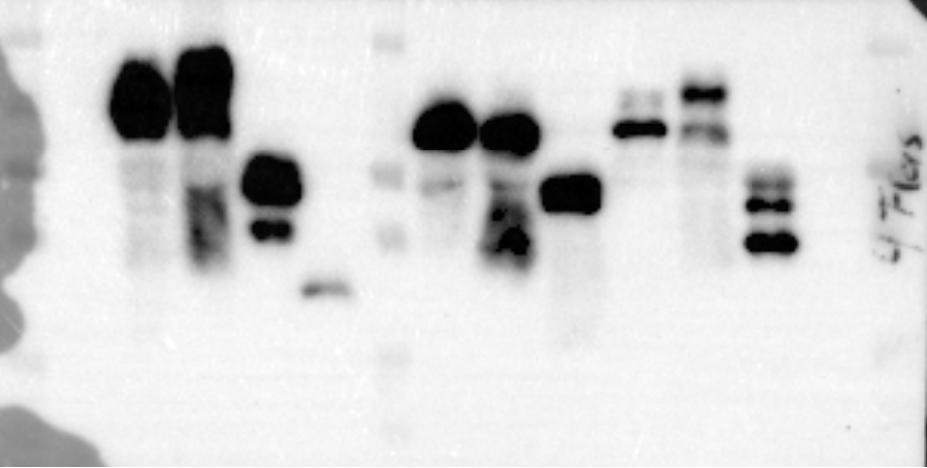

Supplement: Figure 3—source data 1. [file elife-92409-fig3-data1.zip › Figure 3-source data 1/Uncropped Originals /Originals merged with marker /PanelA - Flag IP blot.tif]

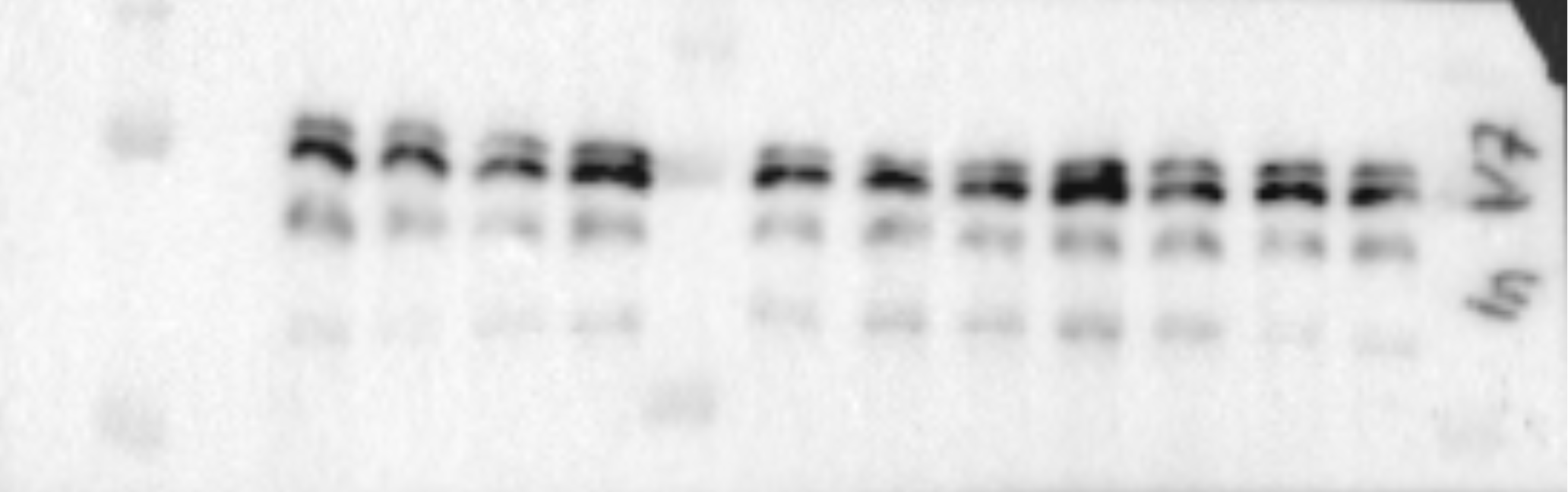

Supplement: Figure 3—source data 1. [file elife-92409-fig3-data1.zip › Figure 3-source data 1/Uncropped Originals /Originals merged with marker /PanelA - FAF1 input blot.tif]

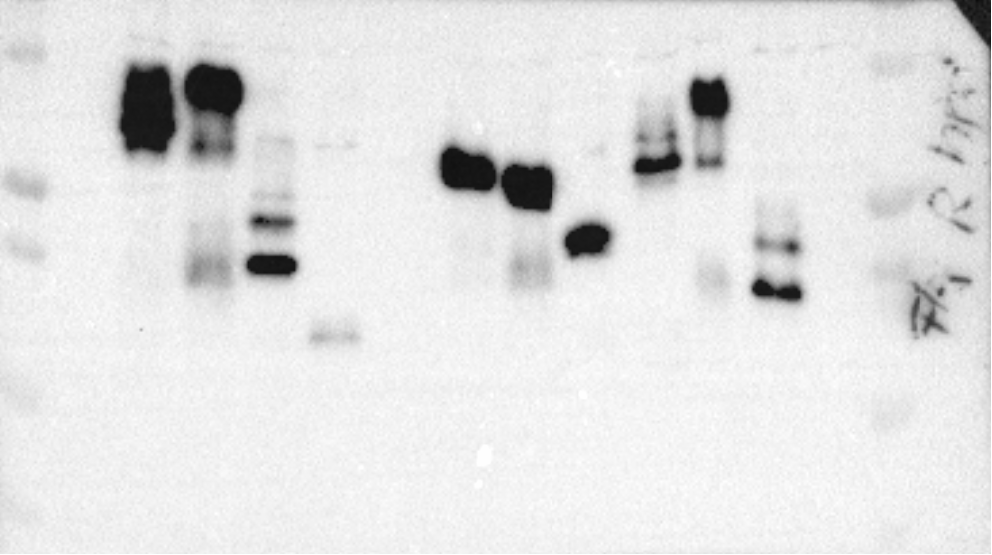

Supplement: Figure 3—source data 1. [file elife-92409-fig3-data1.zip › Figure 3-source data 1/Uncropped Originals /Originals merged with marker /PanelA - Flag input blot.tif]

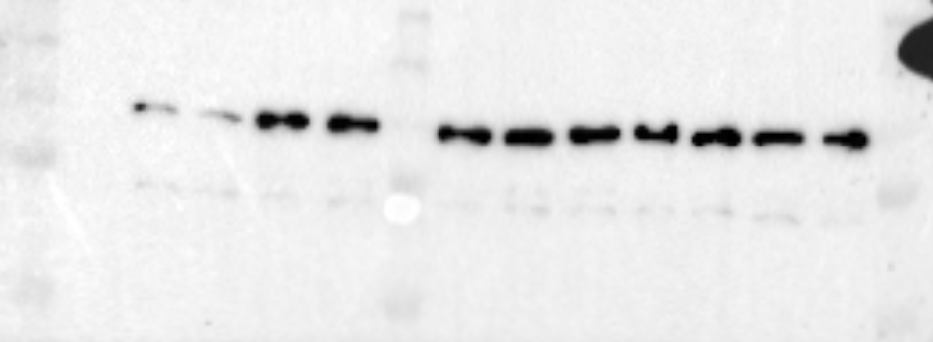

Supplement: Figure 3—source data 1. [file elife-92409-fig3-data1.zip › Figure 3-source data 1/Uncropped Originals /Originals merged with marker /PanelA - p97 input blot.tif]

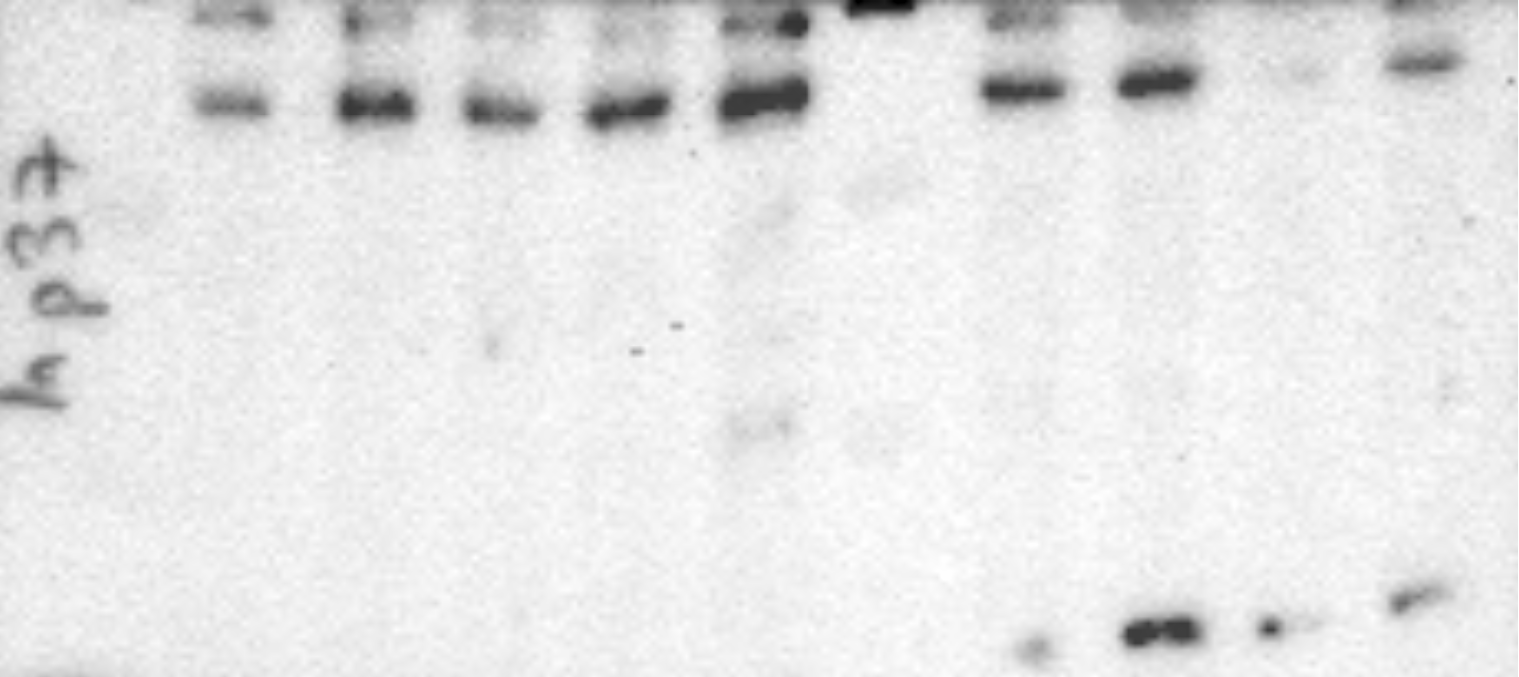

Supplement: Figure 3—source data 1. [file elife-92409-fig3-data1.zip › Figure 3-source data 1/Uncropped Originals /Originals merged with marker /PanelA - UBXN2B input blot.tif]

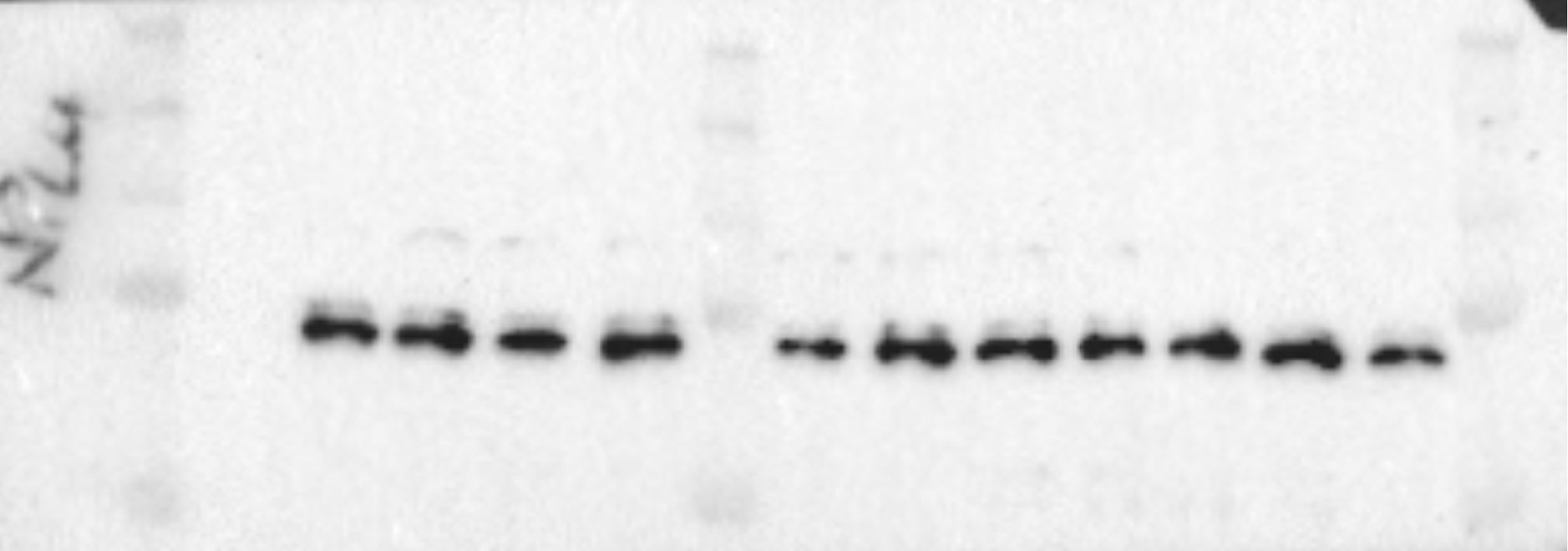

Supplement: Figure 3—source data 1. [file elife-92409-fig3-data1.zip › Figure 3-source data 1/Uncropped Originals /Originals merged with marker /PanelA - NPL4 input blot.tif]

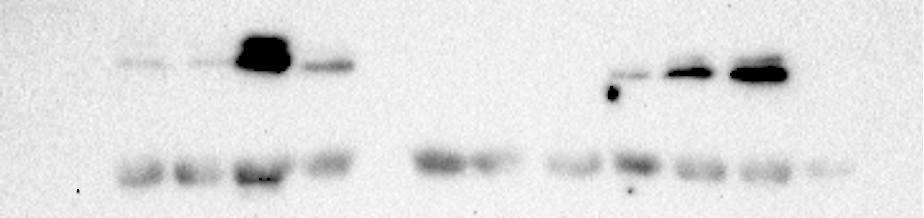

Supplement: Figure 3—source data 1. [file elife-92409-fig3-data1.zip › Figure 3-source data 1/Uncropped Originals /PanelA - NPL4 IP blot.tif]

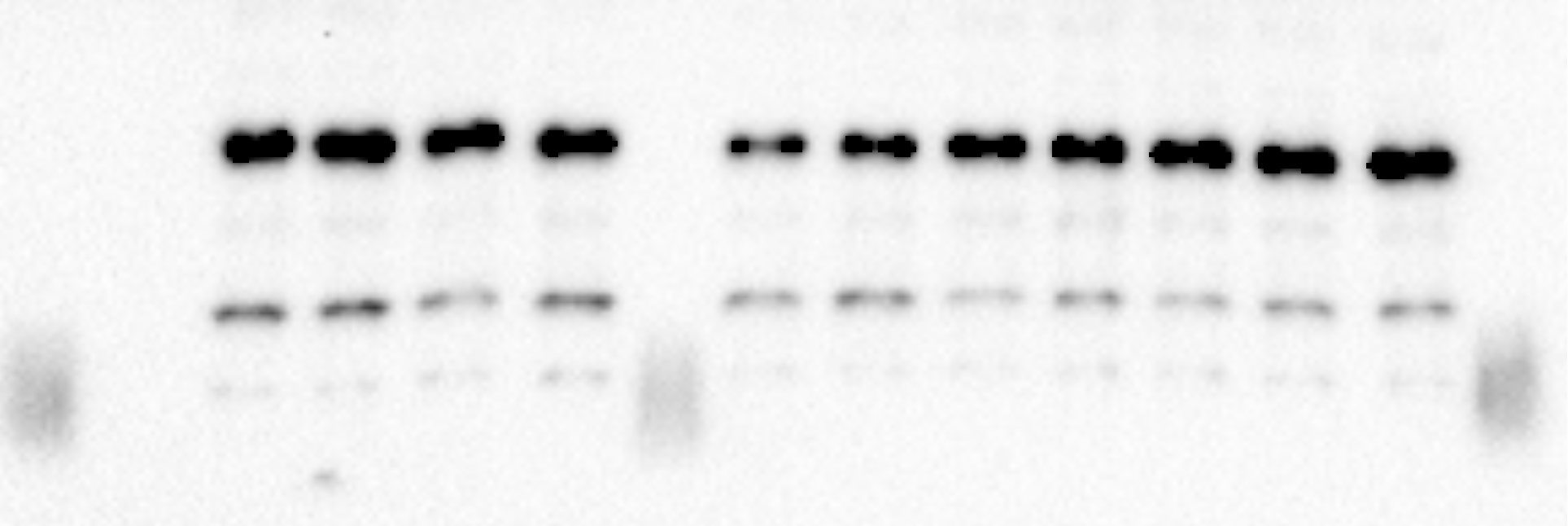

Supplement: Figure 3—source data 1. [file elife-92409-fig3-data1.zip › Figure 3-source data 1/Uncropped Originals /PanelA - UFD1 input blot.tif]

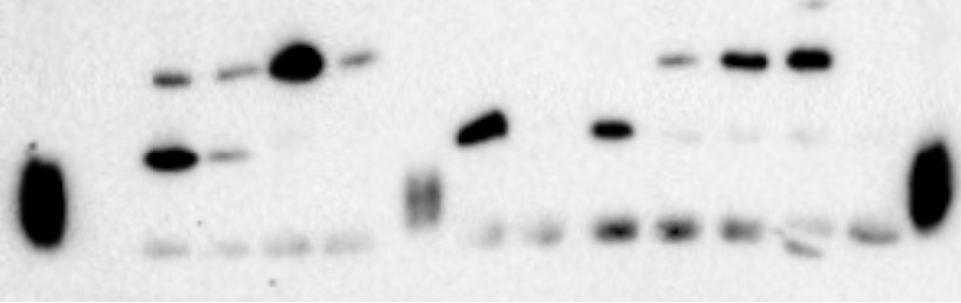

Supplement: Figure 3—source data 1. [file elife-92409-fig3-data1.zip › Figure 3-source data 1/Uncropped Originals /PanelA - UFD1 IP blot.tif]

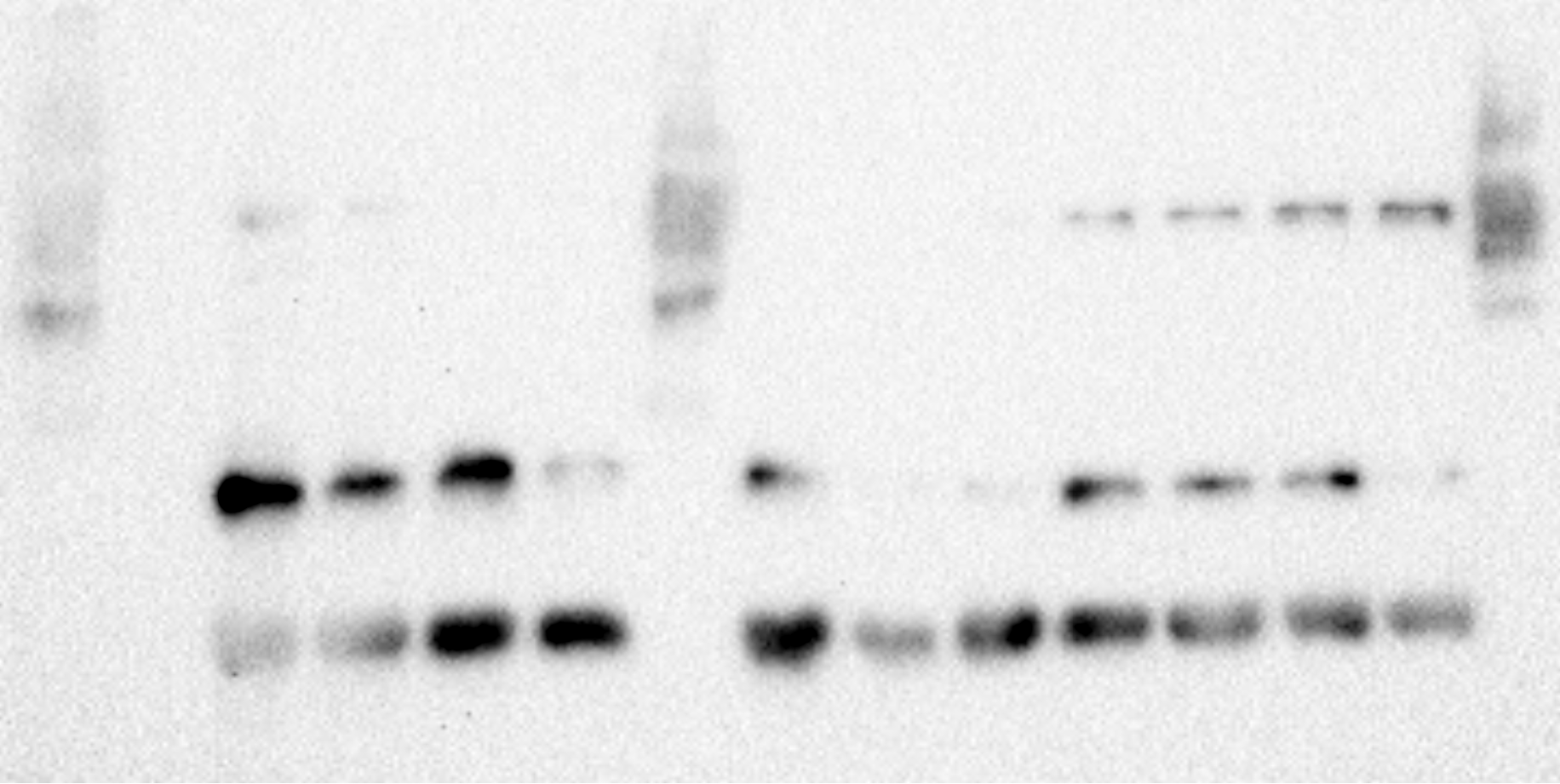

Supplement: Figure 3—source data 1. [file elife-92409-fig3-data1.zip › Figure 3-source data 1/Uncropped Originals /PanelA - UBXN7 IP blot.tif]

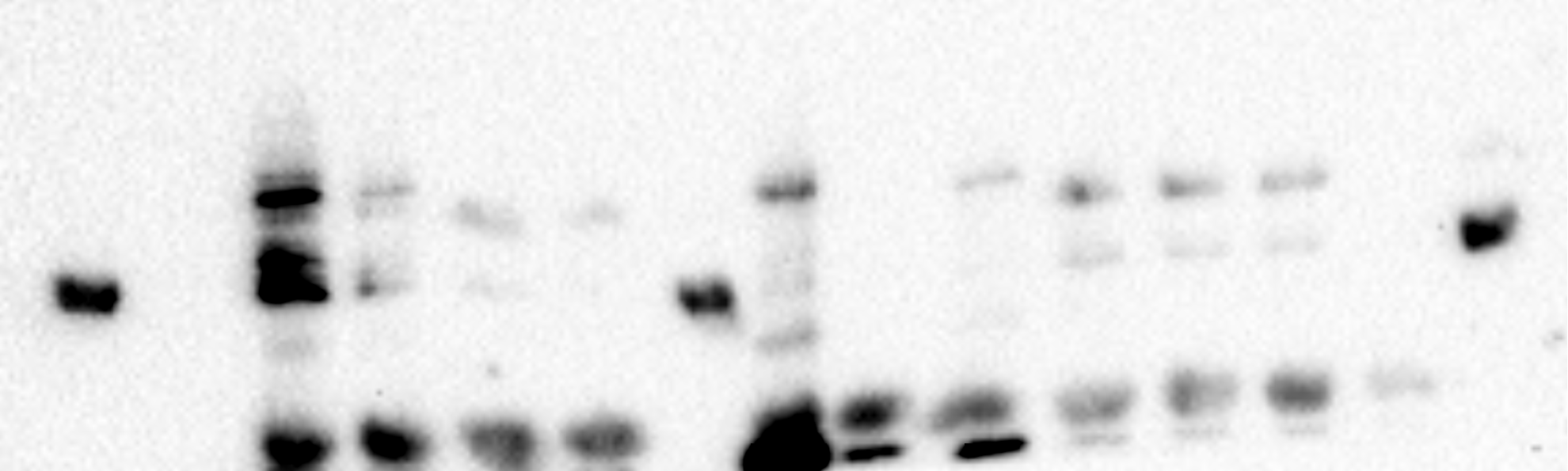

Supplement: Figure 3—source data 1. [file elife-92409-fig3-data1.zip › Figure 3-source data 1/Uncropped Originals /PanelA - FAF1 IP blot.tif]

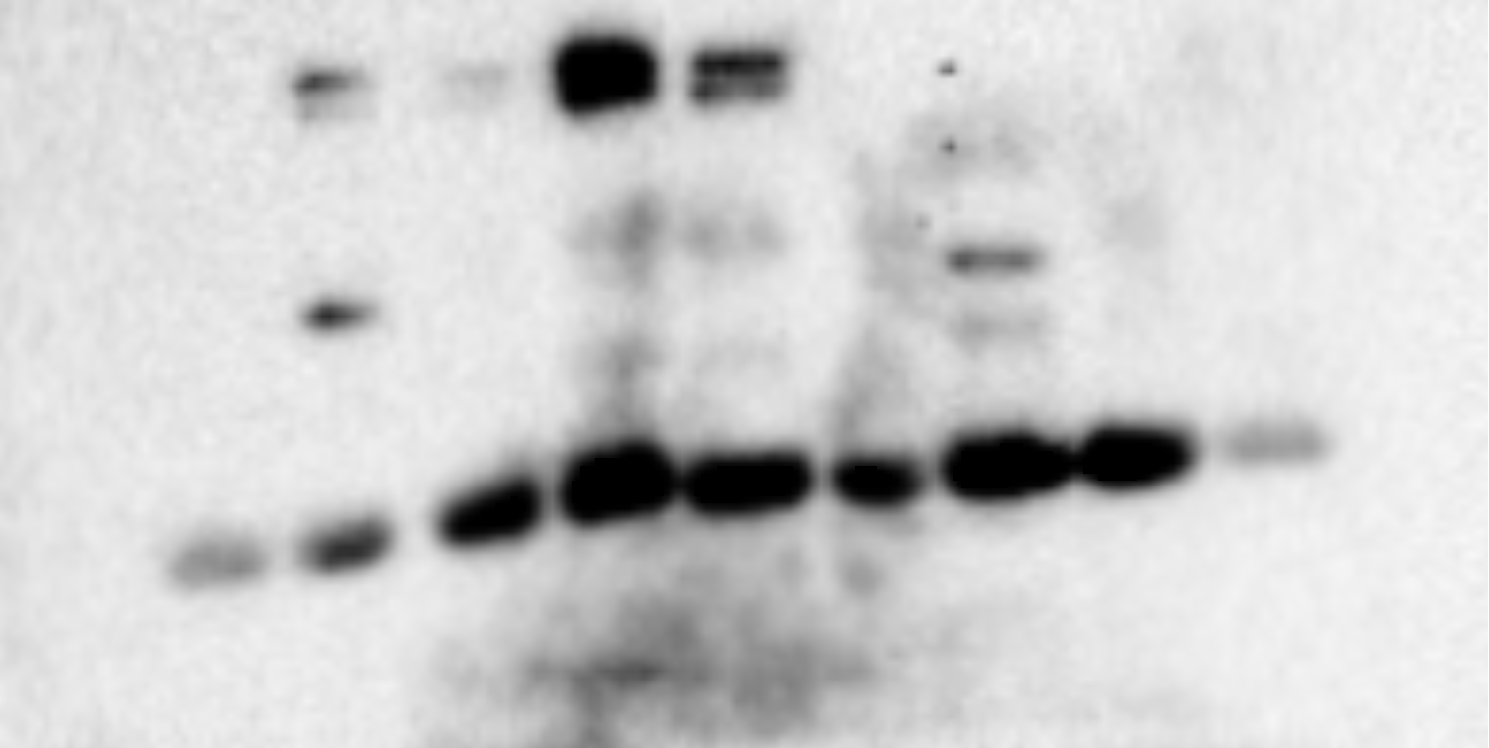

Supplement: Figure 3—source data 1. [file elife-92409-fig3-data1.zip › Figure 3-source data 1/Uncropped Originals /PanelA - UBXN2B IP blot.tif]

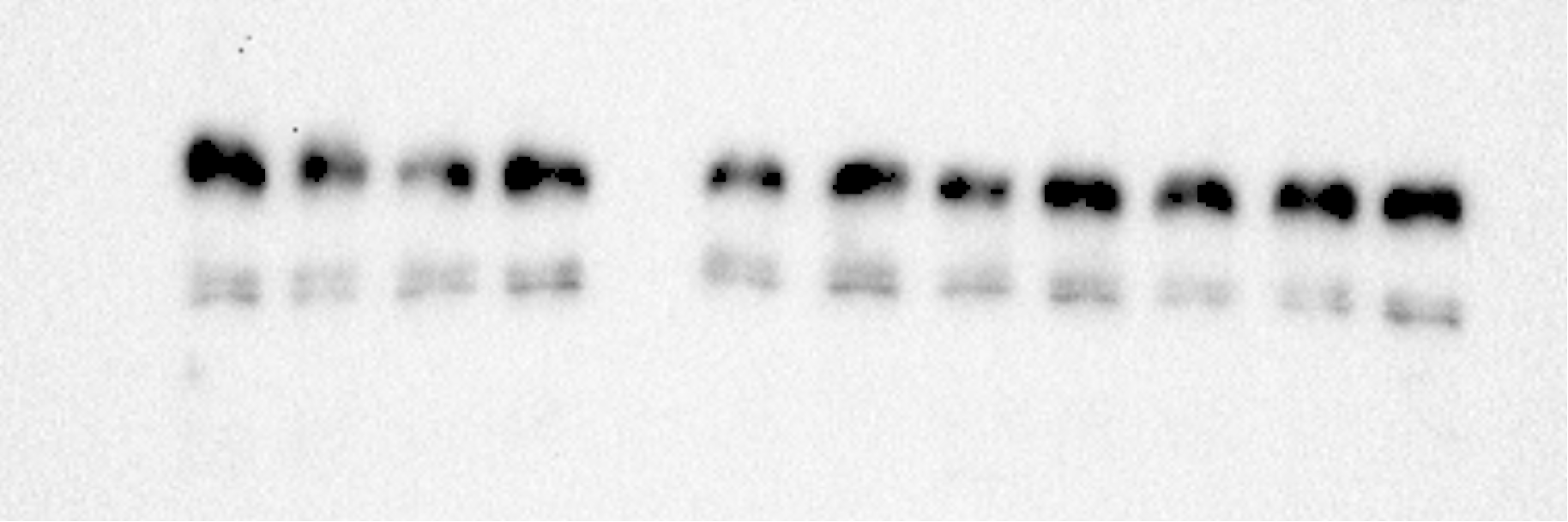

Supplement: Figure 3—source data 1. [file elife-92409-fig3-data1.zip › Figure 3-source data 1/Uncropped Originals /PanelA - UBXN7 input blot.tif]

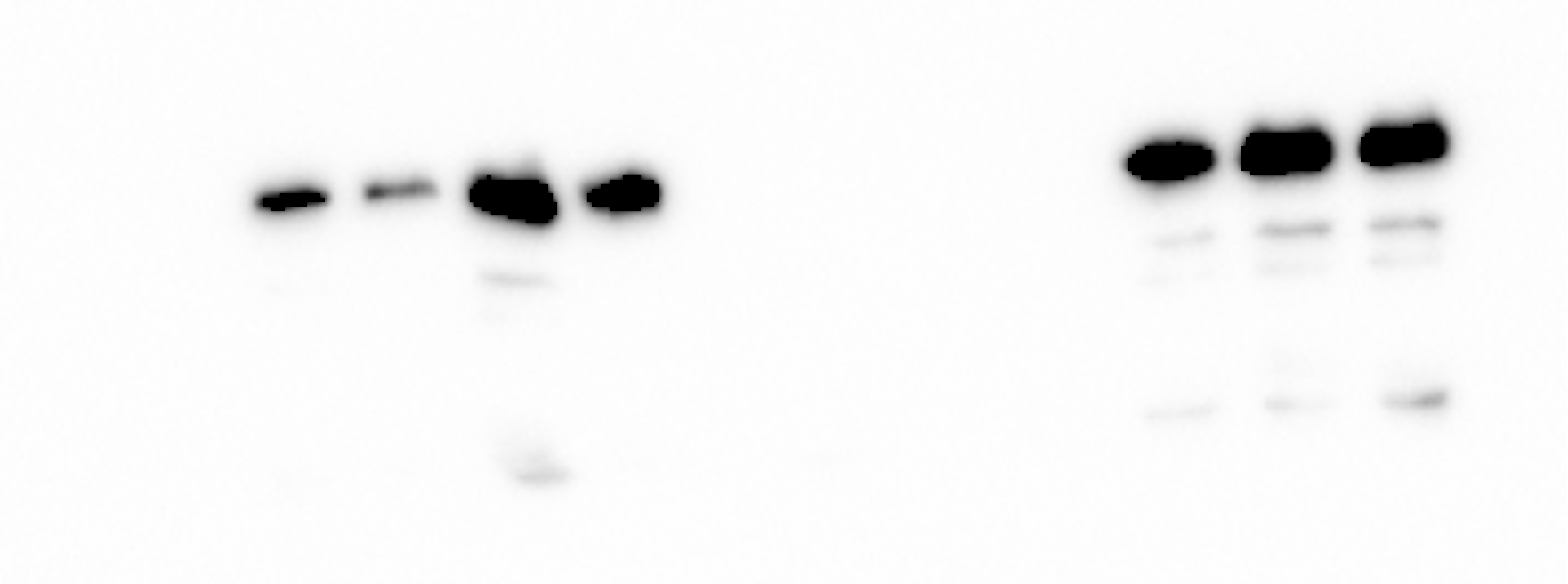

Supplement: Figure 3—source data 1. [file elife-92409-fig3-data1.zip › Figure 3-source data 1/Uncropped Originals /PanelA - p97 IP blot.tif]

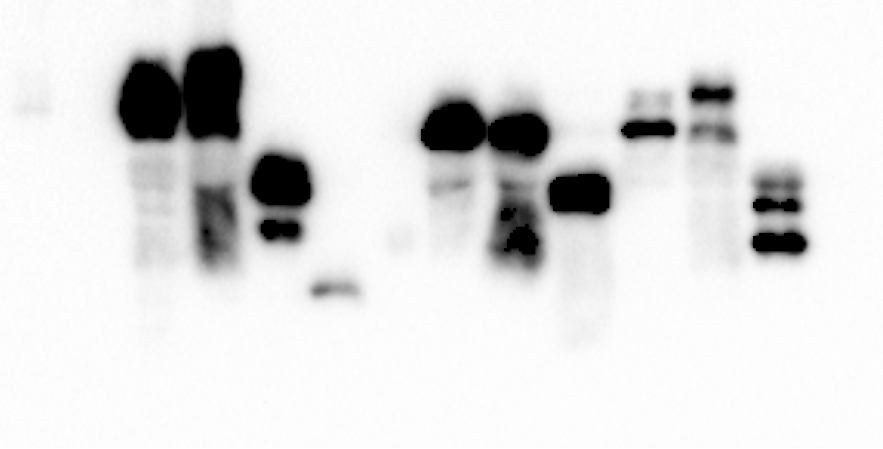

Supplement: Figure 3—source data 1. [file elife-92409-fig3-data1.zip › Figure 3-source data 1/Uncropped Originals /PanelA - Flag IP blot.tif]

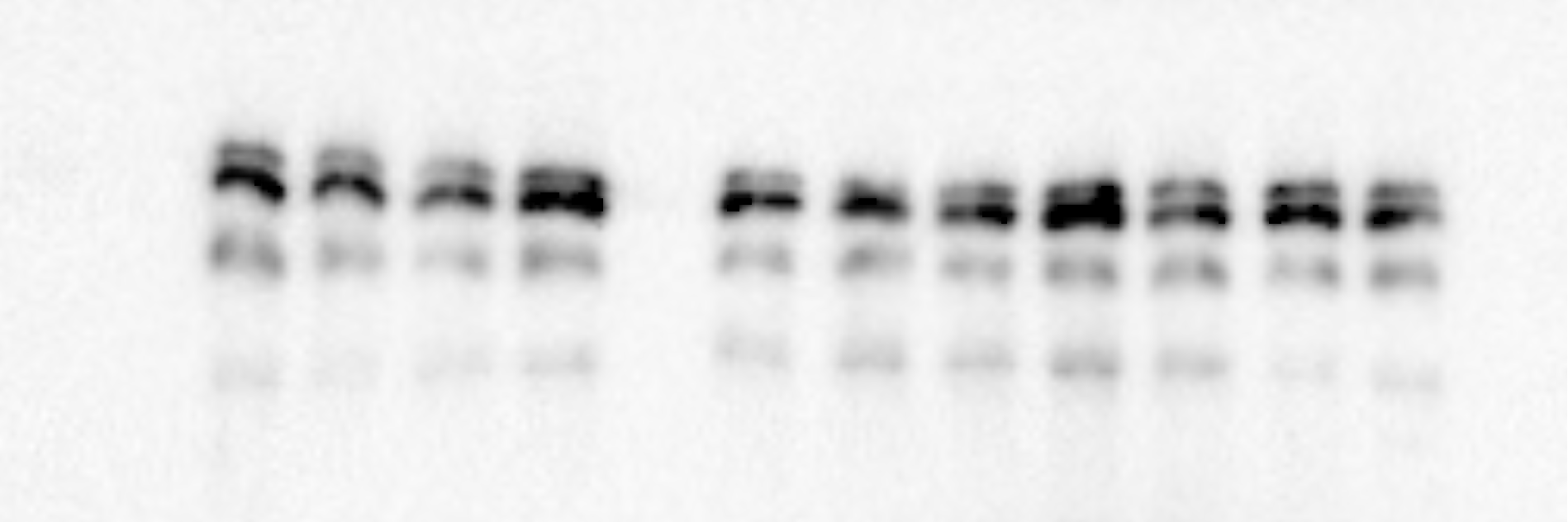

Supplement: Figure 3—source data 1. [file elife-92409-fig3-data1.zip › Figure 3-source data 1/Uncropped Originals /PanelA - FAF1 input blot.tif]

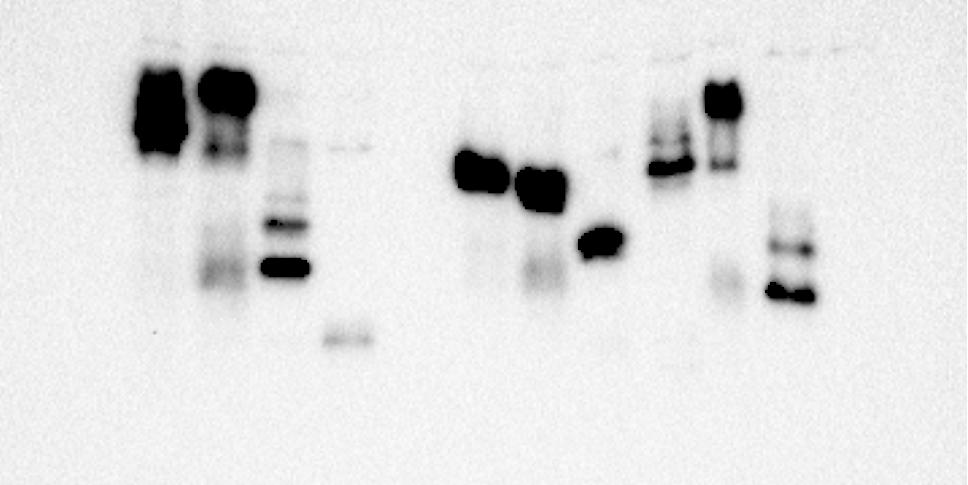

Supplement: Figure 3—source data 1. [file elife-92409-fig3-data1.zip › Figure 3-source data 1/Uncropped Originals /PanelA - Flag input blot.tif]

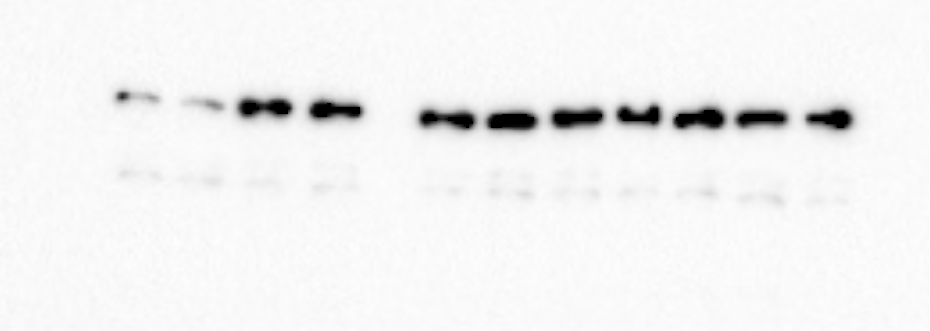

Supplement: Figure 3—source data 1. [file elife-92409-fig3-data1.zip › Figure 3-source data 1/Uncropped Originals /PanelA - p97 input blot.tif]

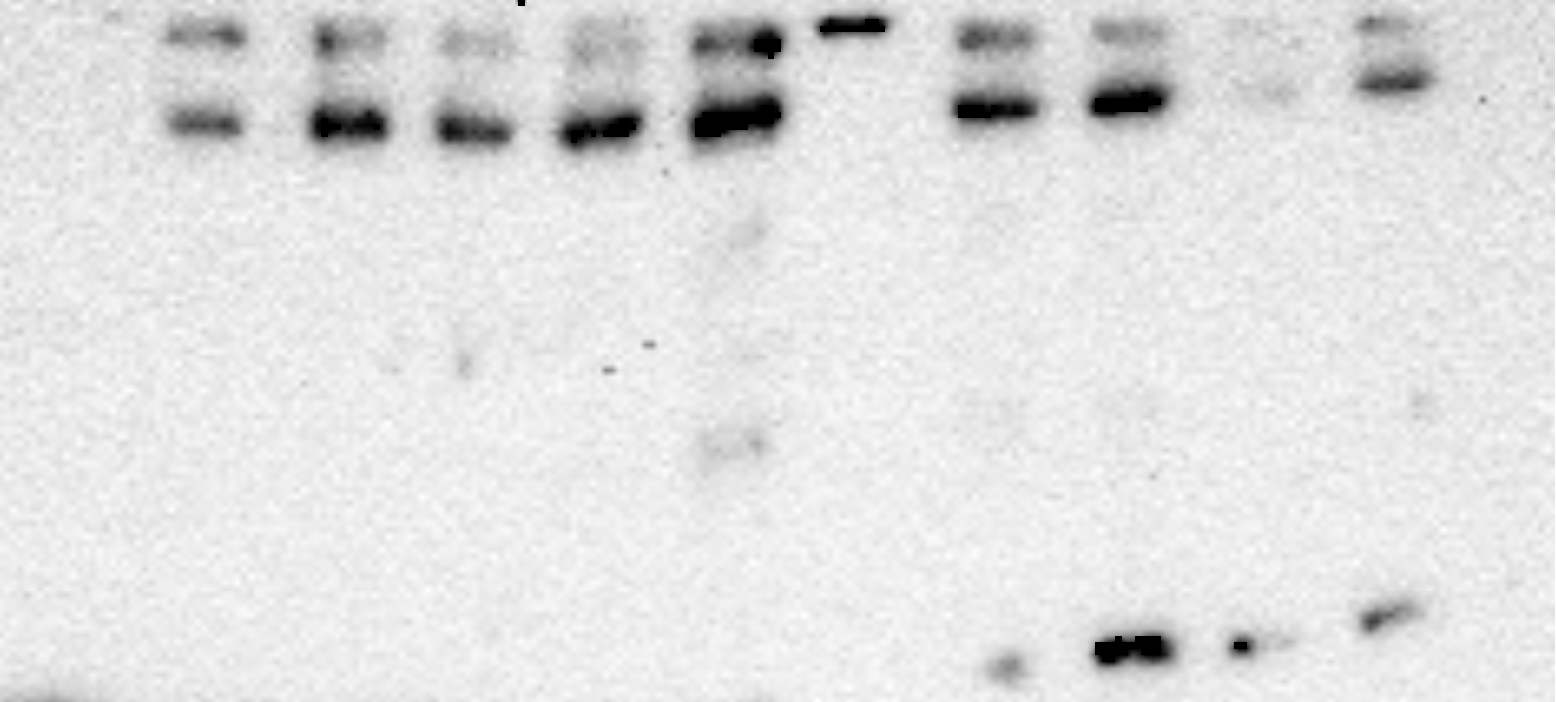

Supplement: Figure 3—source data 1. [file elife-92409-fig3-data1.zip › Figure 3-source data 1/Uncropped Originals /PanelA - UBXN2B input blot.tif]

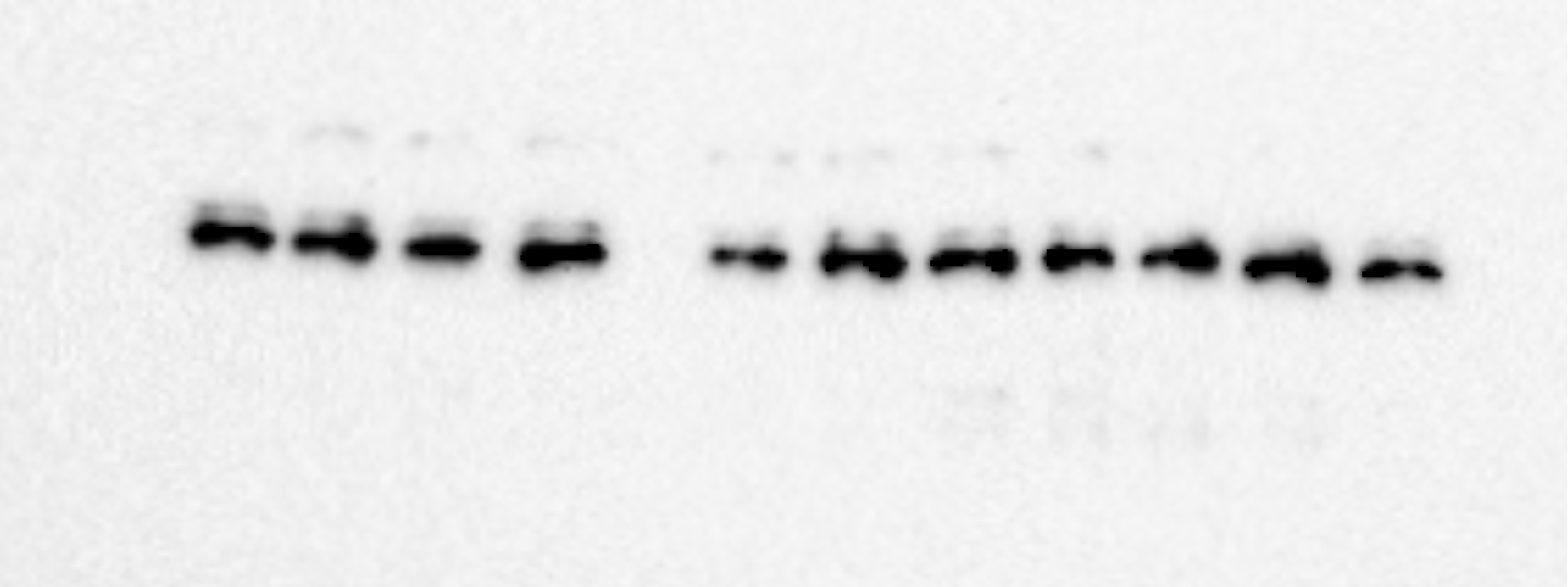

Supplement: Figure 3—source data 1. [file elife-92409-fig3-data1.zip › Figure 3-source data 1/Uncropped Originals /PanelA - NPL4 input blot.tif]

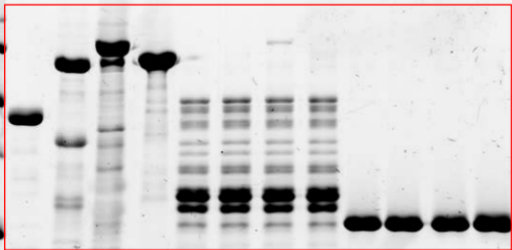

Supplement: Figure 3—source data 2. [file elife-92409-fig3-data2.zip › Figure 3-source data 2/Uncropped Labelled/Panel B - TCE.pdf]

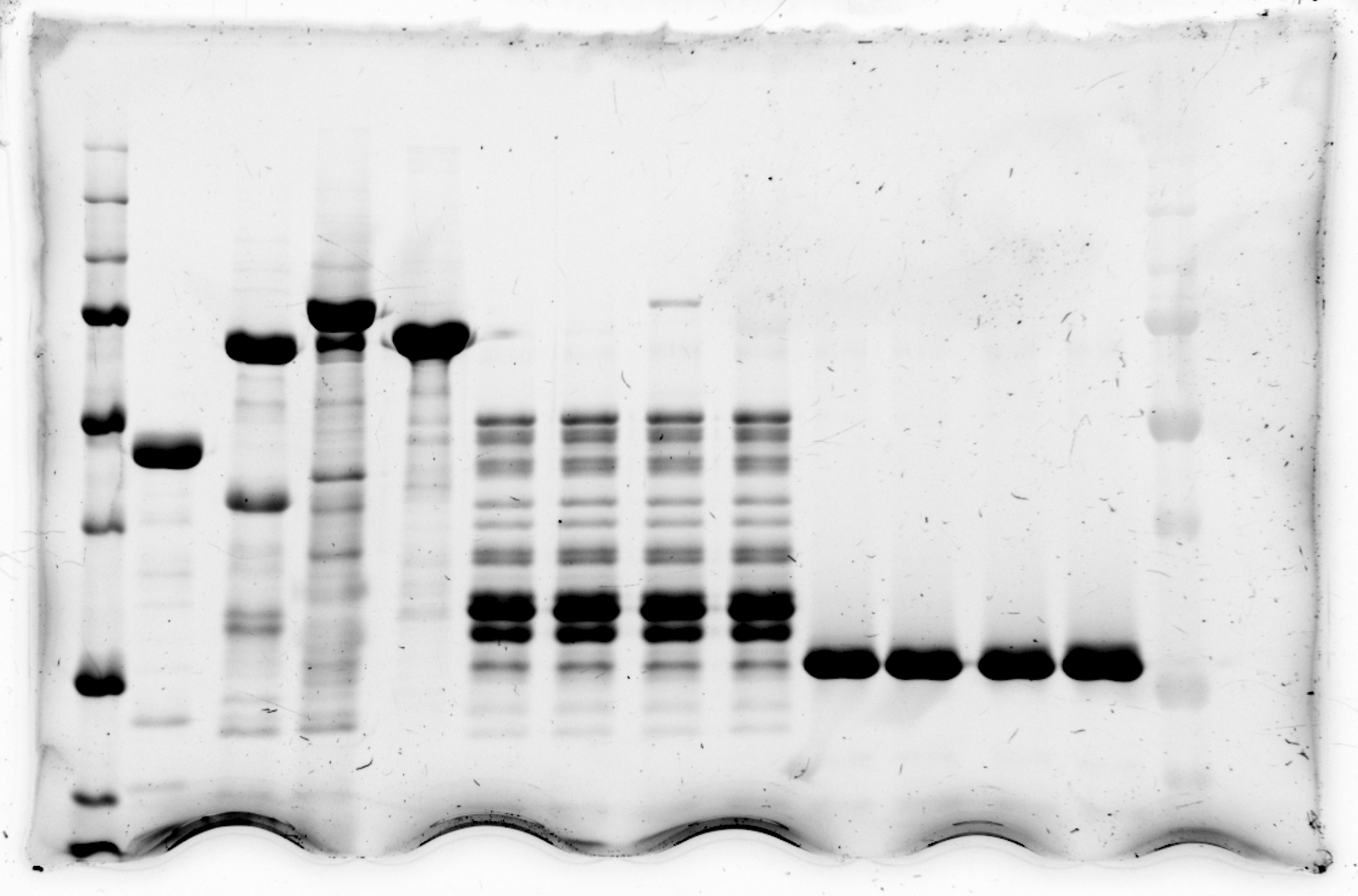

Supplement: Figure 3—source data 2. [file elife-92409-fig3-data2.zip › Figure 3-source data 2/Uncropped Originals/Panel B - TCE.tif]

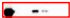

Supplement: Figure 3—source data 3. [file elife-92409-fig3-data3.zip › Figure 3-source data 3/Uncropped Labelled/Panel C - FAF1 blot.pdf]

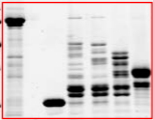

Supplement: Figure 3—source data 3. [file elife-92409-fig3-data3.zip › Figure 3-source data 3/Uncropped Labelled/Panel C - TCE.pdf]

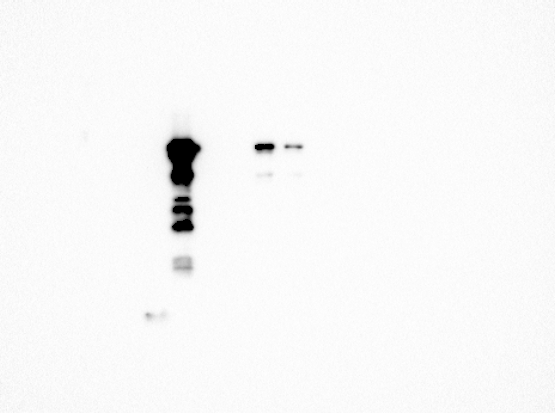

Supplement: Figure 3—source data 3. [file elife-92409-fig3-data3.zip › Figure 3-source data 3/Uncropped Originals/Panel C - FAF1 blot.tif]

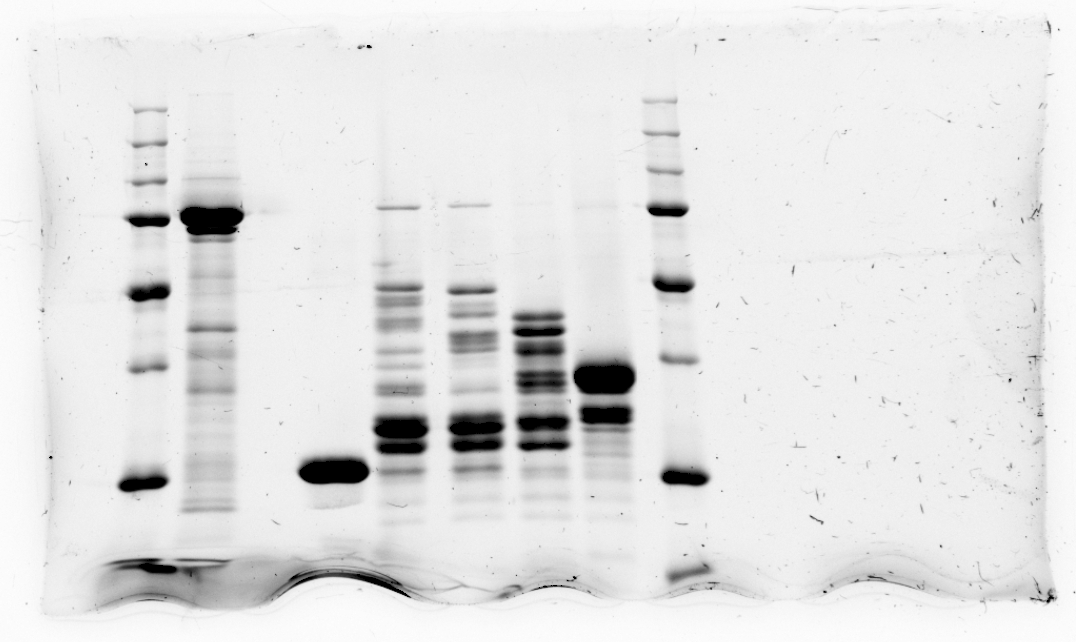

Supplement: Figure 3—source data 3. [file elife-92409-fig3-data3.zip › Figure 3-source data 3/Uncropped Originals/Panel C - TCE.tif]

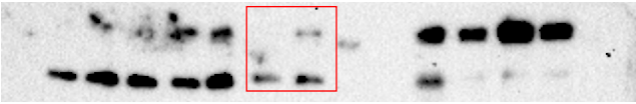

Supplement: Figure 3—figure supplement 1—source data 1. [file elife-92409-fig3-figsupp1-data1.zip › Figure 3-figure supplement 1-source data 1/Uncropped Labelled/PanelB UBXN2B blot.pdf]

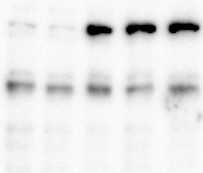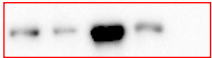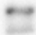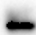

Supplement: Figure 3—figure supplement 1—source data 1. [file elife-92409-fig3-figsupp1-data1.zip › Figure 3-figure supplement 1-source data 1/Uncropped Labelled/PanelA p97 IP blot.pdf]

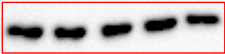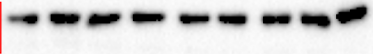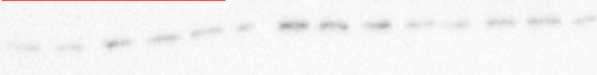

Supplement: Figure 3—figure supplement 1—source data 1. [file elife-92409-fig3-figsupp1-data1.zip › Figure 3-figure supplement 1-source data 1/Uncropped Labelled/PanelC UFD1 Input blot.pdf]

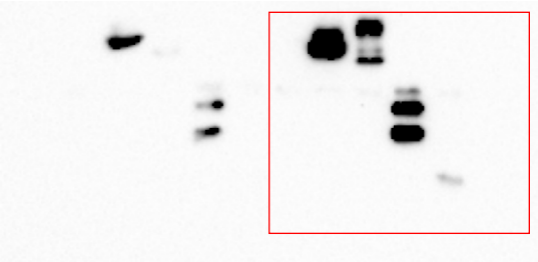

Supplement: Figure 3—figure supplement 1—source data 1. [file elife-92409-fig3-figsupp1-data1.zip › Figure 3-figure supplement 1-source data 1/Uncropped Labelled/PanelC Flag IP blot.pdf]

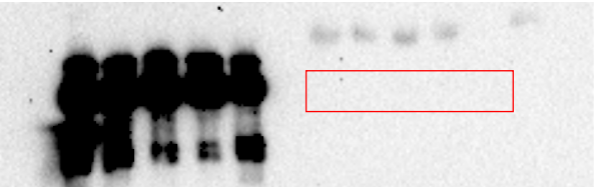

Supplement: Figure 3—figure supplement 1—source data 1. [file elife-92409-fig3-figsupp1-data1.zip › Figure 3-figure supplement 1-source data 1/Uncropped Labelled/PanelA p47 IP blot.pdf]

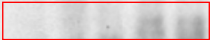

Supplement: Figure 3—figure supplement 1—source data 1. [file elife-92409-fig3-figsupp1-data1.zip › Figure 3-figure supplement 1-source data 1/Uncropped Labelled/PanelA UBXN2B Input blot.pdf]

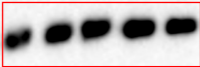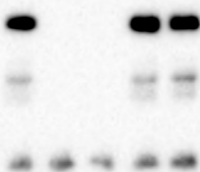

Supplement: Figure 3—figure supplement 1—source data 1. [file elife-92409-fig3-figsupp1-data1.zip › Figure 3-figure supplement 1-source data 1/Uncropped Labelled/PanelC p47 Input blot.pdf]

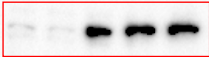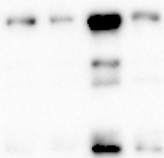

Supplement: Figure 3—figure supplement 1—source data 1. [file elife-92409-fig3-figsupp1-data1.zip › Figure 3-figure supplement 1-source data 1/Uncropped Labelled/PanelA p97 Input blot.pdf]

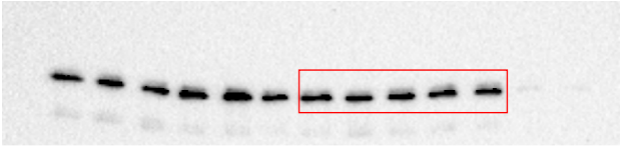

Supplement: Figure 3—figure supplement 1—source data 1. [file elife-92409-fig3-figsupp1-data1.zip › Figure 3-figure supplement 1-source data 1/Uncropped Labelled/PanelC NPL4 Input blot.pdf]

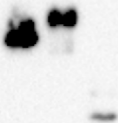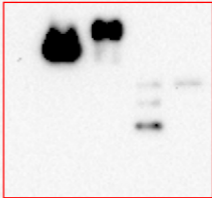

Supplement: Figure 3—figure supplement 1—source data 1. [file elife-92409-fig3-figsupp1-data1.zip › Figure 3-figure supplement 1-source data 1/Uncropped Labelled/PanelC Flag Input blot.pdf]

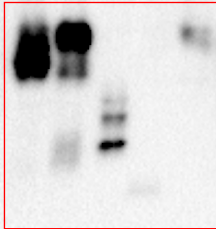

Supplement: Figure 3—figure supplement 1—source data 1. [file elife-92409-fig3-figsupp1-data1.zip › Figure 3-figure supplement 1-source data 1/Uncropped Labelled/PanelA - Flag Input blot.pdf]

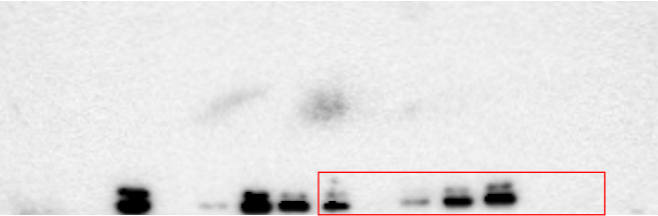

Supplement: Figure 3—figure supplement 1—source data 1. [file elife-92409-fig3-figsupp1-data1.zip › Figure 3-figure supplement 1-source data 1/Uncropped Labelled/PanelC NPL4 IP blot.pdf]

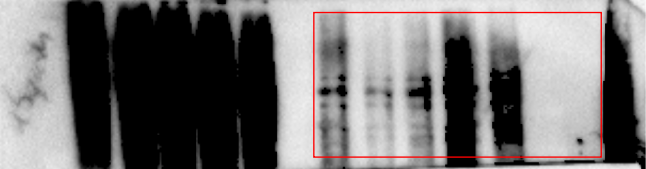

Supplement: Figure 3—figure supplement 1—source data 1. [file elife-92409-fig3-figsupp1-data1.zip › Figure 3-figure supplement 1-source data 1/Uncropped Labelled/PanelC Ubiquitin IP blot.pdf]

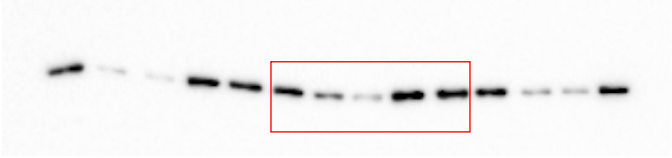

Supplement: Figure 3—figure supplement 1—source data 1. [file elife-92409-fig3-figsupp1-data1.zip › Figure 3-figure supplement 1-source data 1/Uncropped Labelled/PanelC p97 Input blot.pdf]

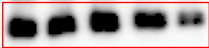

Supplement: Figure 3—figure supplement 1—source data 1. [file elife-92409-fig3-figsupp1-data1.zip › Figure 3-figure supplement 1-source data 1/Uncropped Labelled/PanelA p47 Input blot.pdf]

100

100

100

100

100

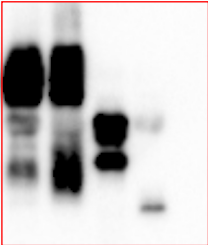

Supplement: Figure 3—figure supplement 1—source data 1. [file elife-92409-fig3-figsupp1-data1.zip › Figure 3-figure supplement 1-source data 1/Uncropped Labelled/PanelA - Flag IP blot.pdf]

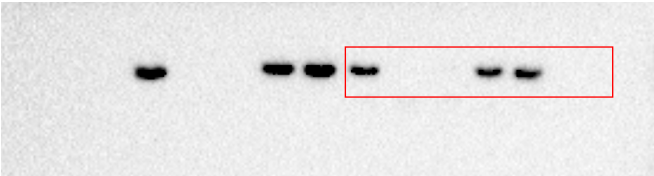

Supplement: Figure 3—figure supplement 1—source data 1. [file elife-92409-fig3-figsupp1-data1.zip › Figure 3-figure supplement 1-source data 1/Uncropped Labelled/PanelC UFD1 IP blot.pdf]

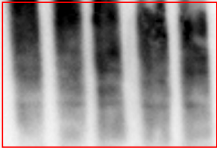

Supplement: Figure 3—figure supplement 1—source data 1. [file elife-92409-fig3-figsupp1-data1.zip › Figure 3-figure supplement 1-source data 1/Uncropped Labelled/PanelC Ubiquitin Input blot.pdf]

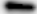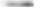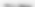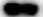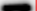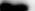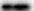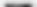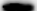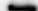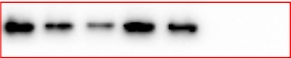

Supplement: Figure 3—figure supplement 1—source data 1. [file elife-92409-fig3-figsupp1-data1.zip › Figure 3-figure supplement 1-source data 1/Uncropped Labelled/PanelC p97 IP blot.pdf]

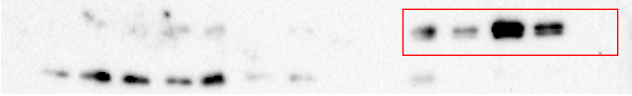

Supplement: Figure 3—figure supplement 1—source data 1. [file elife-92409-fig3-figsupp1-data1.zip › Figure 3-figure supplement 1-source data 1/Uncropped Labelled/PanelA UBXN2B IP blot.pdf]

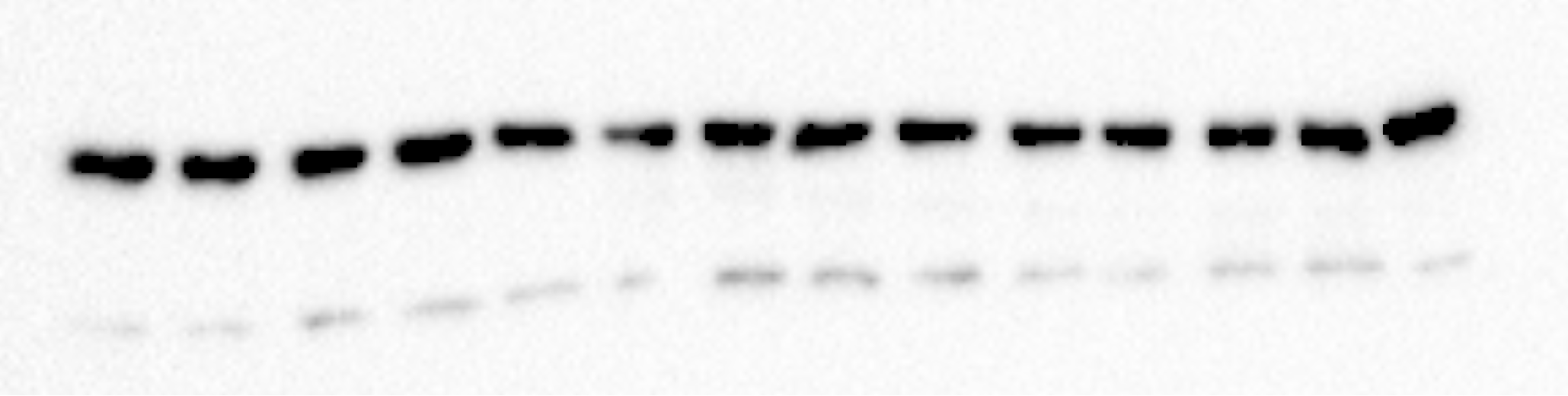

Supplement: Figure 3—figure supplement 1—source data 1. [file elife-92409-fig3-figsupp1-data1.zip › Figure 3-figure supplement 1-source data 1/Uncropped Originals /PanelC - UFD1 Input blot.tif]

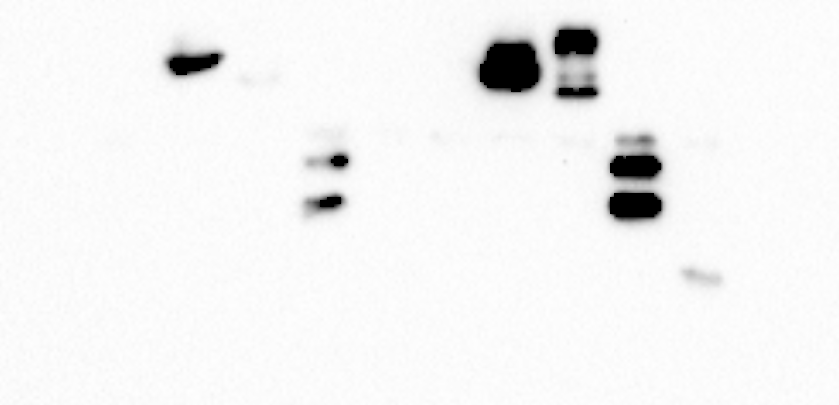

Supplement: Figure 3—figure supplement 1—source data 1. [file elife-92409-fig3-figsupp1-data1.zip › Figure 3-figure supplement 1-source data 1/Uncropped Originals /PanelC - Flag IP blot.tif]

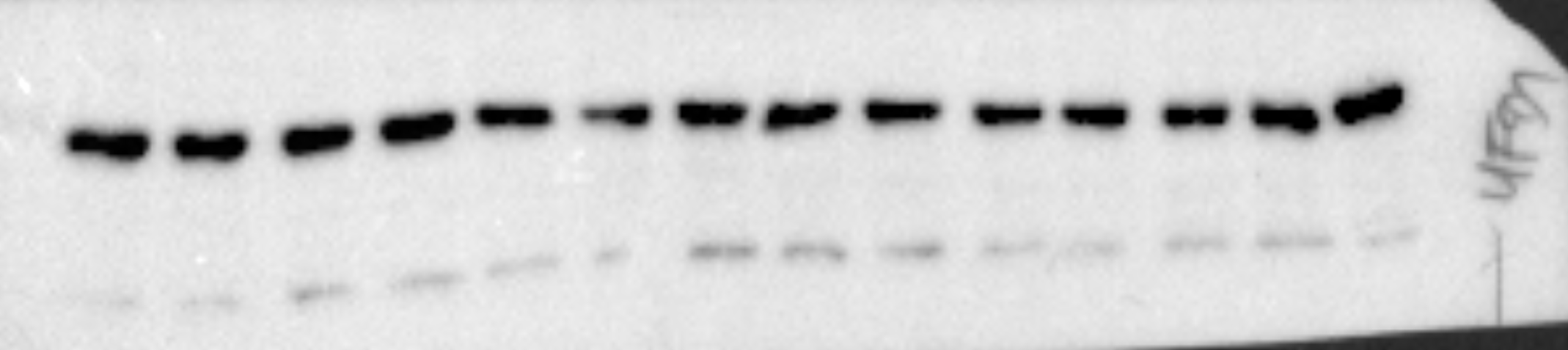

Supplement: Figure 3—figure supplement 1—source data 1. [file elife-92409-fig3-figsupp1-data1.zip › Figure 3-figure supplement 1-source data 1/Uncropped Originals /Uncropped merged with marker/PanelC - UFD1 Input blot.tif]

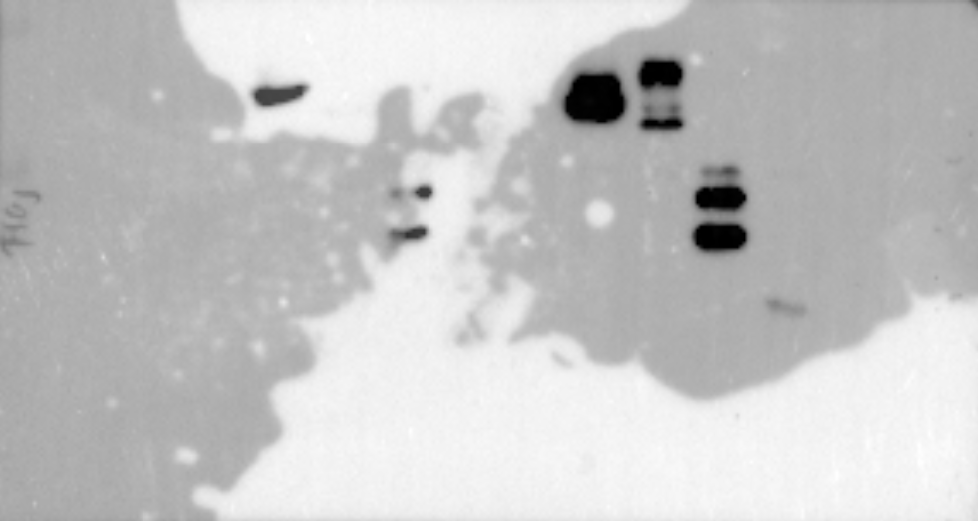

Supplement: Figure 3—figure supplement 1—source data 1. [file elife-92409-fig3-figsupp1-data1.zip › Figure 3-figure supplement 1-source data 1/Uncropped Originals /Uncropped merged with marker/PanelC - Flag IP blot.tif]

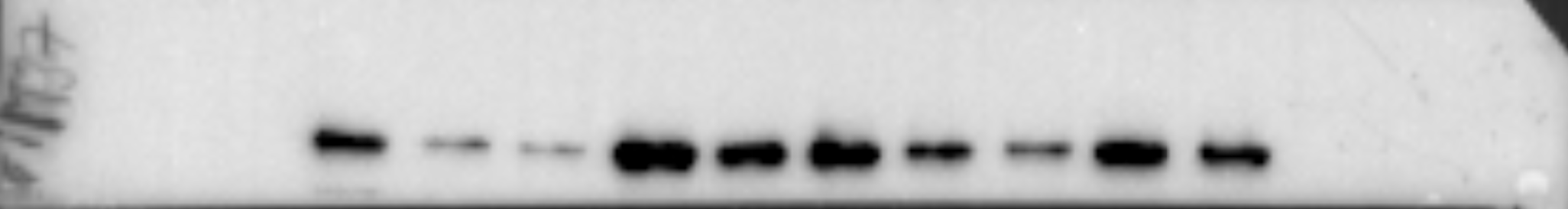

Supplement: Figure 3—figure supplement 1—source data 1. [file elife-92409-fig3-figsupp1-data1.zip › Figure 3-figure supplement 1-source data 1/Uncropped Originals /Uncropped merged with marker/PanelC - p97 IP.tif]

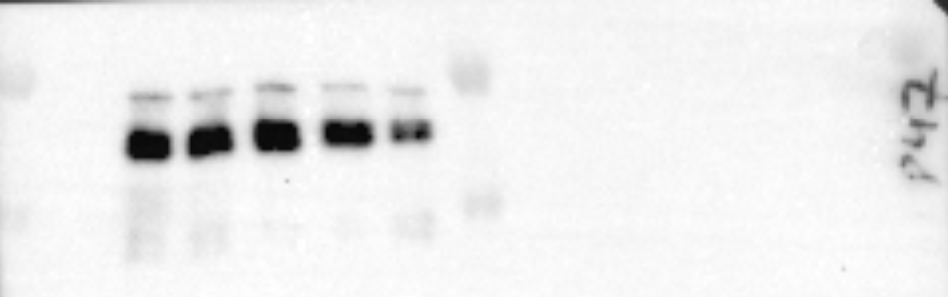

Supplement: Figure 3—figure supplement 1—source data 1. [file elife-92409-fig3-figsupp1-data1.zip › Figure 3-figure supplement 1-source data 1/Uncropped Originals /Uncropped merged with marker/PanelA - p47 Input blot.tif]

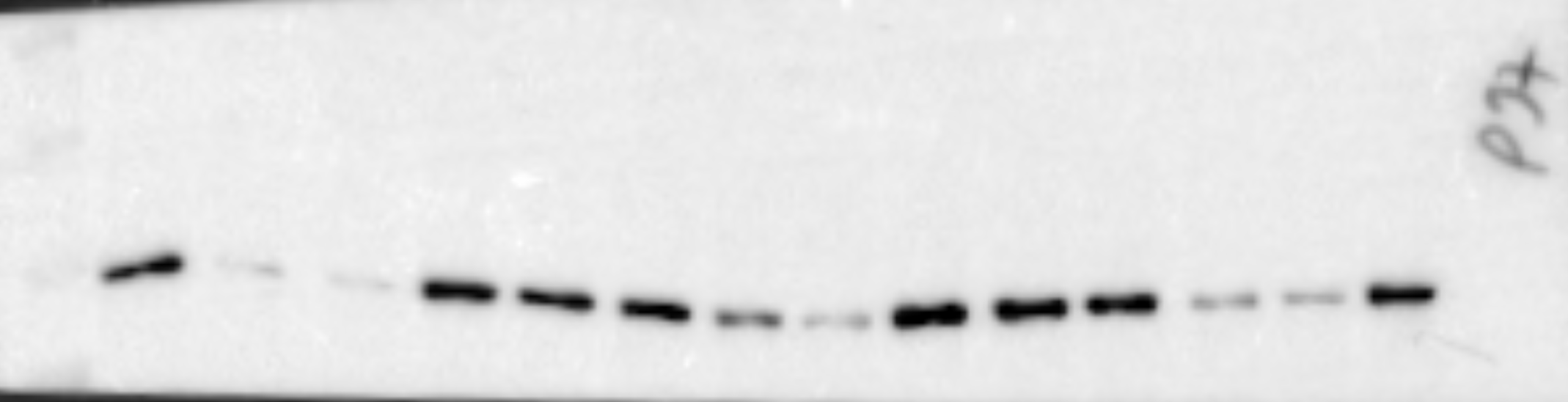

Supplement: Figure 3—figure supplement 1—source data 1. [file elife-92409-fig3-figsupp1-data1.zip › Figure 3-figure supplement 1-source data 1/Uncropped Originals /Uncropped merged with marker/PanelC - p97 Input blot.tif]

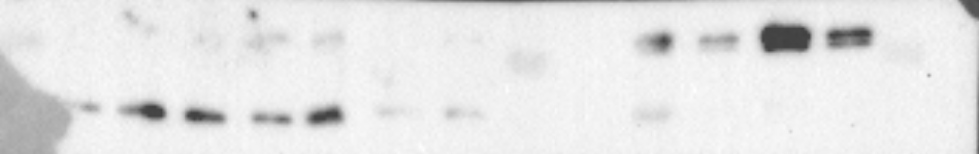

Supplement: Figure 3—figure supplement 1—source data 1. [file elife-92409-fig3-figsupp1-data1.zip › Figure 3-figure supplement 1-source data 1/Uncropped Originals /Uncropped merged with marker/PanelA - UBXN2B IP blot.tif]

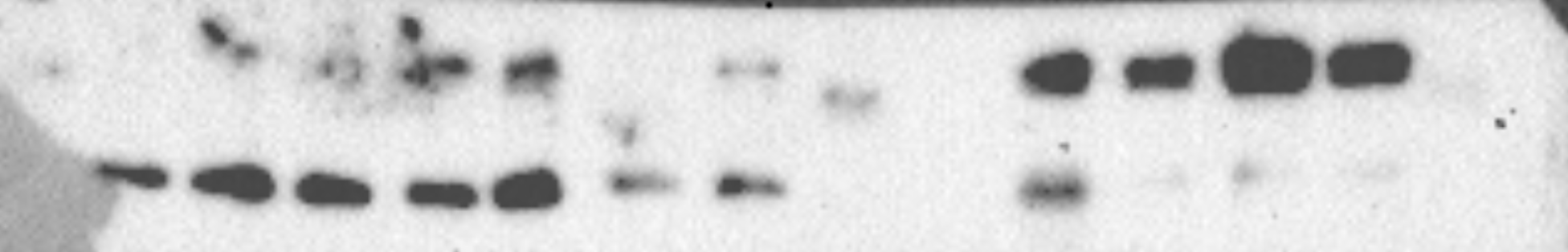

Supplement: Figure 3—figure supplement 1—source data 1. [file elife-92409-fig3-figsupp1-data1.zip › Figure 3-figure supplement 1-source data 1/Uncropped Originals /Uncropped merged with marker/PanelB - UBXN2B blot.tif]

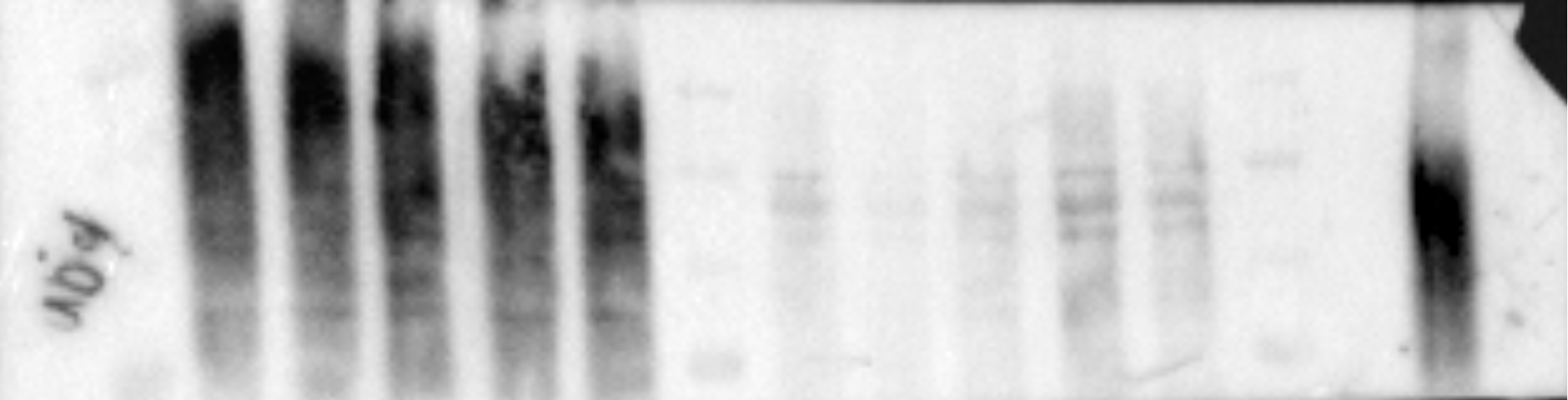

Supplement: Figure 3—figure supplement 1—source data 1. [file elife-92409-fig3-figsupp1-data1.zip › Figure 3-figure supplement 1-source data 1/Uncropped Originals /Uncropped merged with marker/PanelC - Ubiquitin Input blot.tif]

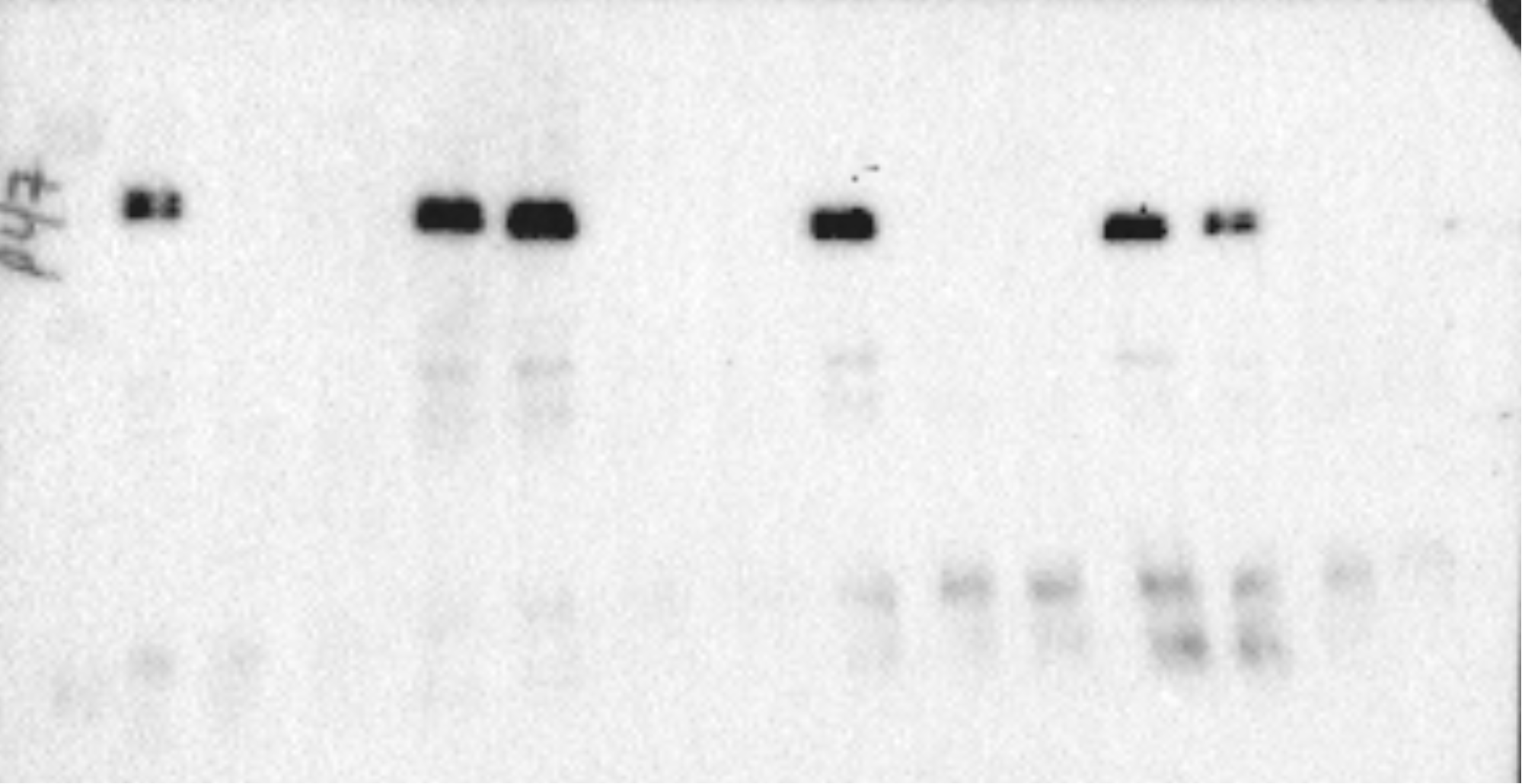

Supplement: Figure 3—figure supplement 1—source data 1. [file elife-92409-fig3-figsupp1-data1.zip › Figure 3-figure supplement 1-source data 1/Uncropped Originals /Uncropped merged with marker/PanelC - p47 IP blot.tif]

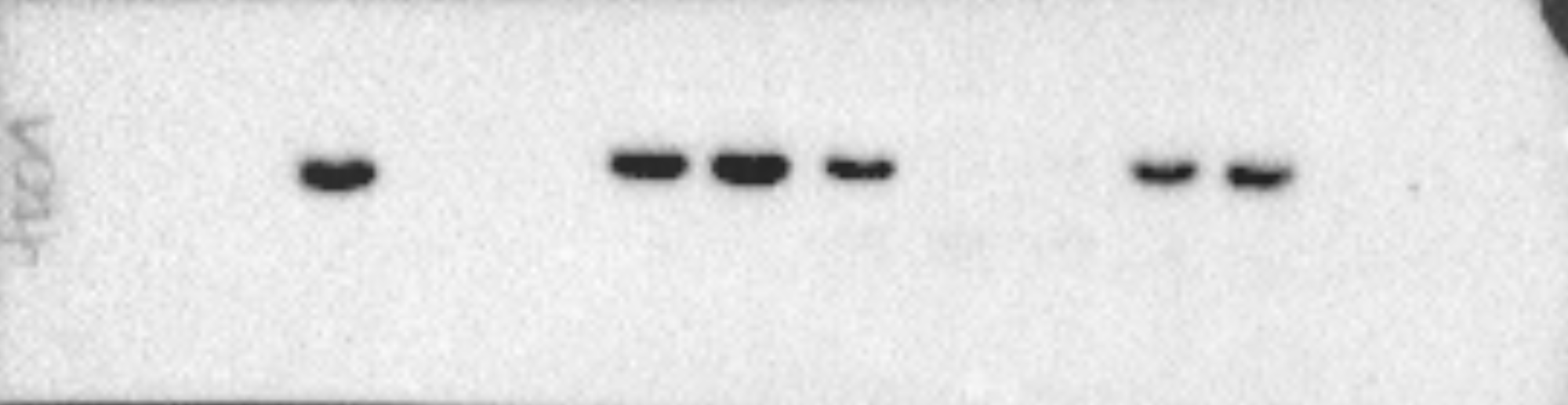

Supplement: Figure 3—figure supplement 1—source data 1. [file elife-92409-fig3-figsupp1-data1.zip › Figure 3-figure supplement 1-source data 1/Uncropped Originals /Uncropped merged with marker/PanelC - UFD1 IP blot.tif]

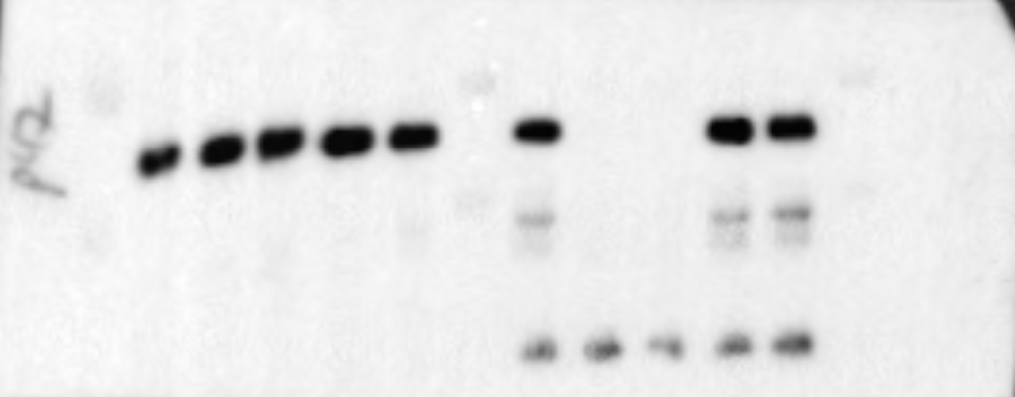

Supplement: Figure 3—figure supplement 1—source data 1. [file elife-92409-fig3-figsupp1-data1.zip › Figure 3-figure supplement 1-source data 1/Uncropped Originals /Uncropped merged with marker/PanelC - p47 Input blot.tif]

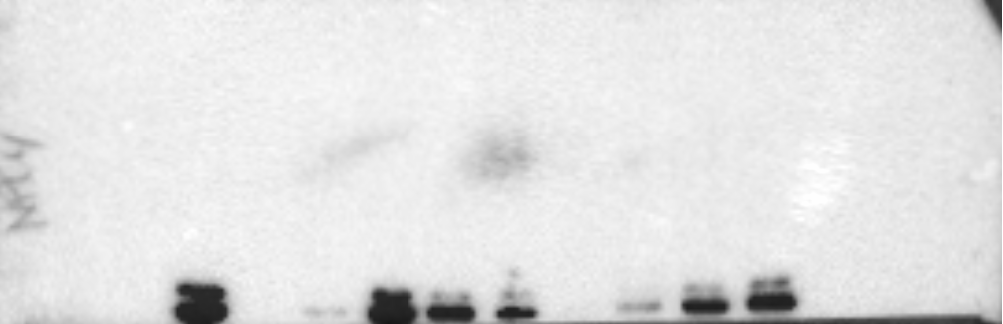

Supplement: Figure 3—figure supplement 1—source data 1. [file elife-92409-fig3-figsupp1-data1.zip › Figure 3-figure supplement 1-source data 1/Uncropped Originals /Uncropped merged with marker/PanelC - NPL4 IP blot .tif]

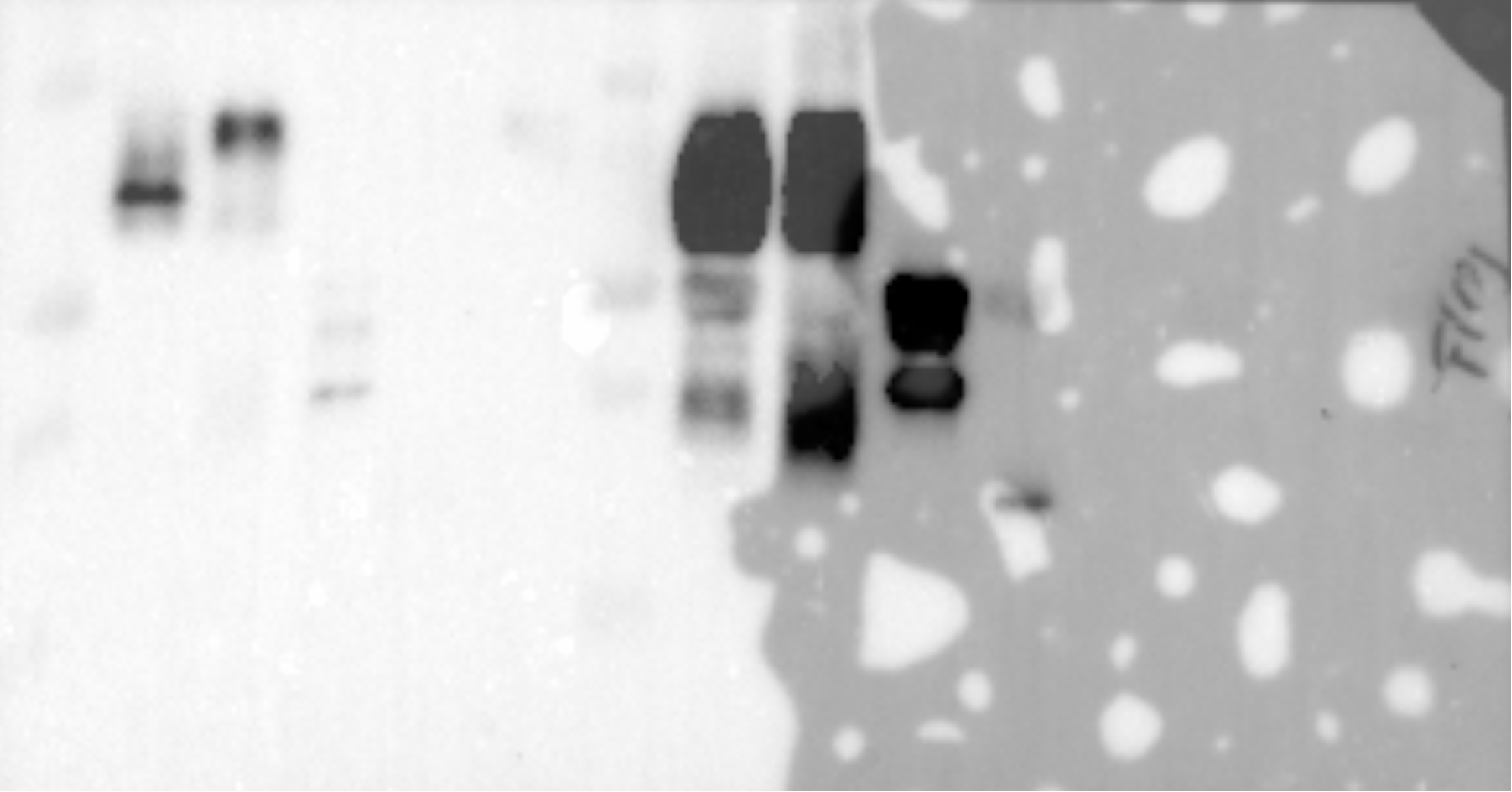

Supplement: Figure 3—figure supplement 1—source data 1. [file elife-92409-fig3-figsupp1-data1.zip › Figure 3-figure supplement 1-source data 1/Uncropped Originals /Uncropped merged with marker/PanelA - Flag IP blot.tif]

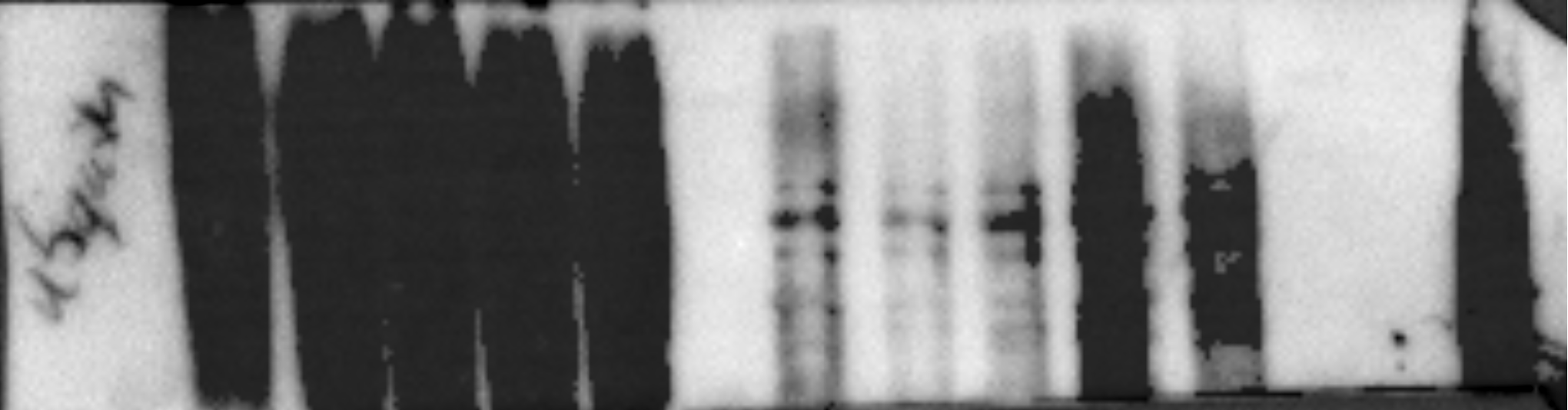

Supplement: Figure 3—figure supplement 1—source data 1. [file elife-92409-fig3-figsupp1-data1.zip › Figure 3-figure supplement 1-source data 1/Uncropped Originals /Uncropped merged with marker/PanelC - Ubiquitin IP blot.tif]

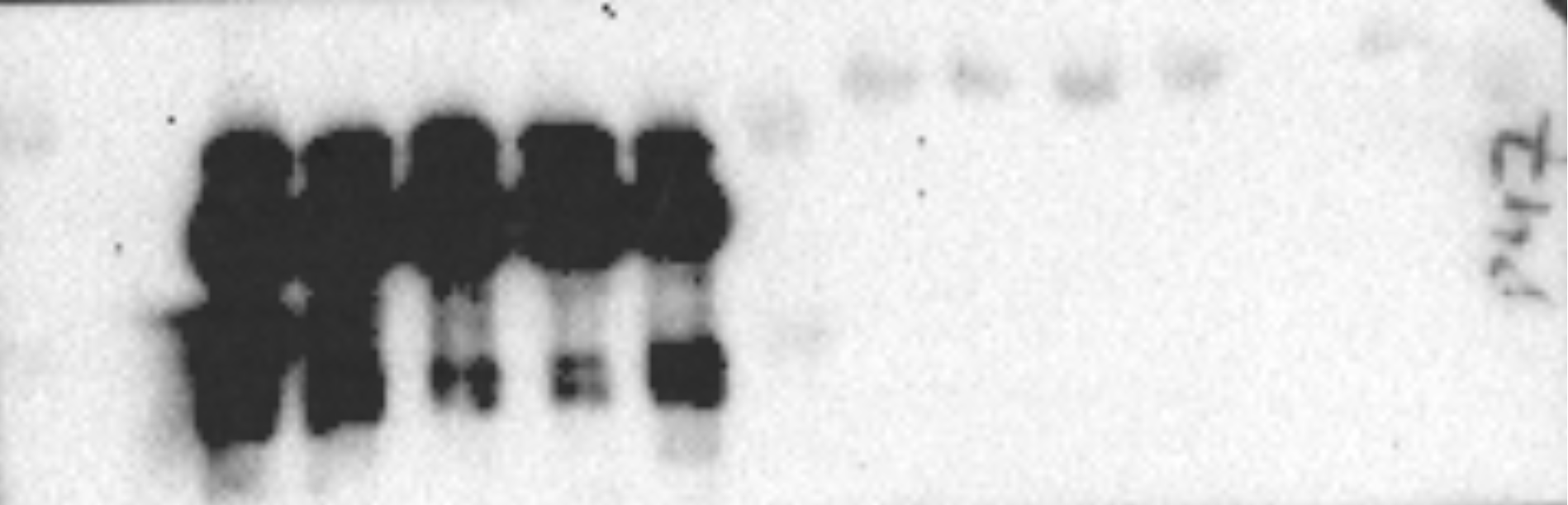

Supplement: Figure 3—figure supplement 1—source data 1. [file elife-92409-fig3-figsupp1-data1.zip › Figure 3-figure supplement 1-source data 1/Uncropped Originals /Uncropped merged with marker/PanelA - p47 IP blot.tif]
