# Supplementary material for: The FAM104 proteins VCF1/2 promote the nuclear localization of p97/VCP
Source: eLife. 2023 Sep 15;12:e92409. doi: 10.7554/eLife.92409 (PMC10541173; doi:10.7554/eLife.92409)

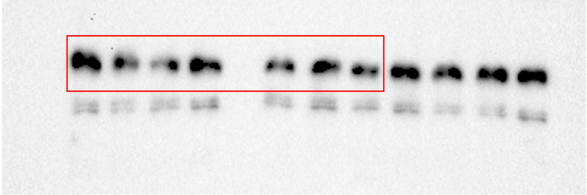

Supplement: Figure 3—source data 1. [file elife-92409-fig3-data1.zip › Figure 3-source data 1/Uncropped Labelled/PanelA - UBXN7 Input blot.pdf]
