# Supplementary figures and images for: The FAM104 proteins VCF1/2 promote the nuclear localization of p97/VCP (part 2 of 2)
Source: eLife. 2023 Sep 15;12:e92409. doi: 10.7554/eLife.92409 (PMC10541173; doi:10.7554/eLife.92409)

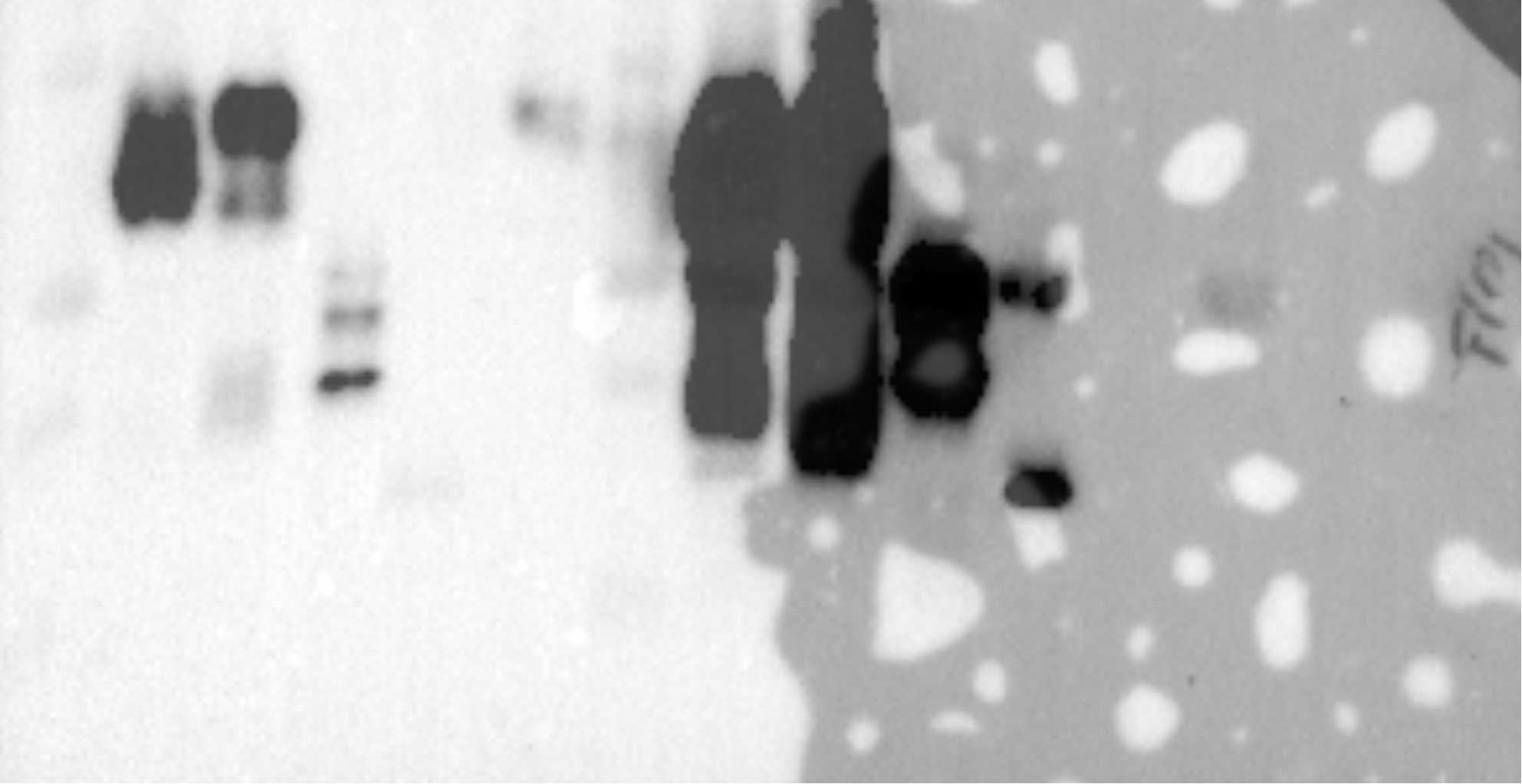

Supplement: Figure 3—figure supplement 1—source data 1. [file elife-92409-fig3-figsupp1-data1.zip › Figure 3-figure supplement 1-source data 1/Uncropped Originals /Uncropped merged with marker/PanelA - Flag Input blot.tif]

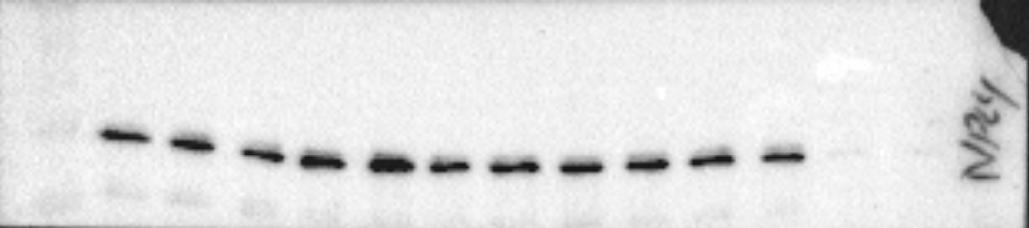

Supplement: Figure 3—figure supplement 1—source data 1. [file elife-92409-fig3-figsupp1-data1.zip › Figure 3-figure supplement 1-source data 1/Uncropped Originals /Uncropped merged with marker/PanelC - NPL4 Input blot.tif]

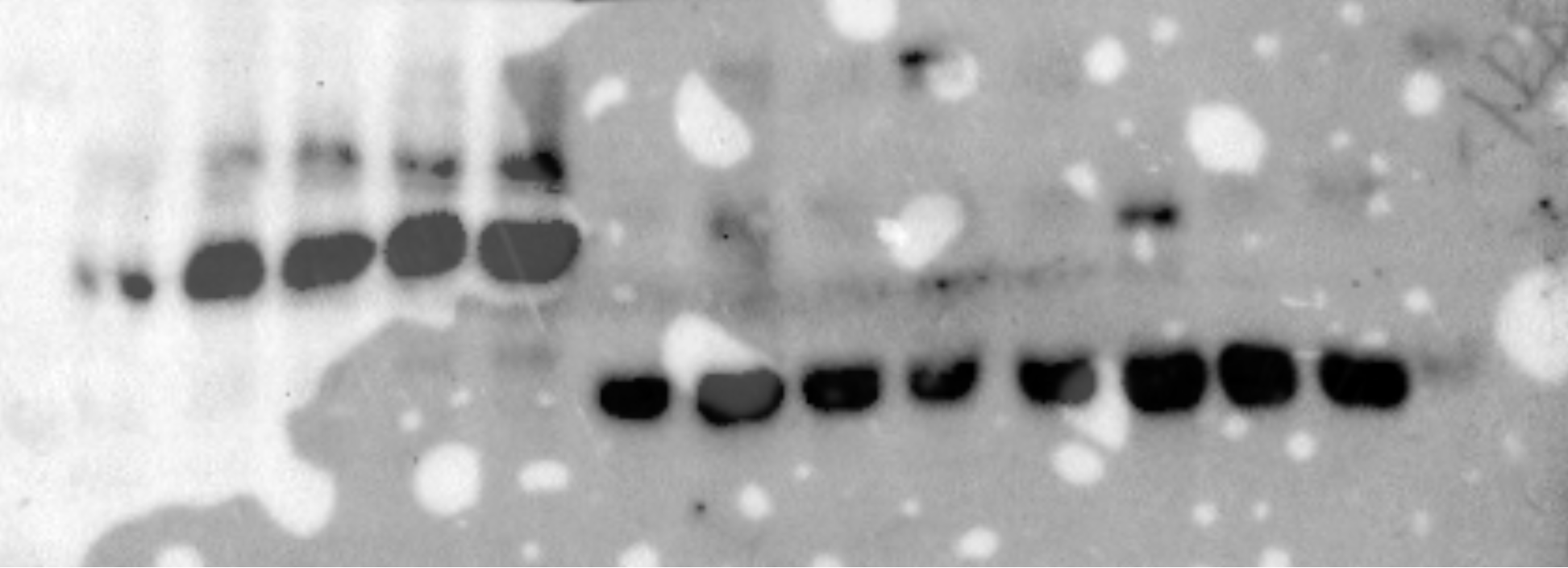

Supplement: Figure 3—figure supplement 1—source data 1. [file elife-92409-fig3-figsupp1-data1.zip › Figure 3-figure supplement 1-source data 1/Uncropped Originals /Uncropped merged with marker/PanelA - UBXN2B Input blot.tif]

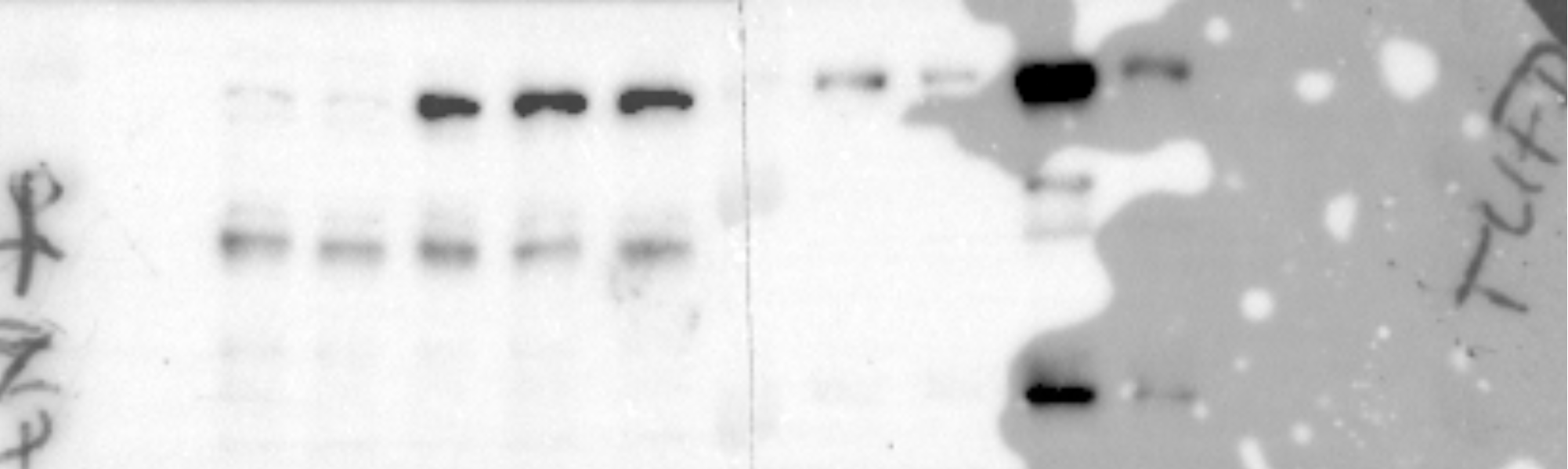

Supplement: Figure 3—figure supplement 1—source data 1. [file elife-92409-fig3-figsupp1-data1.zip › Figure 3-figure supplement 1-source data 1/Uncropped Originals /Uncropped merged with marker/PanelA - p97 Input and IP blot.tif]

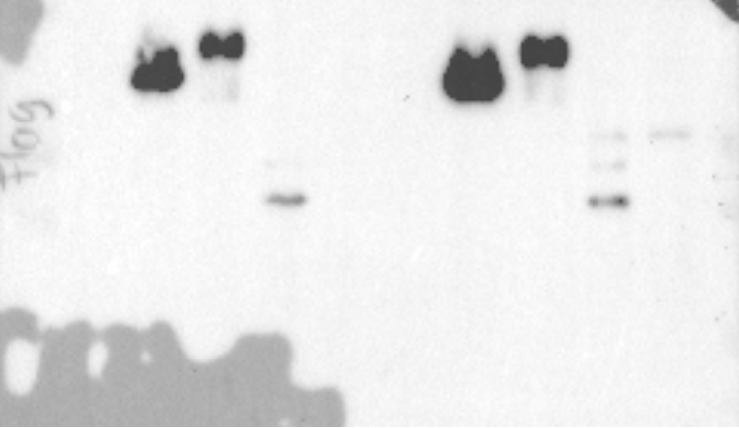

Supplement: Figure 3—figure supplement 1—source data 1. [file elife-92409-fig3-figsupp1-data1.zip › Figure 3-figure supplement 1-source data 1/Uncropped Originals /Uncropped merged with marker/PanelC - Flag Input blot.tif]

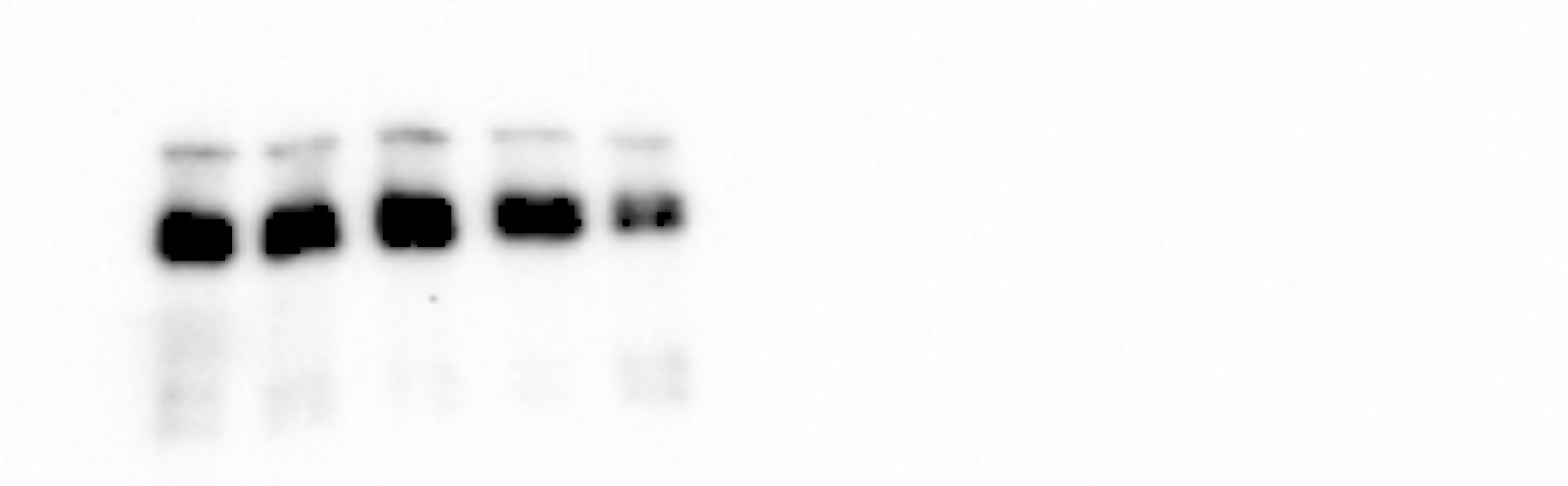

Supplement: Figure 3—figure supplement 1—source data 1. [file elife-92409-fig3-figsupp1-data1.zip › Figure 3-figure supplement 1-source data 1/Uncropped Originals /PanelA - p47 Input blot.tif]

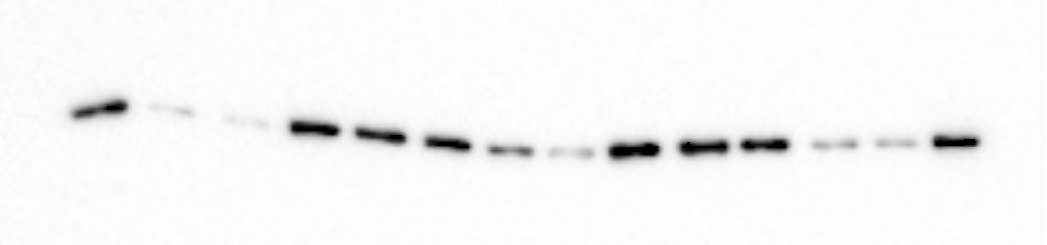

Supplement: Figure 3—figure supplement 1—source data 1. [file elife-92409-fig3-figsupp1-data1.zip › Figure 3-figure supplement 1-source data 1/Uncropped Originals /PanelC - p97 Input blot.tif]

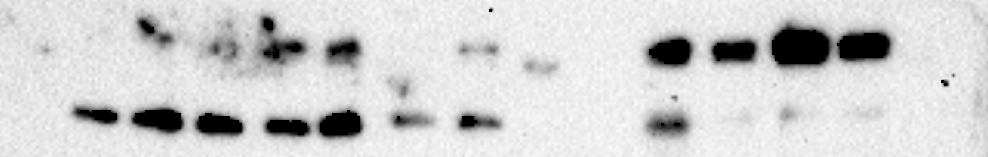

Supplement: Figure 3—figure supplement 1—source data 1. [file elife-92409-fig3-figsupp1-data1.zip › Figure 3-figure supplement 1-source data 1/Uncropped Originals /PanelB - UBXN2B blot.tif]

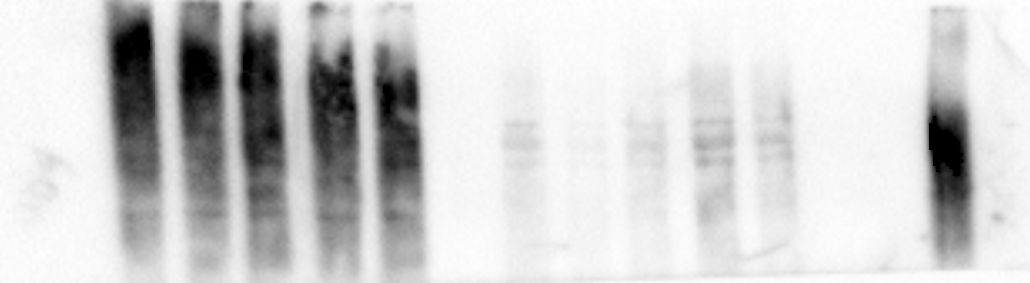

Supplement: Figure 3—figure supplement 1—source data 1. [file elife-92409-fig3-figsupp1-data1.zip › Figure 3-figure supplement 1-source data 1/Uncropped Originals /PanelC - Ubiquitin Input blot.tif]

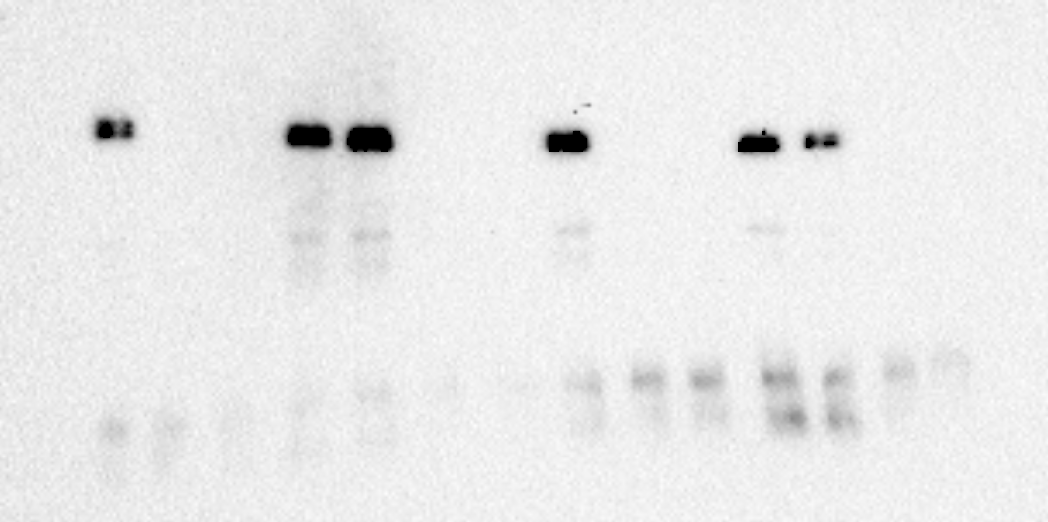

Supplement: Figure 3—figure supplement 1—source data 1. [file elife-92409-fig3-figsupp1-data1.zip › Figure 3-figure supplement 1-source data 1/Uncropped Originals /PanelC - p47 IP blot.tif]

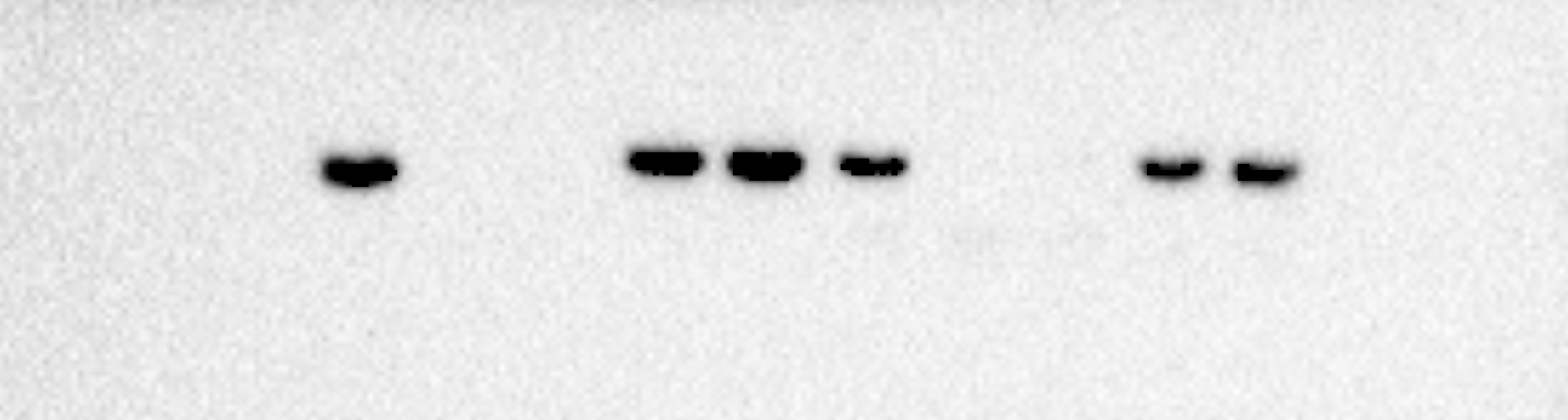

Supplement: Figure 3—figure supplement 1—source data 1. [file elife-92409-fig3-figsupp1-data1.zip › Figure 3-figure supplement 1-source data 1/Uncropped Originals /PanelC - UFD1 IP blot.tif]

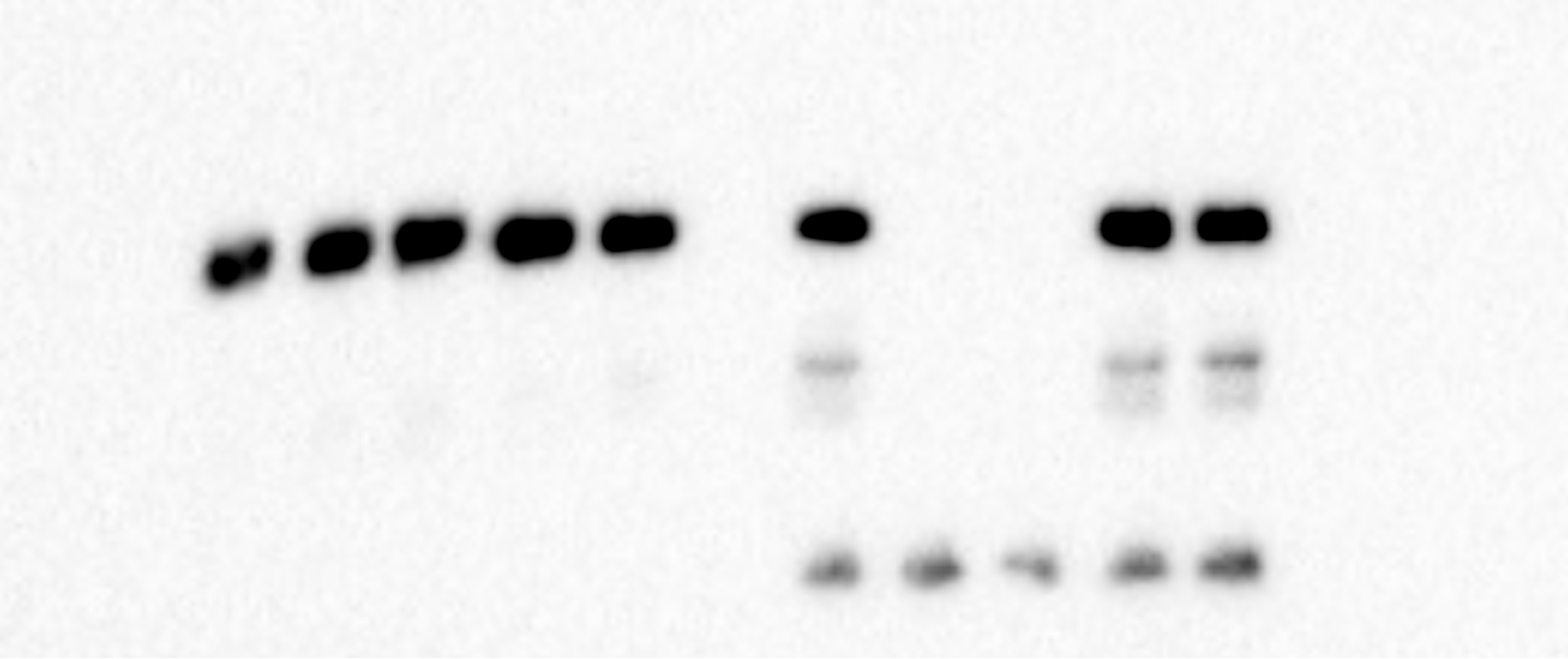

Supplement: Figure 3—figure supplement 1—source data 1. [file elife-92409-fig3-figsupp1-data1.zip › Figure 3-figure supplement 1-source data 1/Uncropped Originals /PanelC - p47 Input blot.tif]

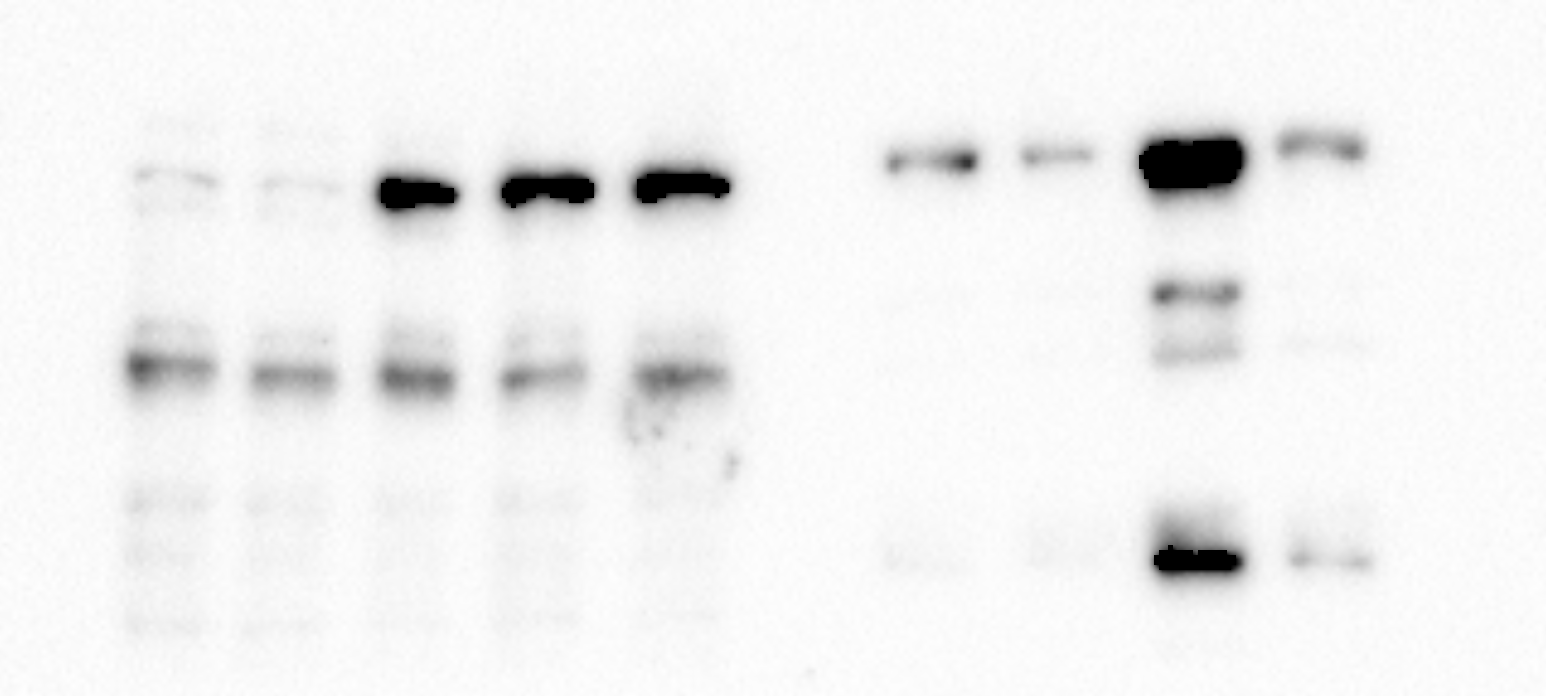

Supplement: Figure 3—figure supplement 1—source data 1. [file elife-92409-fig3-figsupp1-data1.zip › Figure 3-figure supplement 1-source data 1/Uncropped Originals /PanelA - p97 Input and IP blot wm.tif]

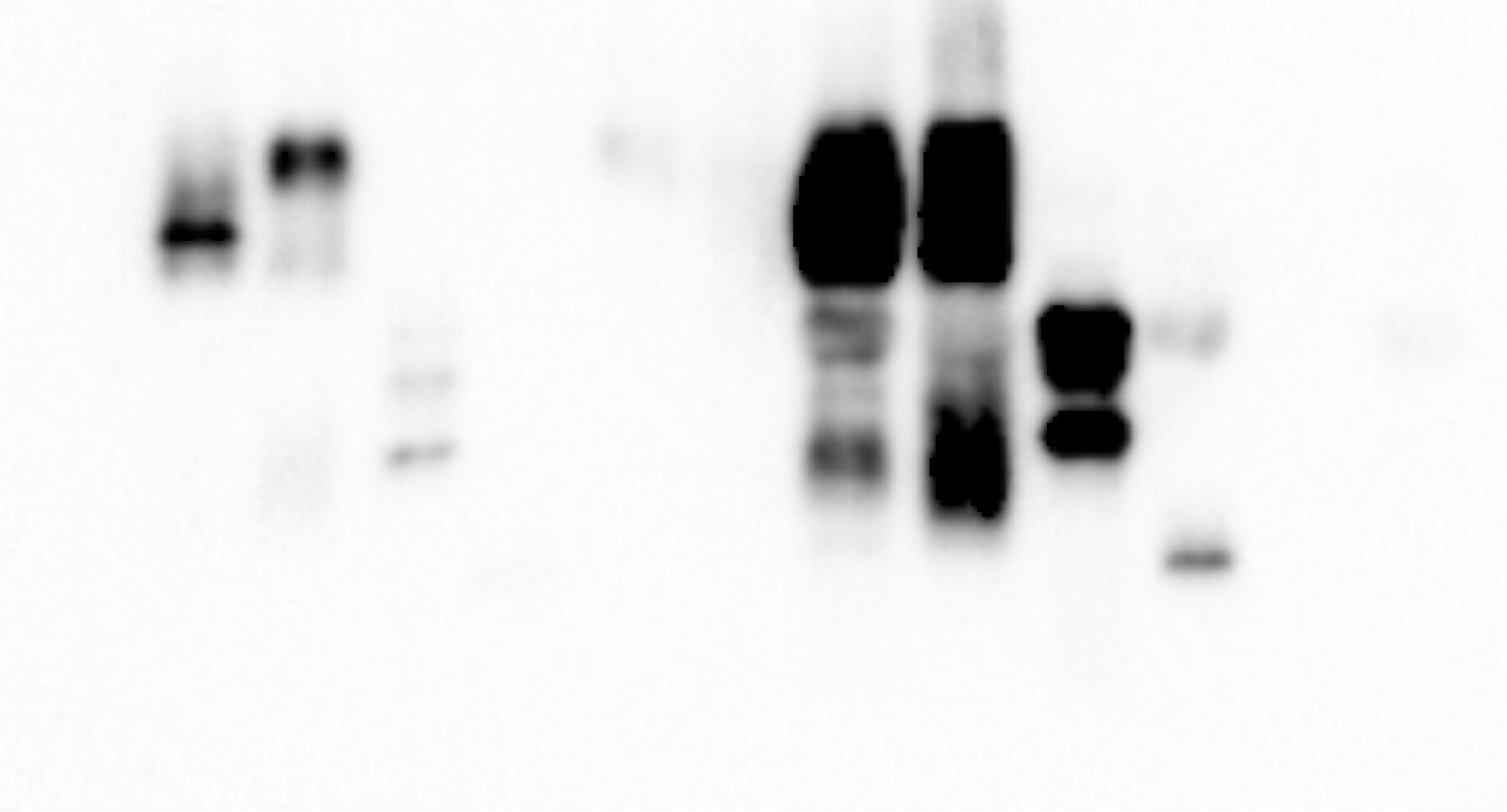

Supplement: Figure 3—figure supplement 1—source data 1. [file elife-92409-fig3-figsupp1-data1.zip › Figure 3-figure supplement 1-source data 1/Uncropped Originals /PanelA - Flag IP blot.tif]

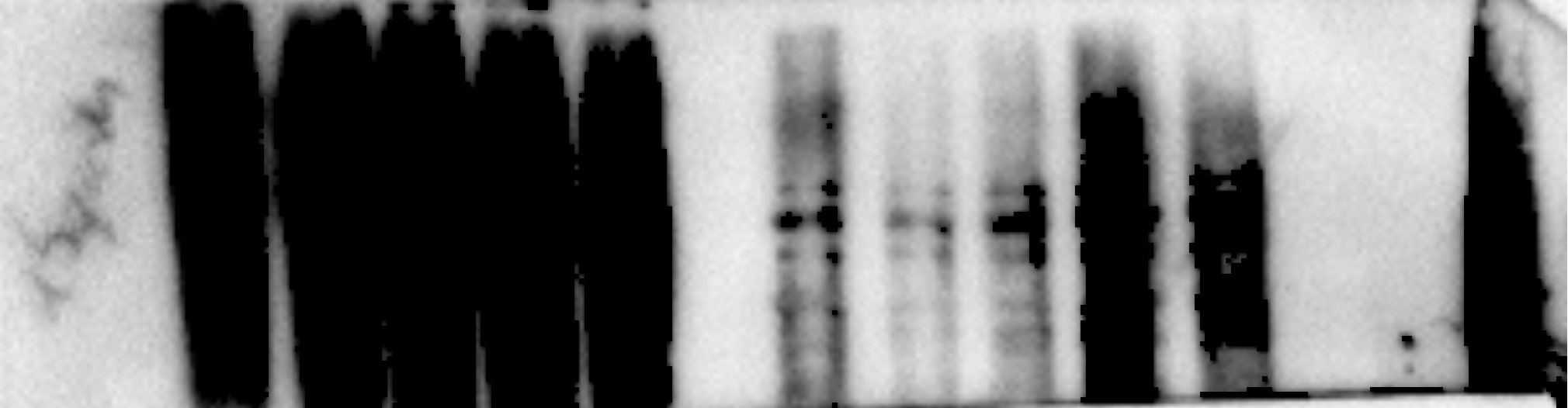

Supplement: Figure 3—figure supplement 1—source data 1. [file elife-92409-fig3-figsupp1-data1.zip › Figure 3-figure supplement 1-source data 1/Uncropped Originals /PanelC - Ubiquitin IP blot.tif]

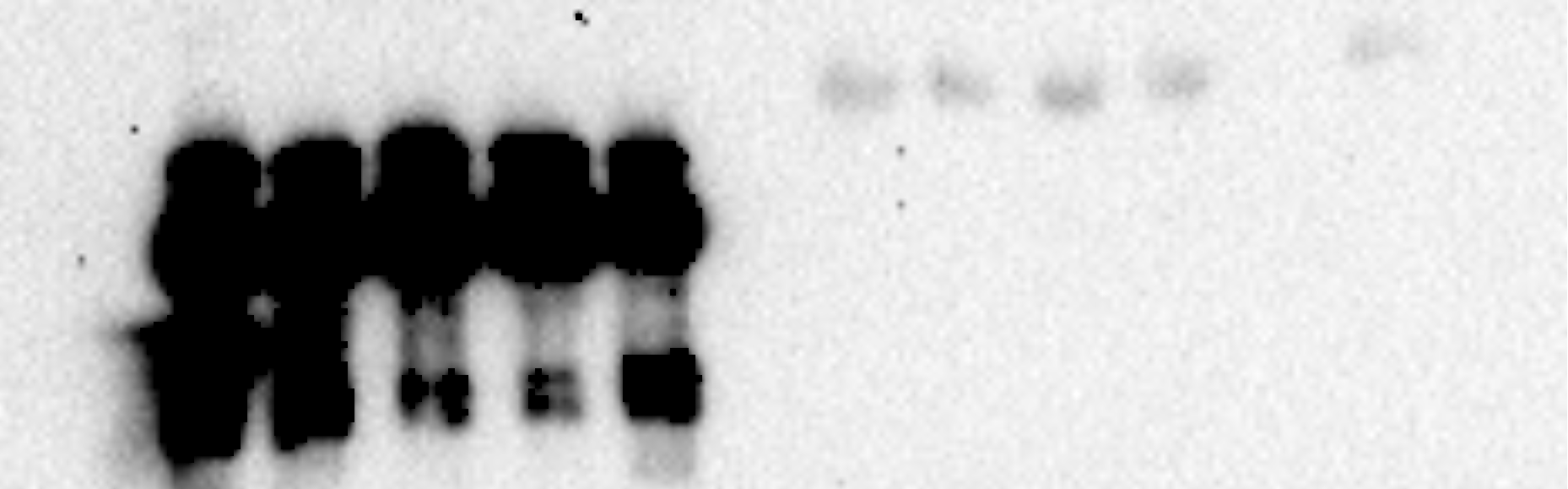

Supplement: Figure 3—figure supplement 1—source data 1. [file elife-92409-fig3-figsupp1-data1.zip › Figure 3-figure supplement 1-source data 1/Uncropped Originals /PanelA - p47 IP blot.tif]

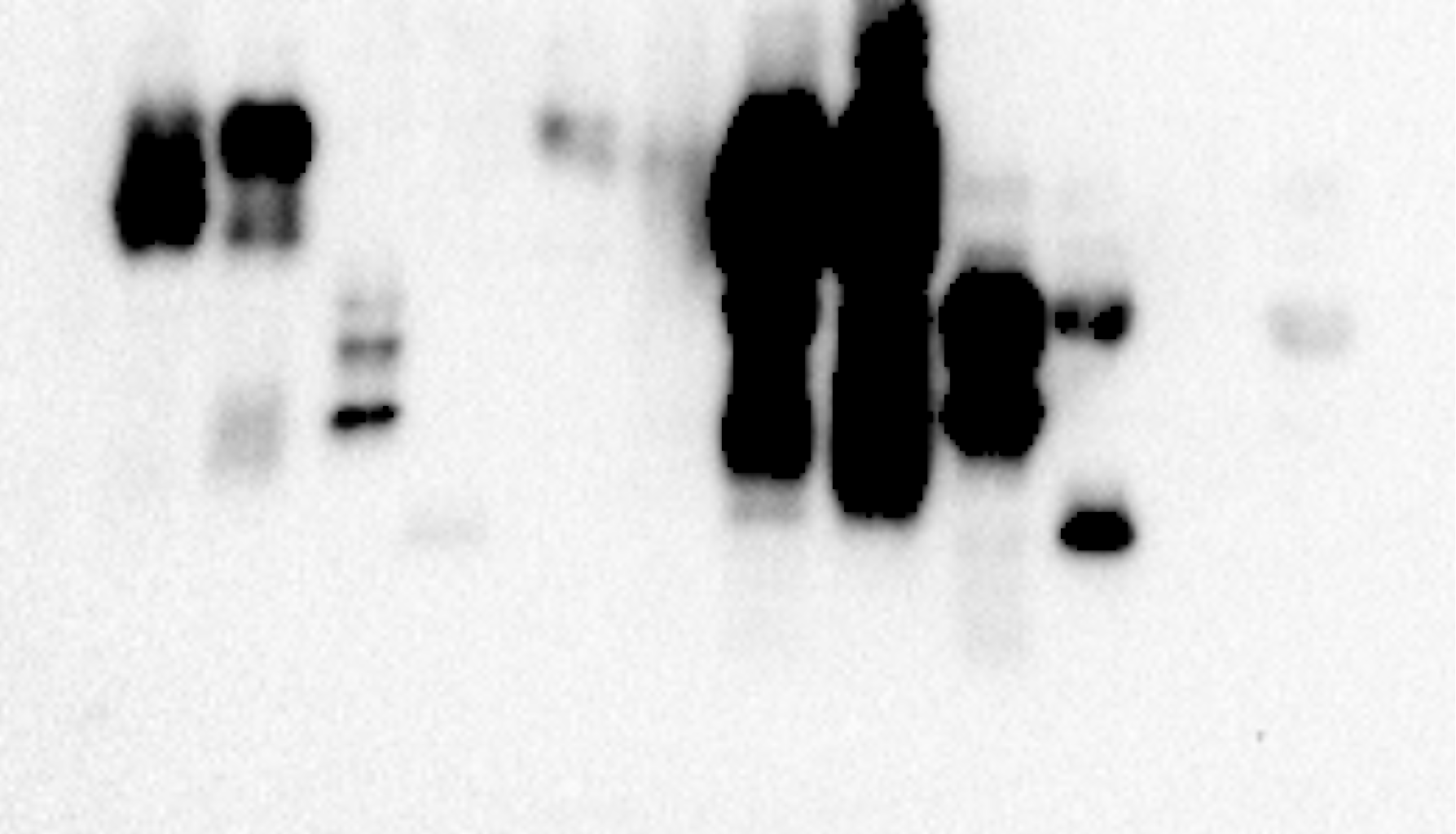

Supplement: Figure 3—figure supplement 1—source data 1. [file elife-92409-fig3-figsupp1-data1.zip › Figure 3-figure supplement 1-source data 1/Uncropped Originals /PanelA - Flag Input blot.tif]

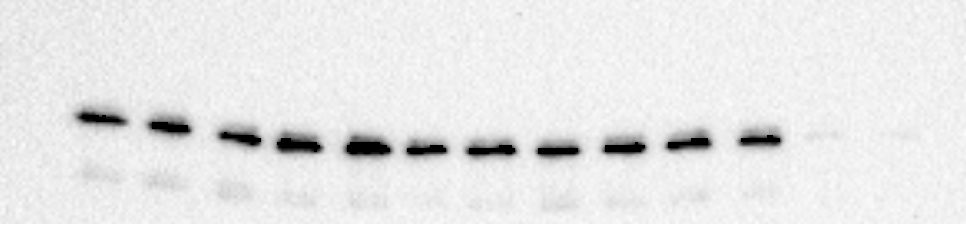

Supplement: Figure 3—figure supplement 1—source data 1. [file elife-92409-fig3-figsupp1-data1.zip › Figure 3-figure supplement 1-source data 1/Uncropped Originals /PanelC - NPL4 Input blot.tif]

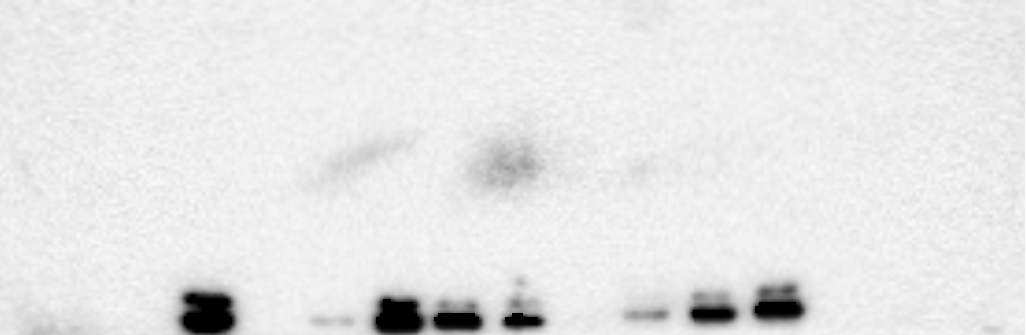

Supplement: Figure 3—figure supplement 1—source data 1. [file elife-92409-fig3-figsupp1-data1.zip › Figure 3-figure supplement 1-source data 1/Uncropped Originals /PanelC - NPL4 IP blot.tif]

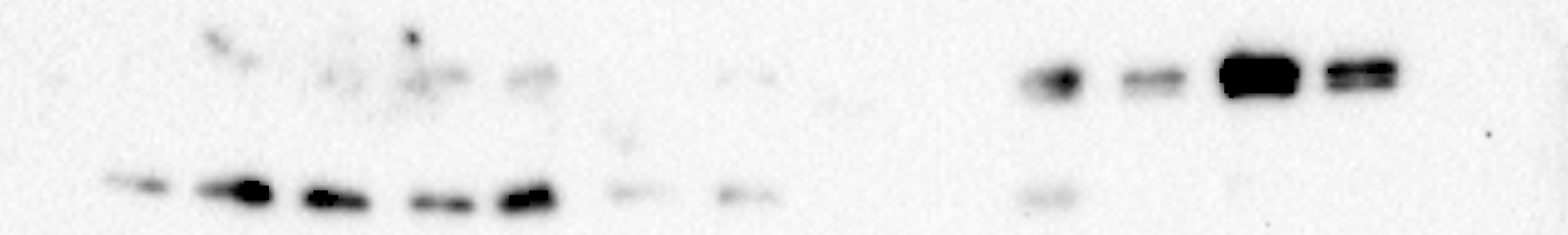

Supplement: Figure 3—figure supplement 1—source data 1. [file elife-92409-fig3-figsupp1-data1.zip › Figure 3-figure supplement 1-source data 1/Uncropped Originals /PanelA - UBXN2B IP blotwm.tif]

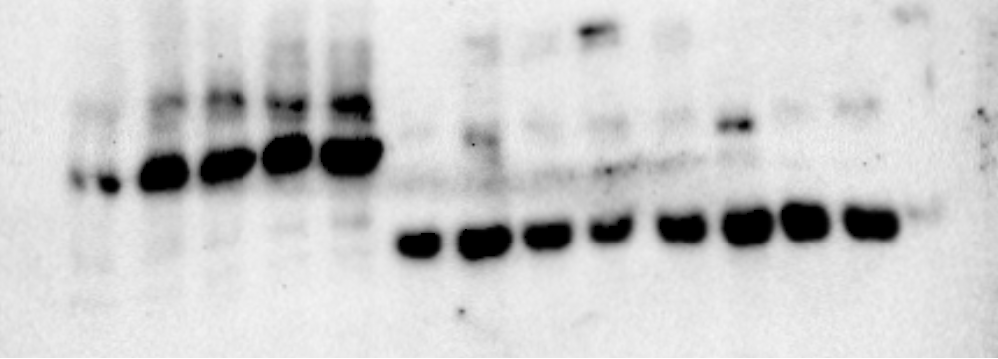

Supplement: Figure 3—figure supplement 1—source data 1. [file elife-92409-fig3-figsupp1-data1.zip › Figure 3-figure supplement 1-source data 1/Uncropped Originals /PanelA - UBXN2B Input blot.tif]

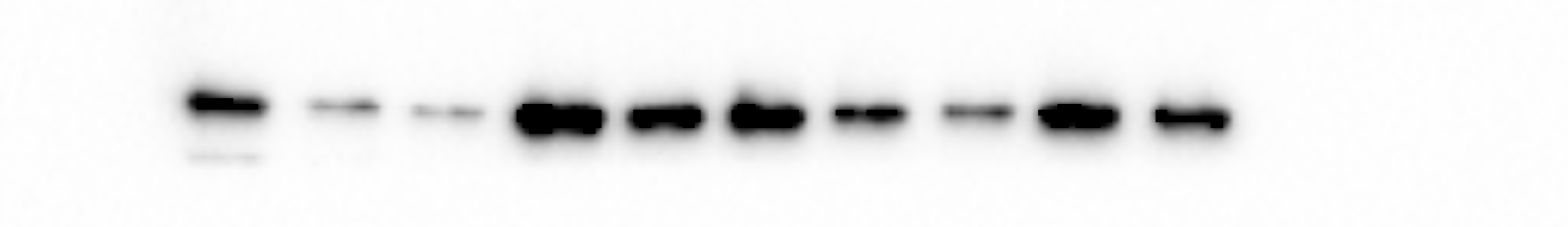

Supplement: Figure 3—figure supplement 1—source data 1. [file elife-92409-fig3-figsupp1-data1.zip › Figure 3-figure supplement 1-source data 1/Uncropped Originals /PanelC - p97 IP blot.tif]

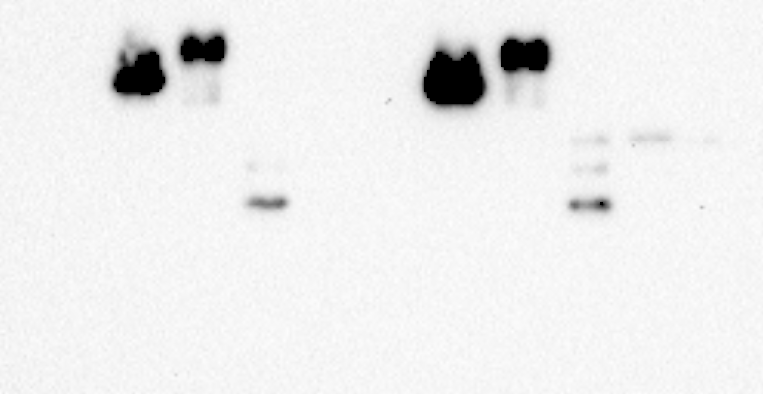

Supplement: Figure 3—figure supplement 1—source data 1. [file elife-92409-fig3-figsupp1-data1.zip › Figure 3-figure supplement 1-source data 1/Uncropped Originals /PanelC - Flag Input blot.tif]

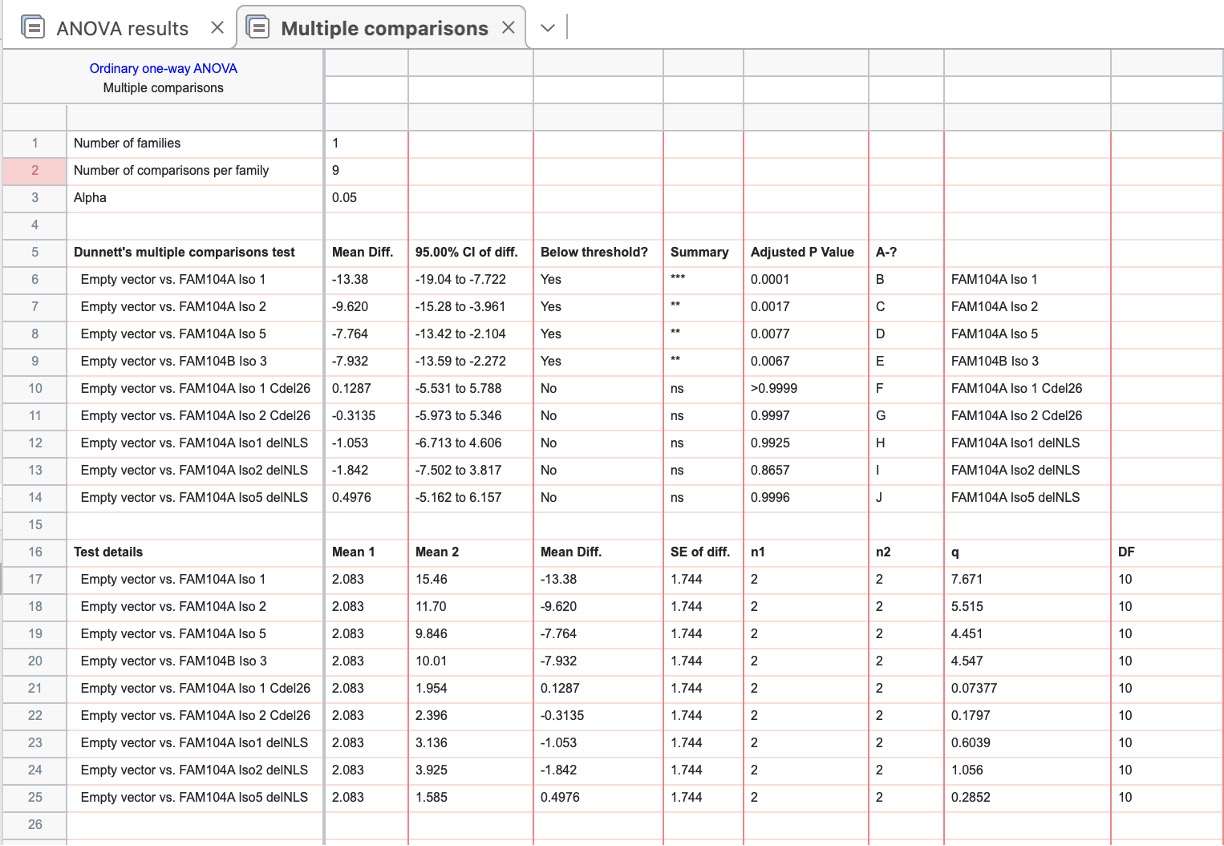

Supplement: Figure 4—source data 1. [file elife-92409-fig4-data1.zip › Figure 4-source data 1/ANOVA Figure 4C.jpg]

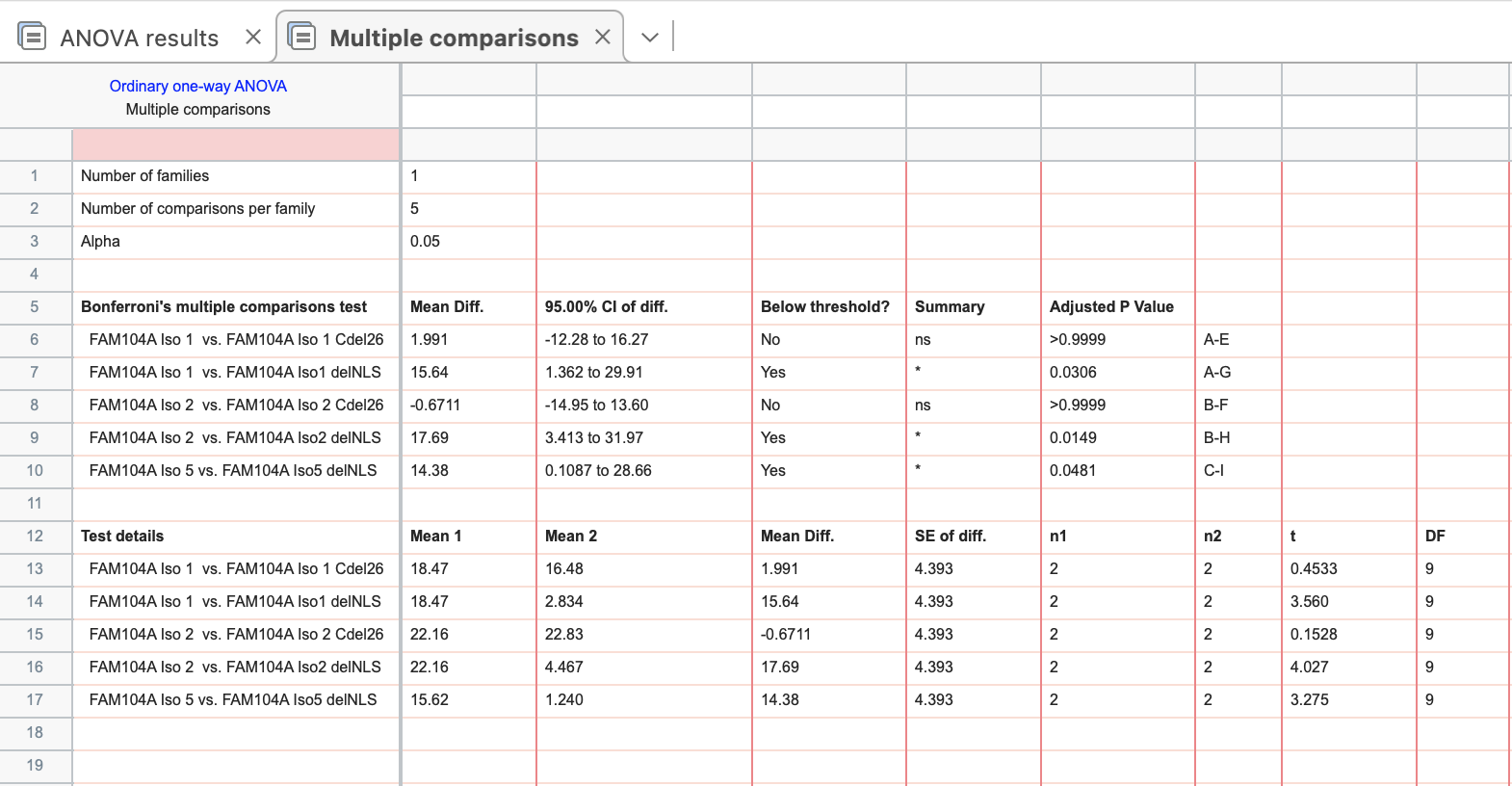

Supplement: Figure 4—source data 1. [file elife-92409-fig4-data1.zip › Figure 4-source data 1/ANOVA Figure 4D.png]

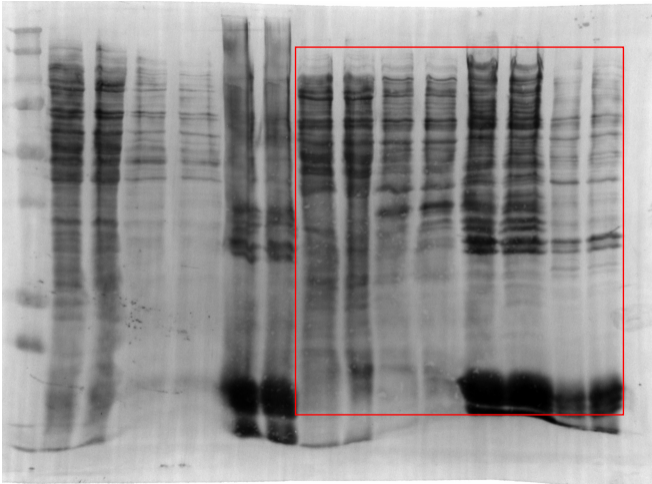

Supplement: Figure 5—source data 1. [file elife-92409-fig5-data1.zip › Figure 5-source data 1/Uncropped Labelled /PanelB - total protein stain.pdf]

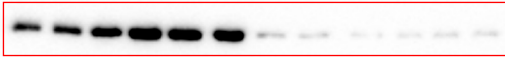

Supplement: Figure 5—source data 1. [file elife-92409-fig5-data1.zip › Figure 5-source data 1/Uncropped Labelled /PanelA - tubulin blot.pdf]

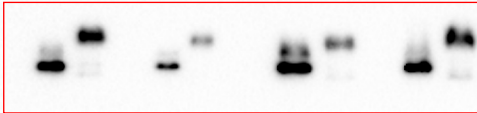

Supplement: Figure 5—source data 1. [file elife-92409-fig5-data1.zip › Figure 5-source data 1/Uncropped Labelled /PanelA - Flag blot.pdf]

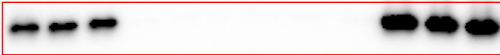

Supplement: Figure 5—source data 1. [file elife-92409-fig5-data1.zip › Figure 5-source data 1/Uncropped Labelled /PanelA - Ub-H2B blot.pdf]

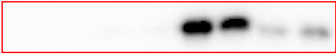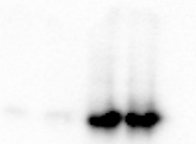

Supplement: Figure 5—source data 1. [file elife-92409-fig5-data1.zip › Figure 5-source data 1/Uncropped Labelled /PanelB - Ub-H2B blot.pdf]

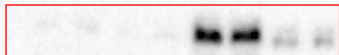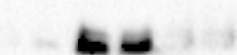

Supplement: Figure 5—source data 1. [file elife-92409-fig5-data1.zip › Figure 5-source data 1/Uncropped Labelled /PanelB - MYC blot.pdf]

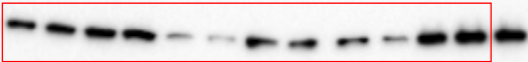

Supplement: Figure 5—source data 1. [file elife-92409-fig5-data1.zip › Figure 5-source data 1/Uncropped Labelled /PanelA - p97 blot.pdf]

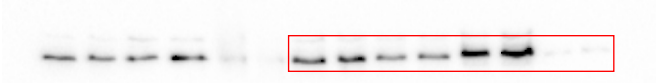

Supplement: Figure 5—source data 1. [file elife-92409-fig5-data1.zip › Figure 5-source data 1/Uncropped Labelled /PanelB - MCM7 blot.pdf]

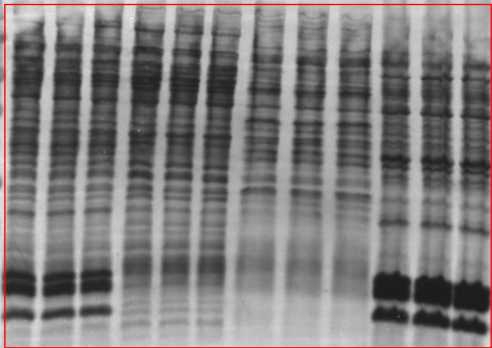

Supplement: Figure 5—source data 1. [file elife-92409-fig5-data1.zip › Figure 5-source data 1/Uncropped Labelled /PanelA - total protein stain.pdf]

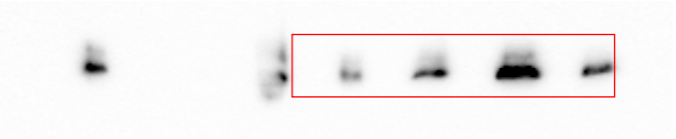

Supplement: Figure 5—source data 1. [file elife-92409-fig5-data1.zip › Figure 5-source data 1/Uncropped Labelled /PanelB - Flag blot.pdf]

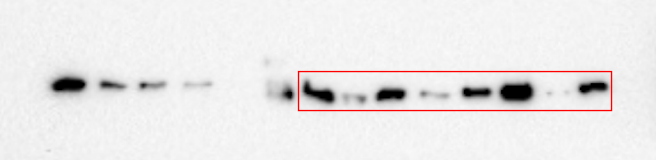

Supplement: Figure 5—source data 1. [file elife-92409-fig5-data1.zip › Figure 5-source data 1/Uncropped Labelled /PanelB - p97 blot.pdf]

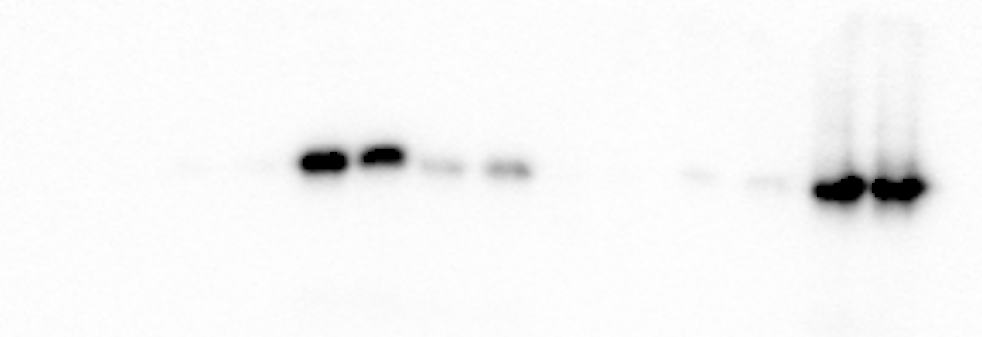

Supplement: Figure 5—source data 1. [file elife-92409-fig5-data1.zip › Figure 5-source data 1/Uncropped Originals /PanelB - Ub-H2B blot.tif]

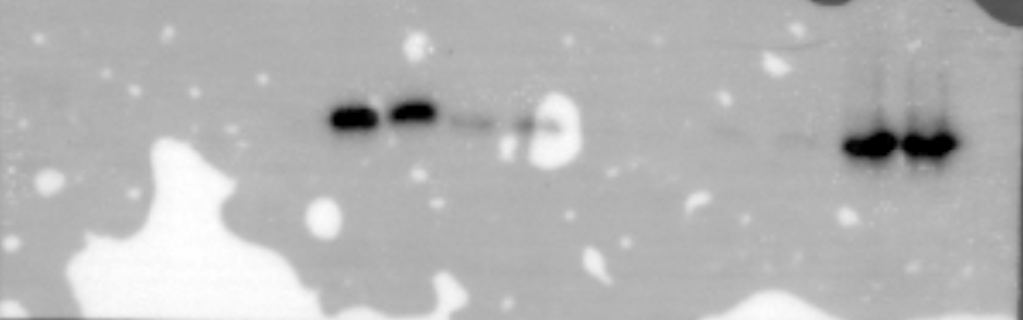

Supplement: Figure 5—source data 1. [file elife-92409-fig5-data1.zip › Figure 5-source data 1/Uncropped Originals /Uncropped merged with marker/PanelB - Ub-H2B blot.tif]

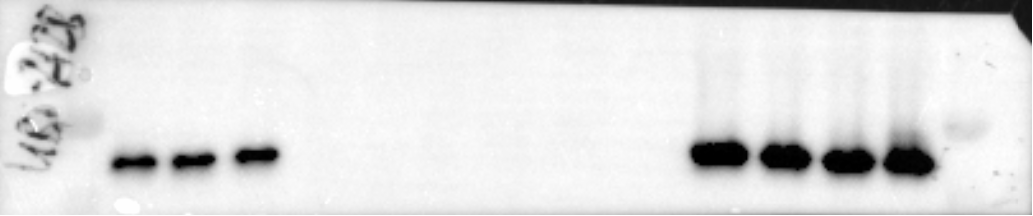

Supplement: Figure 5—source data 1. [file elife-92409-fig5-data1.zip › Figure 5-source data 1/Uncropped Originals /Uncropped merged with marker/PanelA - Ub-H2B blot.tif]

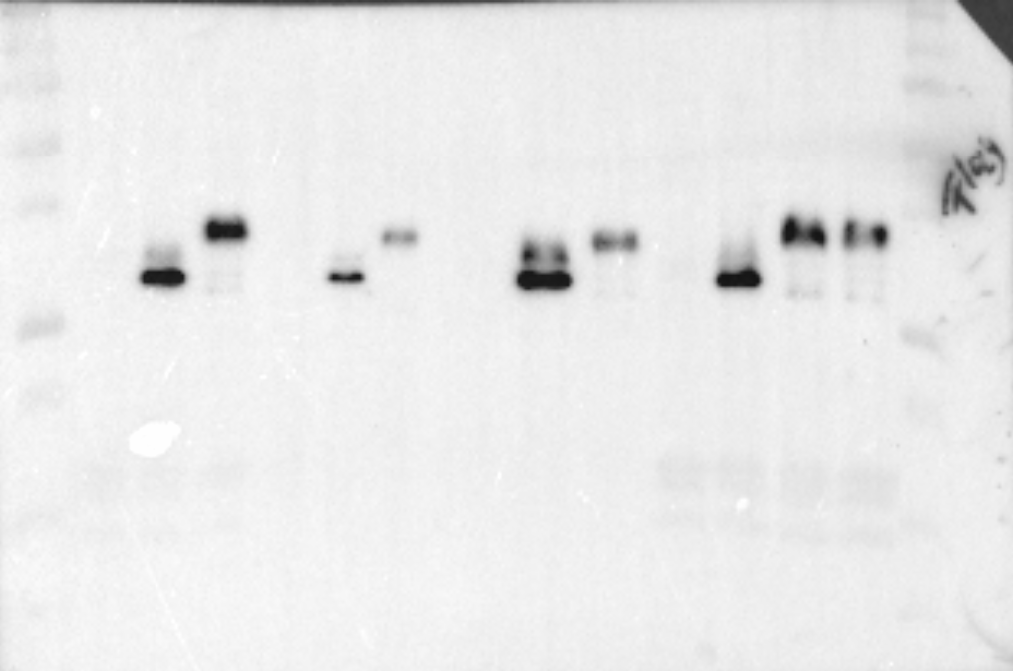

Supplement: Figure 5—source data 1. [file elife-92409-fig5-data1.zip › Figure 5-source data 1/Uncropped Originals /Uncropped merged with marker/PanelA - Flag blot.tif]

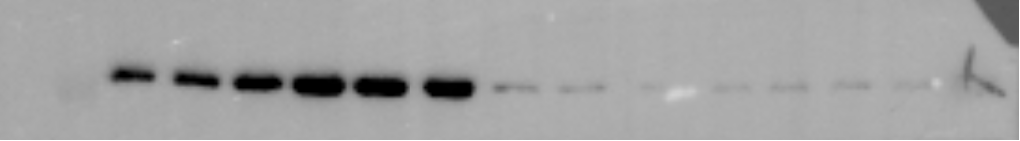

Supplement: Figure 5—source data 1. [file elife-92409-fig5-data1.zip › Figure 5-source data 1/Uncropped Originals /Uncropped merged with marker/PanelA - tubulin blot.tif]

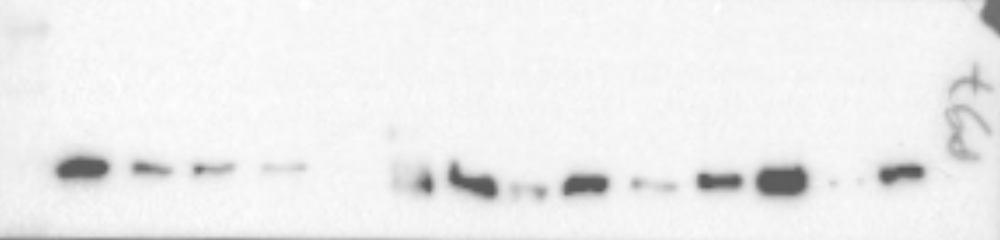

Supplement: Figure 5—source data 1. [file elife-92409-fig5-data1.zip › Figure 5-source data 1/Uncropped Originals /Uncropped merged with marker/PanelB - p97 blot.tif]

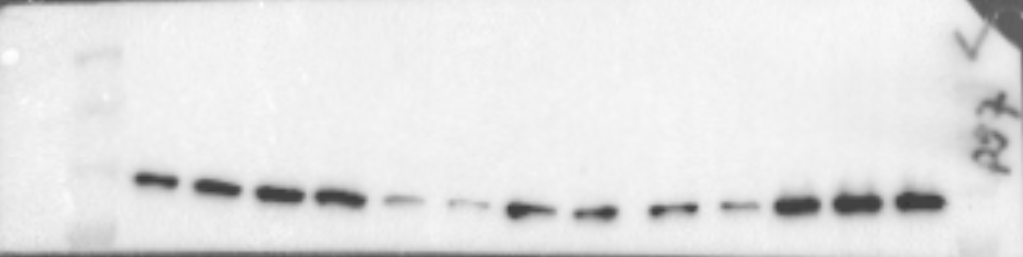

Supplement: Figure 5—source data 1. [file elife-92409-fig5-data1.zip › Figure 5-source data 1/Uncropped Originals /Uncropped merged with marker/PanelA - p97 blot.tif]

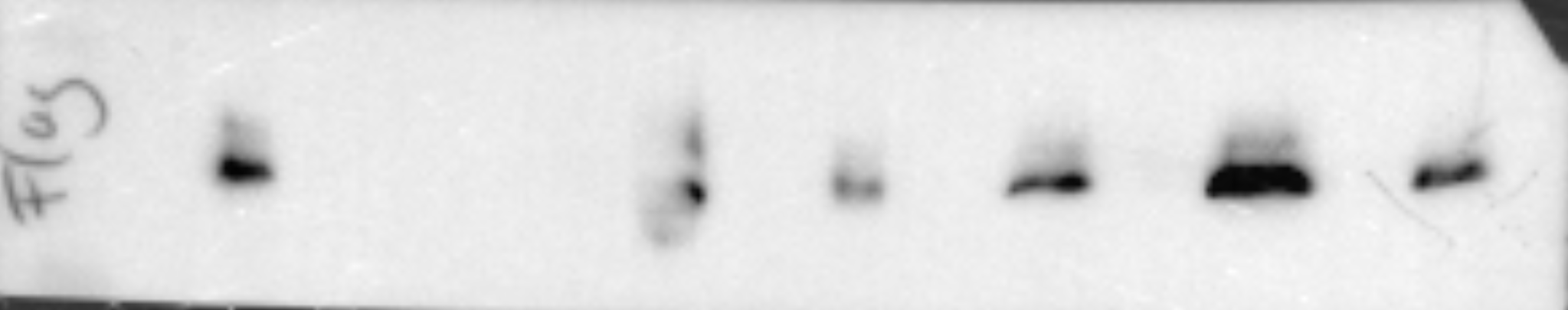

Supplement: Figure 5—source data 1. [file elife-92409-fig5-data1.zip › Figure 5-source data 1/Uncropped Originals /Uncropped merged with marker/PanelB - Flag blot.tif]

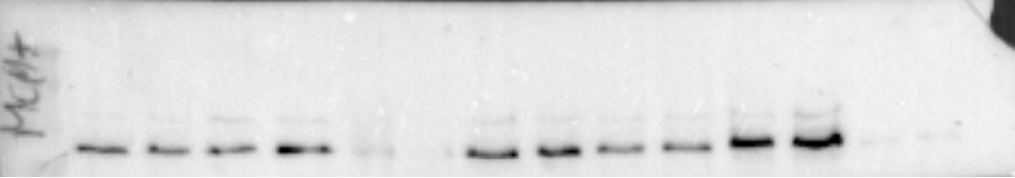

Supplement: Figure 5—source data 1. [file elife-92409-fig5-data1.zip › Figure 5-source data 1/Uncropped Originals /Uncropped merged with marker/PanelB - MCM7 blot.tif]

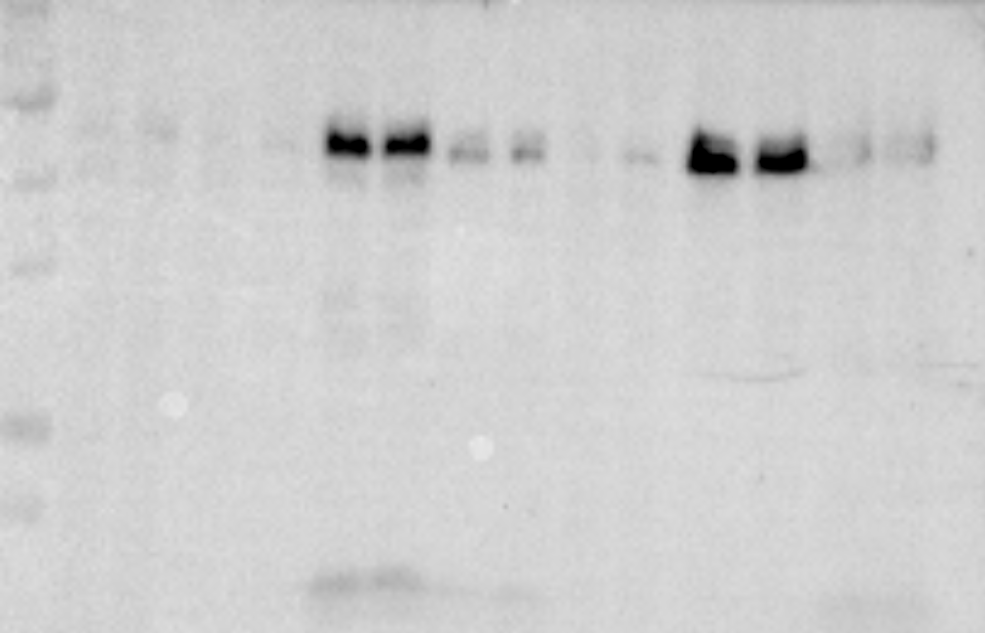

Supplement: Figure 5—source data 1. [file elife-92409-fig5-data1.zip › Figure 5-source data 1/Uncropped Originals /Uncropped merged with marker/PanelB - MYC blot.tif]

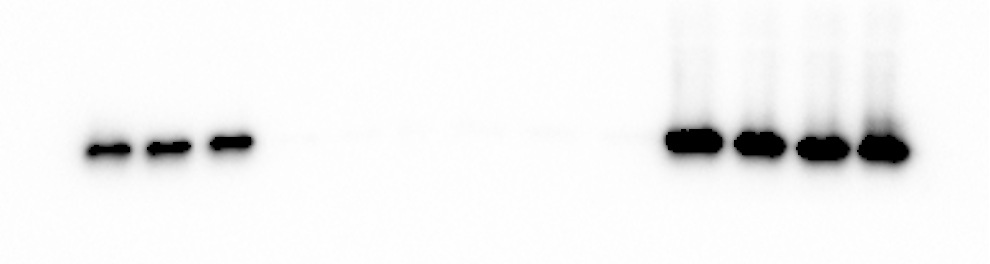

Supplement: Figure 5—source data 1. [file elife-92409-fig5-data1.zip › Figure 5-source data 1/Uncropped Originals /PanelA - Ub-H2B blot.tif]

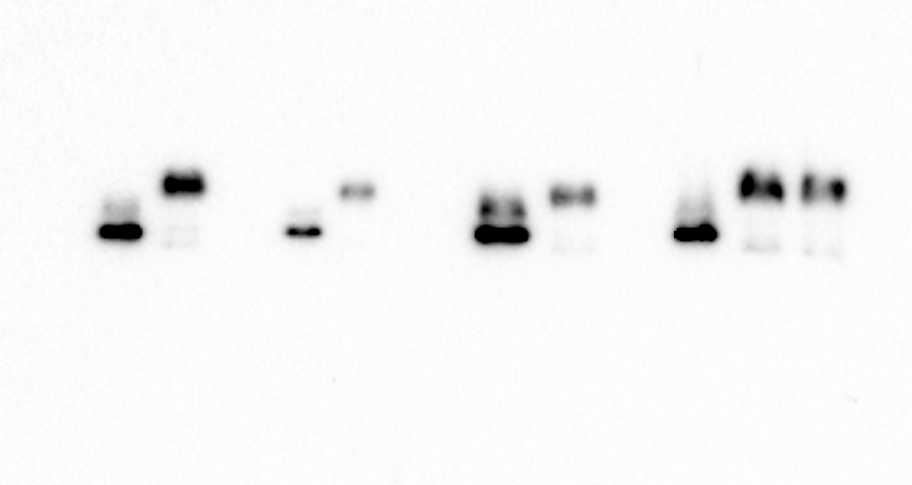

Supplement: Figure 5—source data 1. [file elife-92409-fig5-data1.zip › Figure 5-source data 1/Uncropped Originals /PanelA - Flag blot.tif]

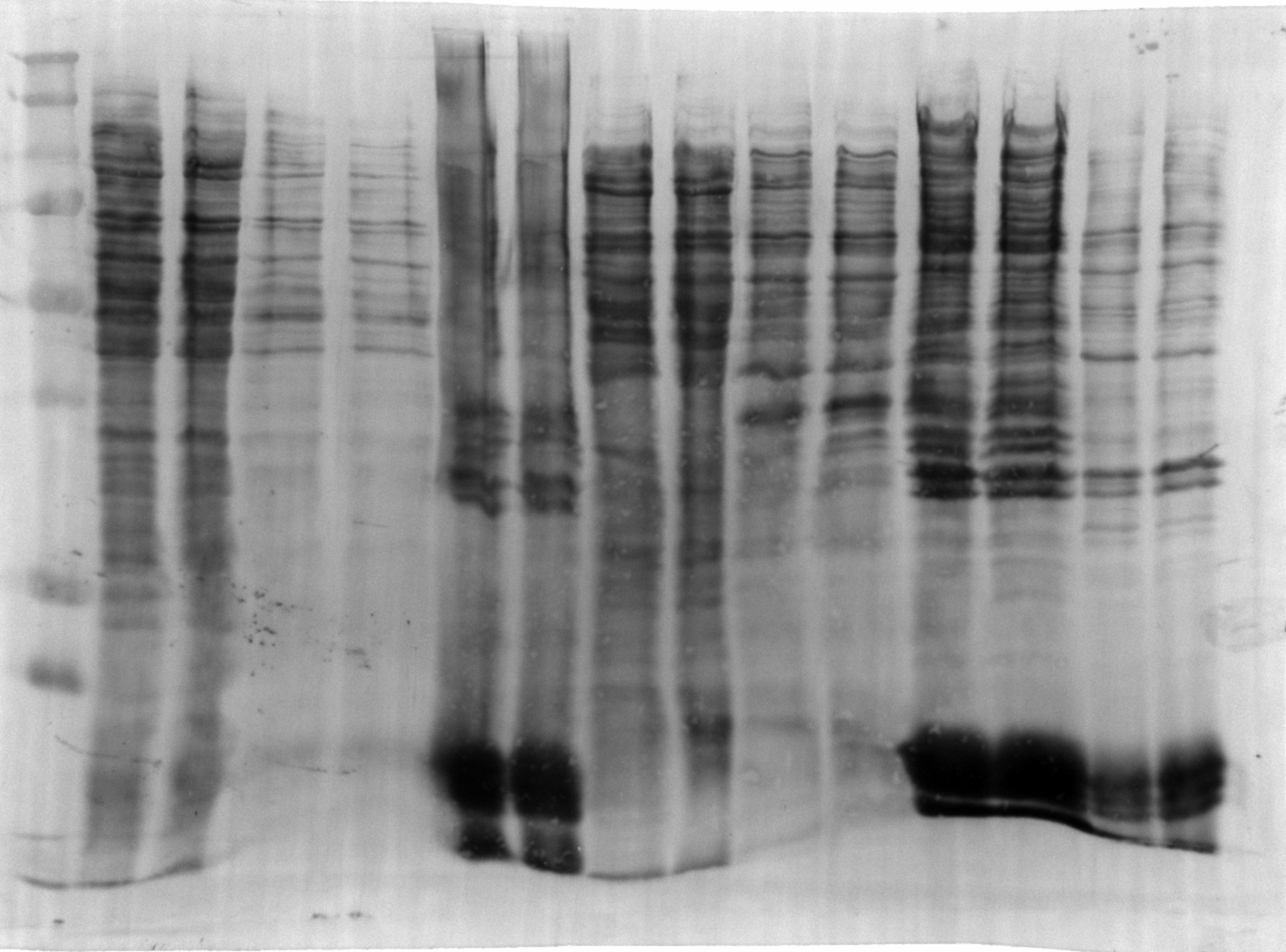

Supplement: Figure 5—source data 1. [file elife-92409-fig5-data1.zip › Figure 5-source data 1/Uncropped Originals /PanelB - total protein stain.tif]

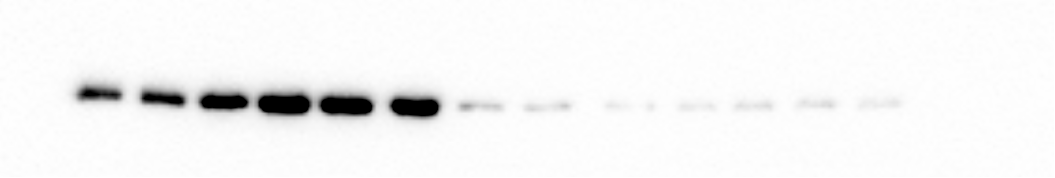

Supplement: Figure 5—source data 1. [file elife-92409-fig5-data1.zip › Figure 5-source data 1/Uncropped Originals /PanelA - tubulin blot.tif]

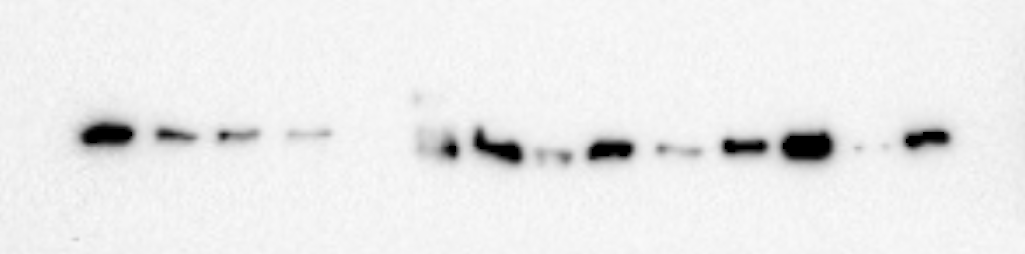

Supplement: Figure 5—source data 1. [file elife-92409-fig5-data1.zip › Figure 5-source data 1/Uncropped Originals /PanelB - p97 blot.tif]

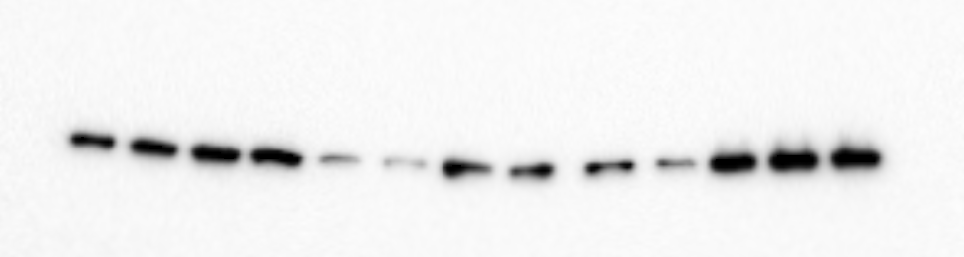

Supplement: Figure 5—source data 1. [file elife-92409-fig5-data1.zip › Figure 5-source data 1/Uncropped Originals /PanelA - p97 blot.tif]

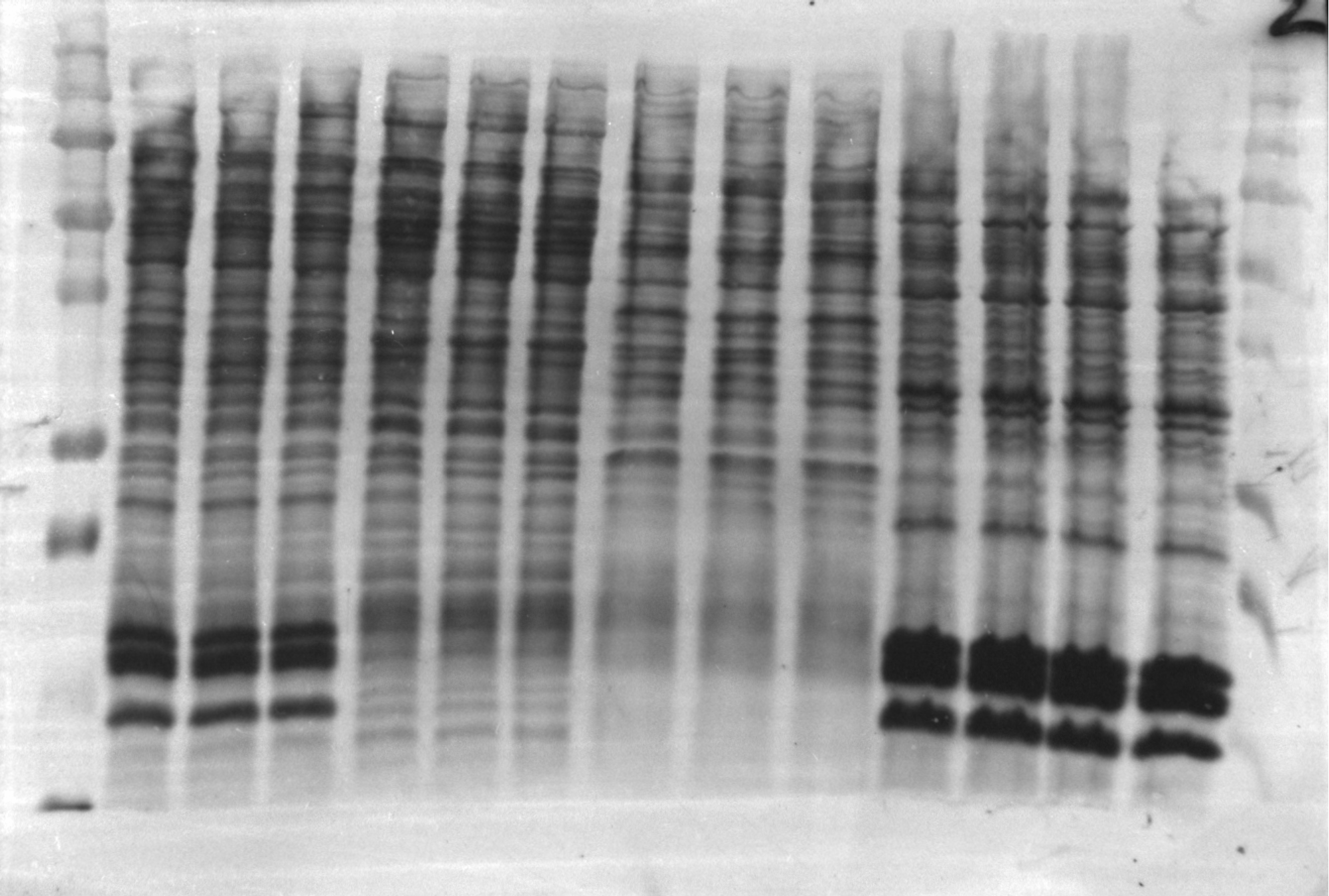

Supplement: Figure 5—source data 1. [file elife-92409-fig5-data1.zip › Figure 5-source data 1/Uncropped Originals /PanelA - total protein stain.tif]

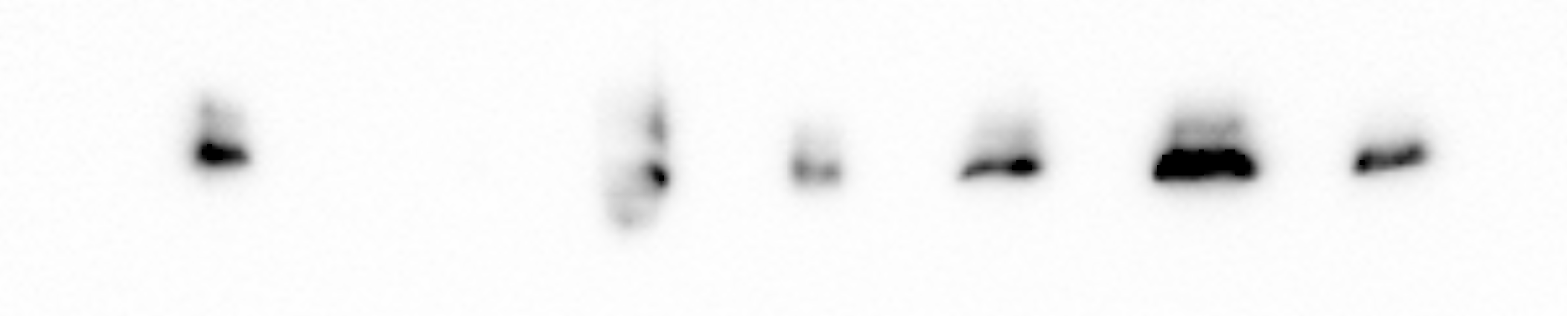

Supplement: Figure 5—source data 1. [file elife-92409-fig5-data1.zip › Figure 5-source data 1/Uncropped Originals /PanelB - Flag blot.tif]

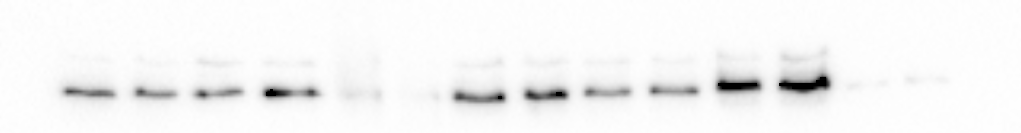

Supplement: Figure 5—source data 1. [file elife-92409-fig5-data1.zip › Figure 5-source data 1/Uncropped Originals /PanelB - MCM7 blot.tif]

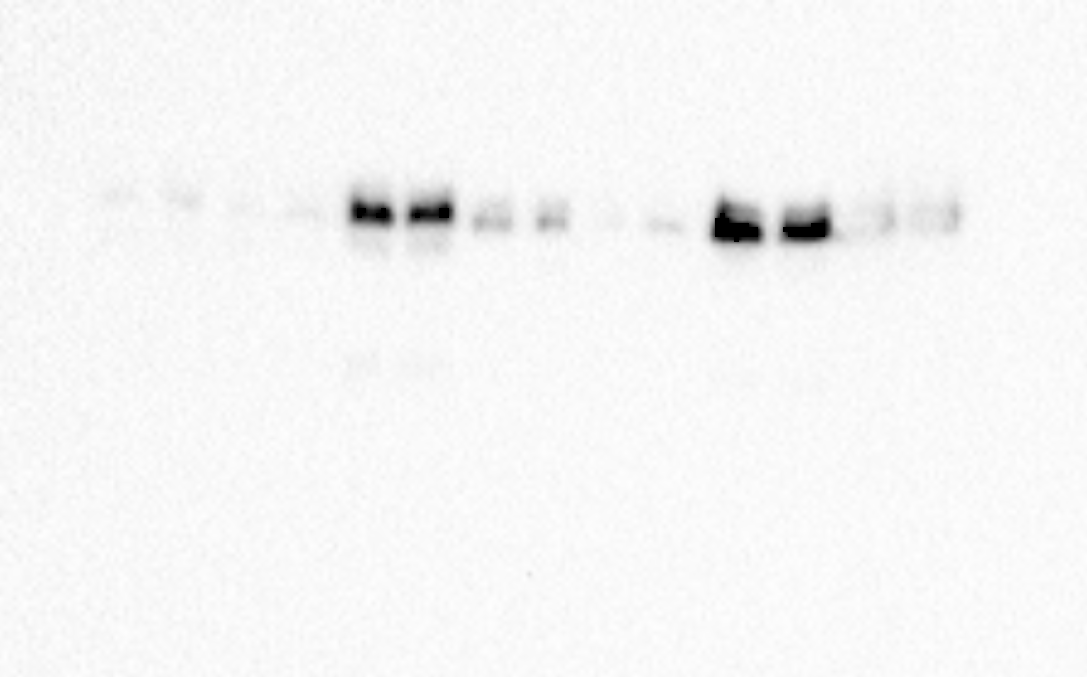

Supplement: Figure 5—source data 1. [file elife-92409-fig5-data1.zip › Figure 5-source data 1/Uncropped Originals /PanelB - MYC blot.tif]

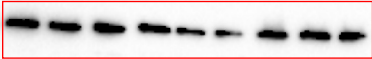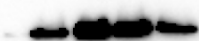

Supplement: Figure 5—figure supplement 1—source data 1. [file elife-92409-fig5-figsupp1-data1.zip › Figure 5-figure supplement 1-source data 1/Uncropped Labelled/PanelA - p97.pdf]

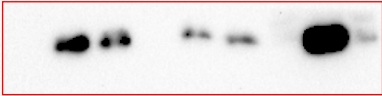

Supplement: Figure 5—figure supplement 1—source data 1. [file elife-92409-fig5-figsupp1-data1.zip › Figure 5-figure supplement 1-source data 1/Uncropped Labelled/PanelA - Flag.pdf]

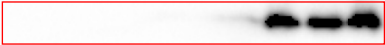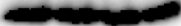

Supplement: Figure 5—figure supplement 1—source data 1. [file elife-92409-fig5-figsupp1-data1.zip › Figure 5-figure supplement 1-source data 1/Uncropped Labelled/PanelB - Ubi-H2B.pdf]

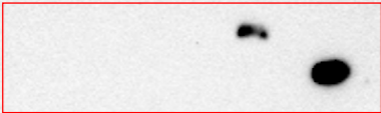

Supplement: Figure 5—figure supplement 1—source data 1. [file elife-92409-fig5-figsupp1-data1.zip › Figure 5-figure supplement 1-source data 1/Uncropped Labelled/PanelB - Flag.pdf]

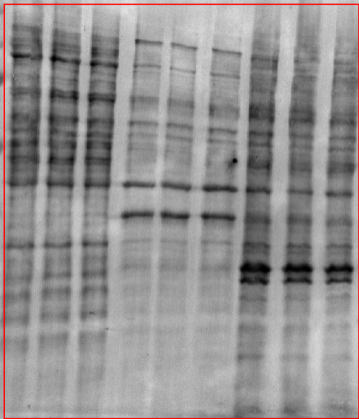

Supplement: Figure 5—figure supplement 1—source data 1. [file elife-92409-fig5-figsupp1-data1.zip › Figure 5-figure supplement 1-source data 1/Uncropped Labelled/PanelB- total protein stain.pdf]

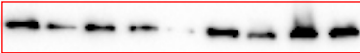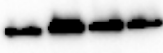

Supplement: Figure 5—figure supplement 1—source data 1. [file elife-92409-fig5-figsupp1-data1.zip › Figure 5-figure supplement 1-source data 1/Uncropped Labelled/PanelB - p97.pdf]

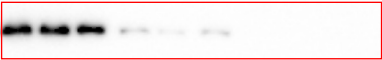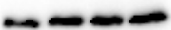

Supplement: Figure 5—figure supplement 1—source data 1. [file elife-92409-fig5-figsupp1-data1.zip › Figure 5-figure supplement 1-source data 1/Uncropped Labelled/PanelA - tubulin.pdf]

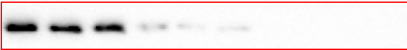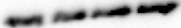

Supplement: Figure 5—figure supplement 1—source data 1. [file elife-92409-fig5-figsupp1-data1.zip › Figure 5-figure supplement 1-source data 1/Uncropped Labelled/PanelB - tubulin.pdf]

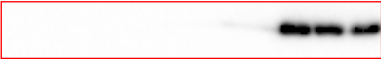

Supplement: Figure 5—figure supplement 1—source data 1. [file elife-92409-fig5-figsupp1-data1.zip › Figure 5-figure supplement 1-source data 1/Uncropped Labelled/PanelA - Ubi-H2B.pdf]

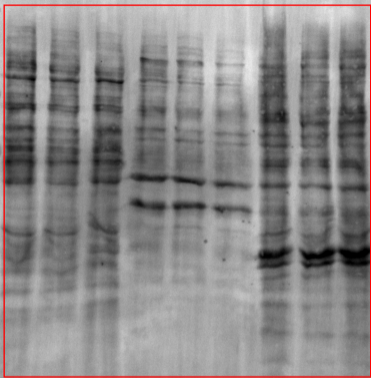

Supplement: Figure 5—figure supplement 1—source data 1. [file elife-92409-fig5-figsupp1-data1.zip › Figure 5-figure supplement 1-source data 1/Uncropped Labelled/PanelA - total protein stain.pdf]

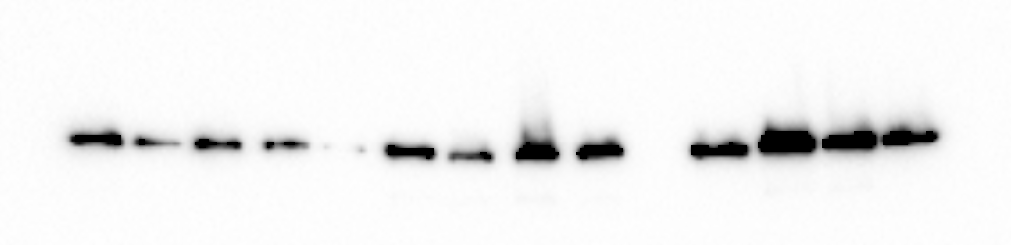

Supplement: Figure 5—figure supplement 1—source data 1. [file elife-92409-fig5-figsupp1-data1.zip › Figure 5-figure supplement 1-source data 1/Uncropped Originals /PanelB - p97 blot .tif]

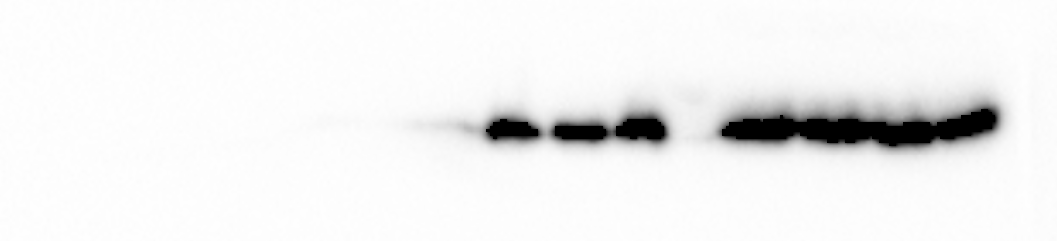

Supplement: Figure 5—figure supplement 1—source data 1. [file elife-92409-fig5-figsupp1-data1.zip › Figure 5-figure supplement 1-source data 1/Uncropped Originals /PanelB - Ub-H2B blot.tif]

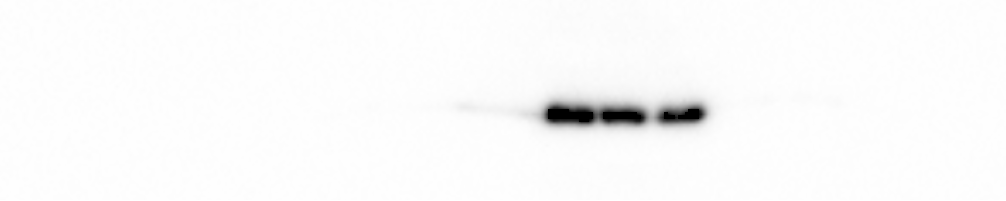

Supplement: Figure 5—figure supplement 1—source data 1. [file elife-92409-fig5-figsupp1-data1.zip › Figure 5-figure supplement 1-source data 1/Uncropped Originals /PanelA - Ub-H2B blot.tif]

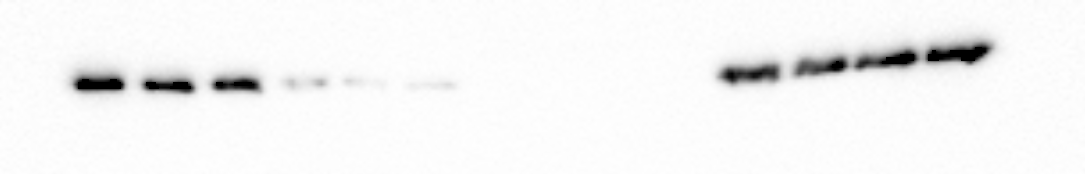

Supplement: Figure 5—figure supplement 1—source data 1. [file elife-92409-fig5-figsupp1-data1.zip › Figure 5-figure supplement 1-source data 1/Uncropped Originals /PanelB - tubulin blot.tif]

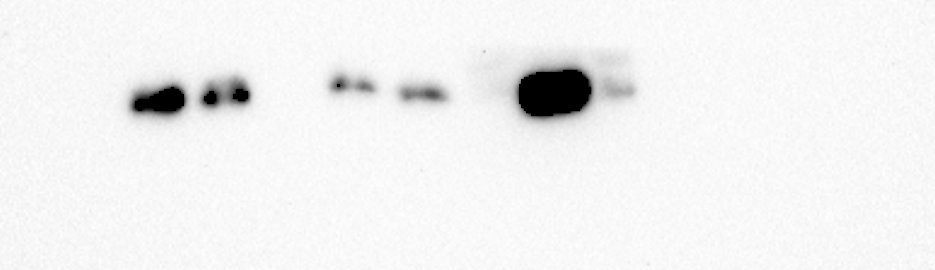

Supplement: Figure 5—figure supplement 1—source data 1. [file elife-92409-fig5-figsupp1-data1.zip › Figure 5-figure supplement 1-source data 1/Uncropped Originals /PanelA - Flag blot.tif]

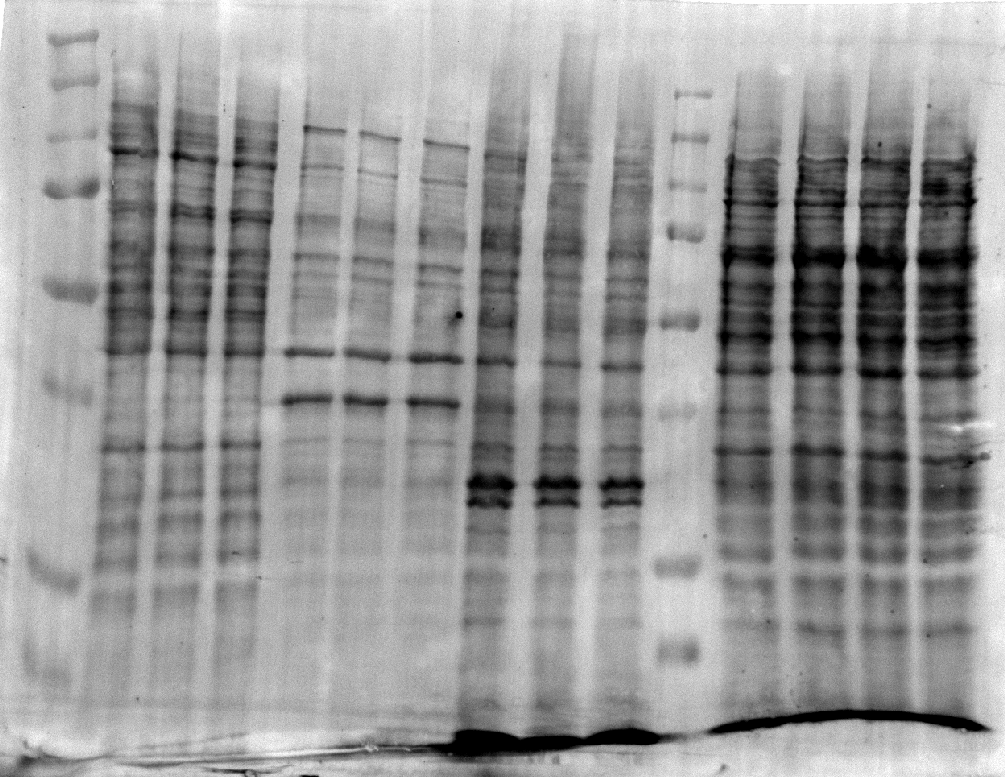

Supplement: Figure 5—figure supplement 1—source data 1. [file elife-92409-fig5-figsupp1-data1.zip › Figure 5-figure supplement 1-source data 1/Uncropped Originals /PanelB - total protein stain.tif]

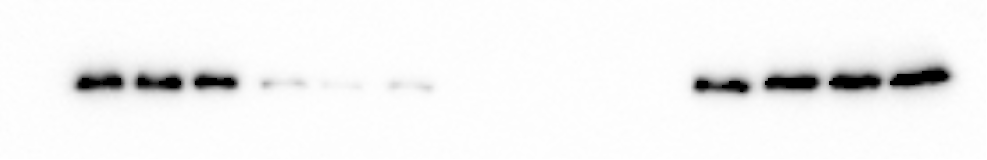

Supplement: Figure 5—figure supplement 1—source data 1. [file elife-92409-fig5-figsupp1-data1.zip › Figure 5-figure supplement 1-source data 1/Uncropped Originals /PanelA - tubulin blot.tif]

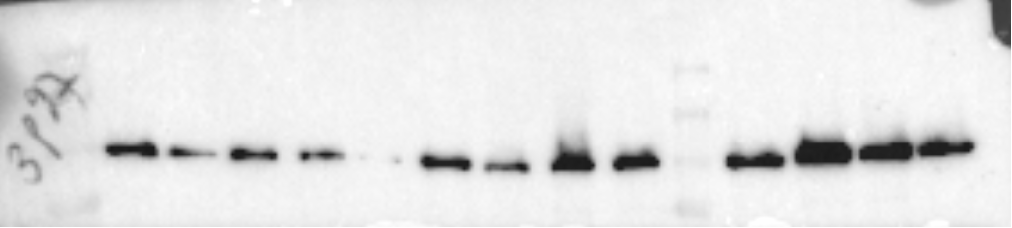

Supplement: Figure 5—figure supplement 1—source data 1. [file elife-92409-fig5-figsupp1-data1.zip › Figure 5-figure supplement 1-source data 1/Uncropped Originals /Originals merged with marker/PanelB - p97 blot .tif]

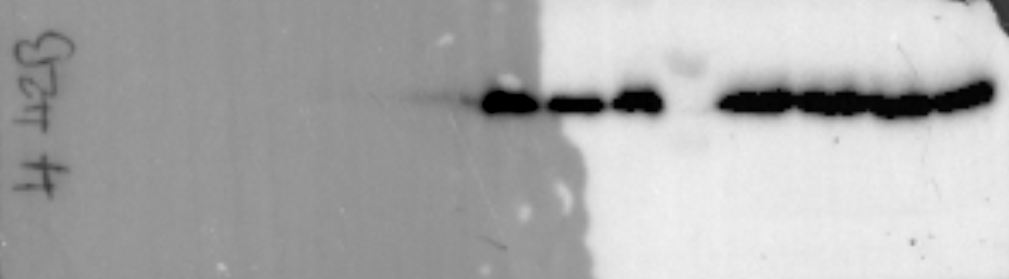

Supplement: Figure 5—figure supplement 1—source data 1. [file elife-92409-fig5-figsupp1-data1.zip › Figure 5-figure supplement 1-source data 1/Uncropped Originals /Originals merged with marker/PanelB - Ub-H2B blot.tif]

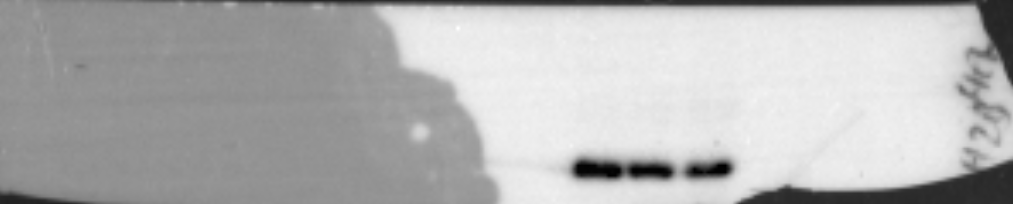

Supplement: Figure 5—figure supplement 1—source data 1. [file elife-92409-fig5-figsupp1-data1.zip › Figure 5-figure supplement 1-source data 1/Uncropped Originals /Originals merged with marker/PanelA - Ub-H2B blot.tif]

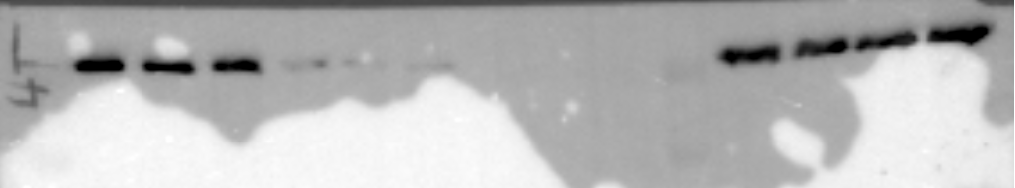

Supplement: Figure 5—figure supplement 1—source data 1. [file elife-92409-fig5-figsupp1-data1.zip › Figure 5-figure supplement 1-source data 1/Uncropped Originals /Originals merged with marker/PanelB - tubulin blot.tif]

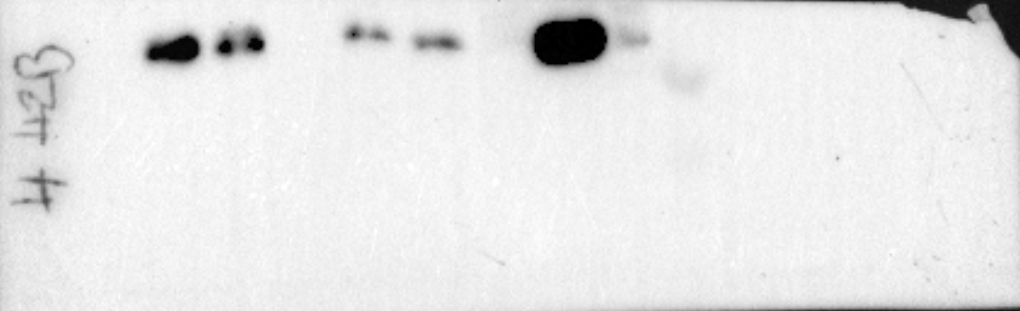

Supplement: Figure 5—figure supplement 1—source data 1. [file elife-92409-fig5-figsupp1-data1.zip › Figure 5-figure supplement 1-source data 1/Uncropped Originals /Originals merged with marker/PanelA - Flag blot.tif]

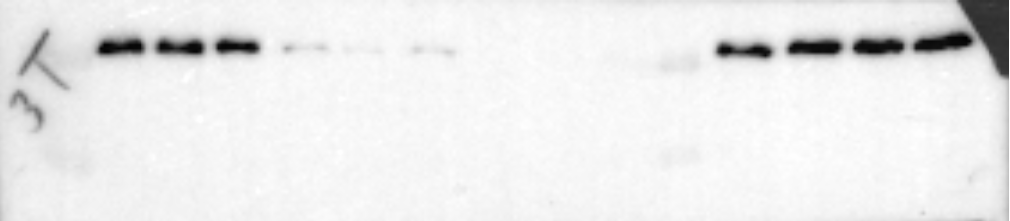

Supplement: Figure 5—figure supplement 1—source data 1. [file elife-92409-fig5-figsupp1-data1.zip › Figure 5-figure supplement 1-source data 1/Uncropped Originals /Originals merged with marker/PanelA - tubulin blot.tif]

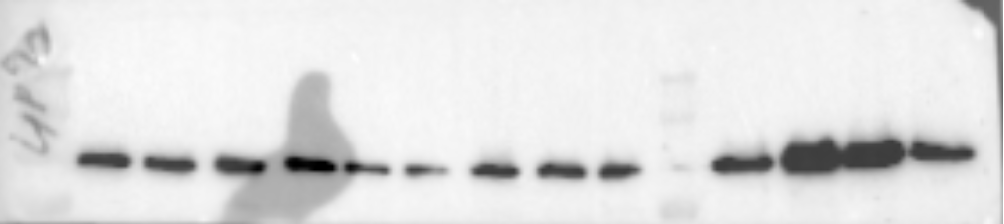

Supplement: Figure 5—figure supplement 1—source data 1. [file elife-92409-fig5-figsupp1-data1.zip › Figure 5-figure supplement 1-source data 1/Uncropped Originals /Originals merged with marker/PanelA - p97 blot .tif]

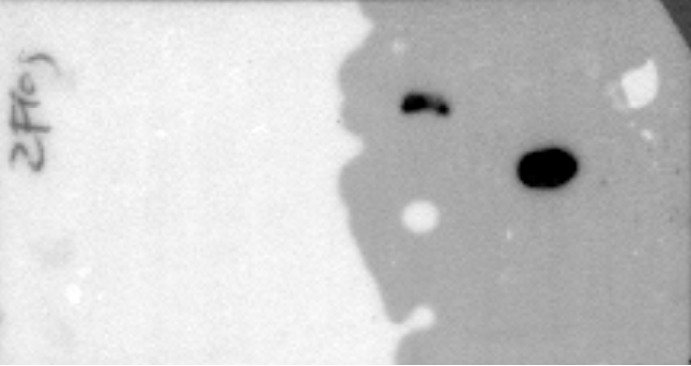

Supplement: Figure 5—figure supplement 1—source data 1. [file elife-92409-fig5-figsupp1-data1.zip › Figure 5-figure supplement 1-source data 1/Uncropped Originals /Originals merged with marker/PanelB - Flag blot.tif]

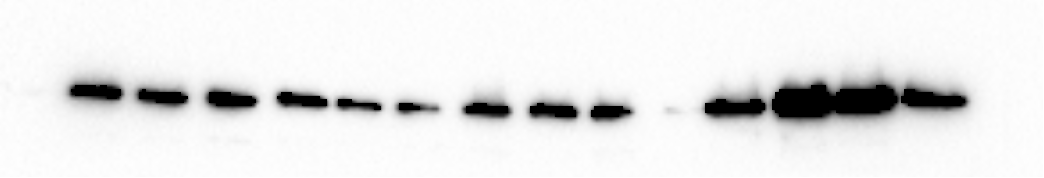

Supplement: Figure 5—figure supplement 1—source data 1. [file elife-92409-fig5-figsupp1-data1.zip › Figure 5-figure supplement 1-source data 1/Uncropped Originals /PanelA - p97 blot .tif]

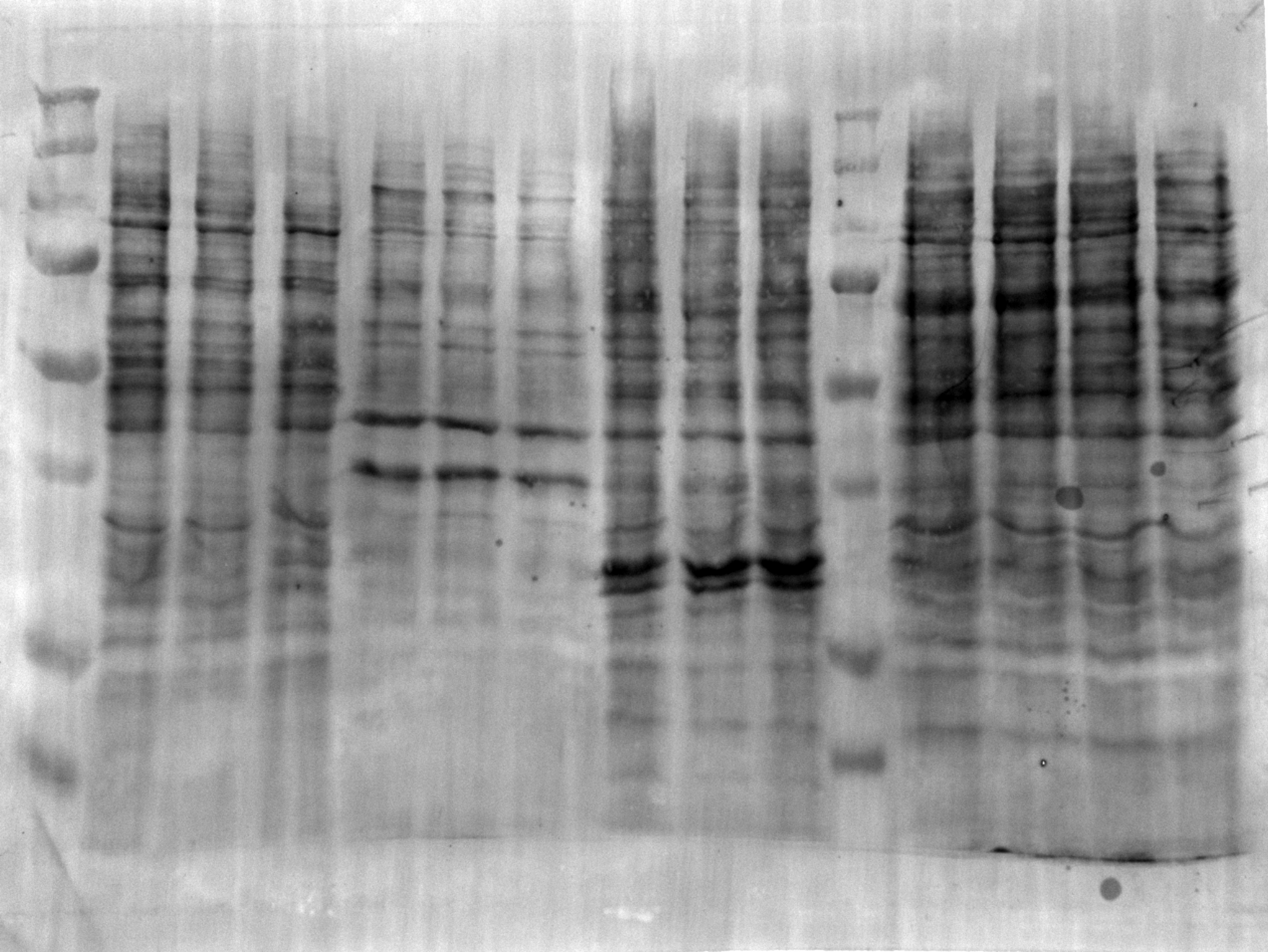

Supplement: Figure 5—figure supplement 1—source data 1. [file elife-92409-fig5-figsupp1-data1.zip › Figure 5-figure supplement 1-source data 1/Uncropped Originals /PanelA - total protein stain.tif]

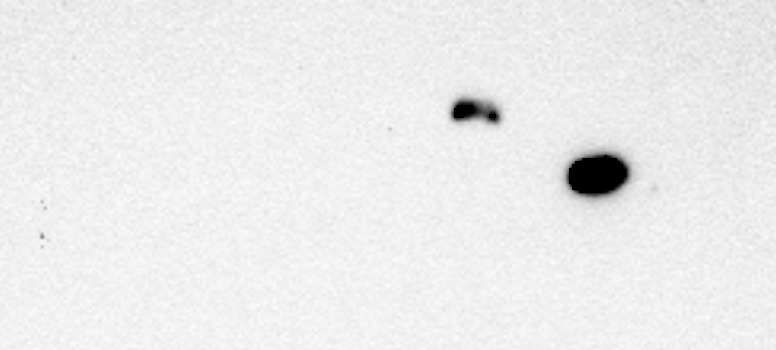

Supplement: Figure 5—figure supplement 1—source data 1. [file elife-92409-fig5-figsupp1-data1.zip › Figure 5-figure supplement 1-source data 1/Uncropped Originals /PanelB - Flag blot.tif]

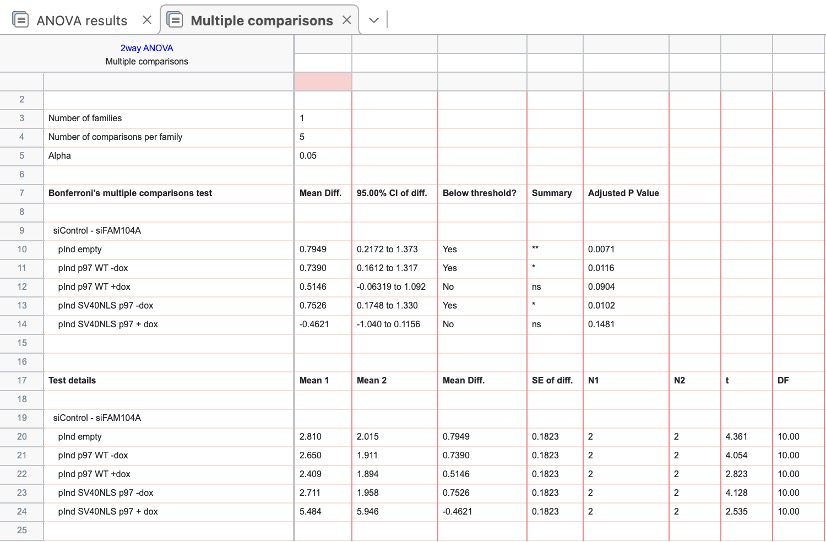

Supplement: Figure 6—source data 1. [file elife-92409-fig6-data1.zip › Figure 6-source data 1/2wayANOVA Figure 6F.jpg]

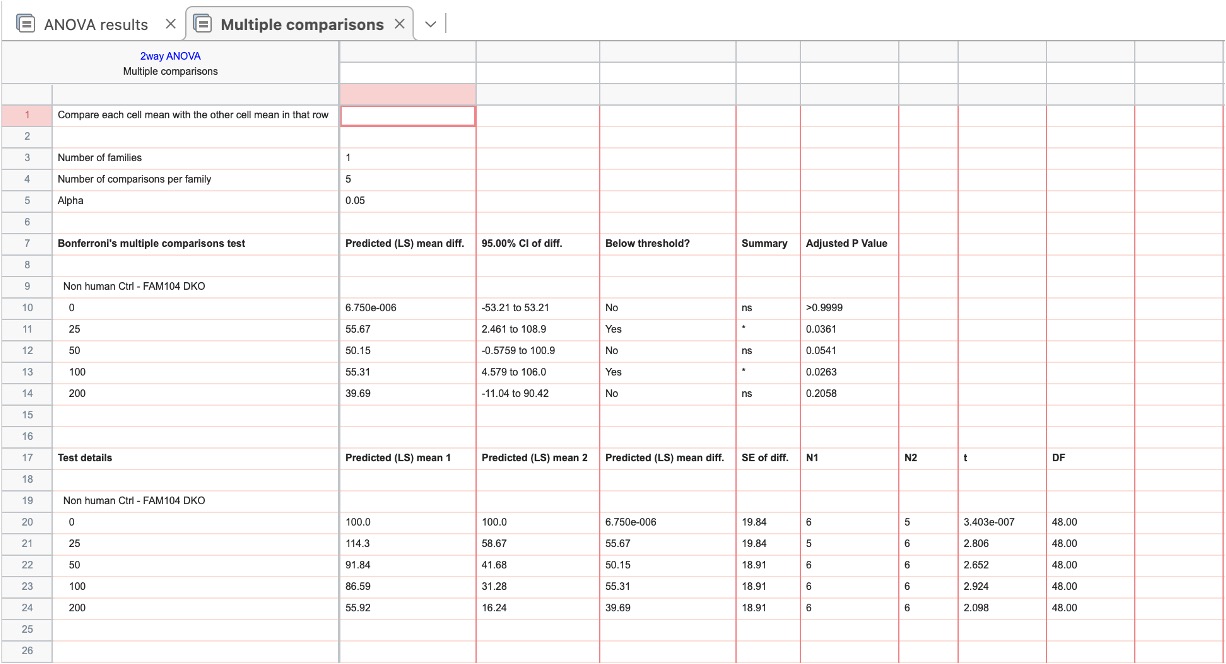

Supplement: Figure 7—source data 1. [file elife-92409-fig7-data1.zip › Figure 7-source data 1/2wayANOVA Figure 7C.jpg]

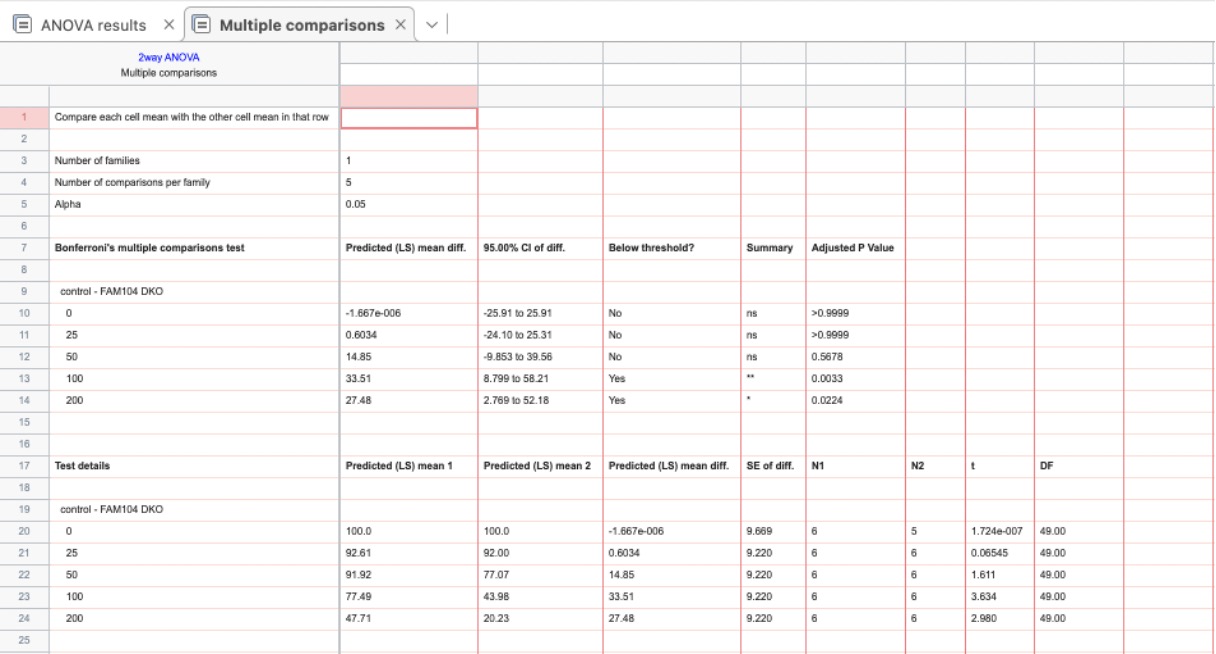

Supplement: Figure 7—source data 1. [file elife-92409-fig7-data1.zip › Figure 7-source data 1/2wayANOVA Figure 7B.jpg]

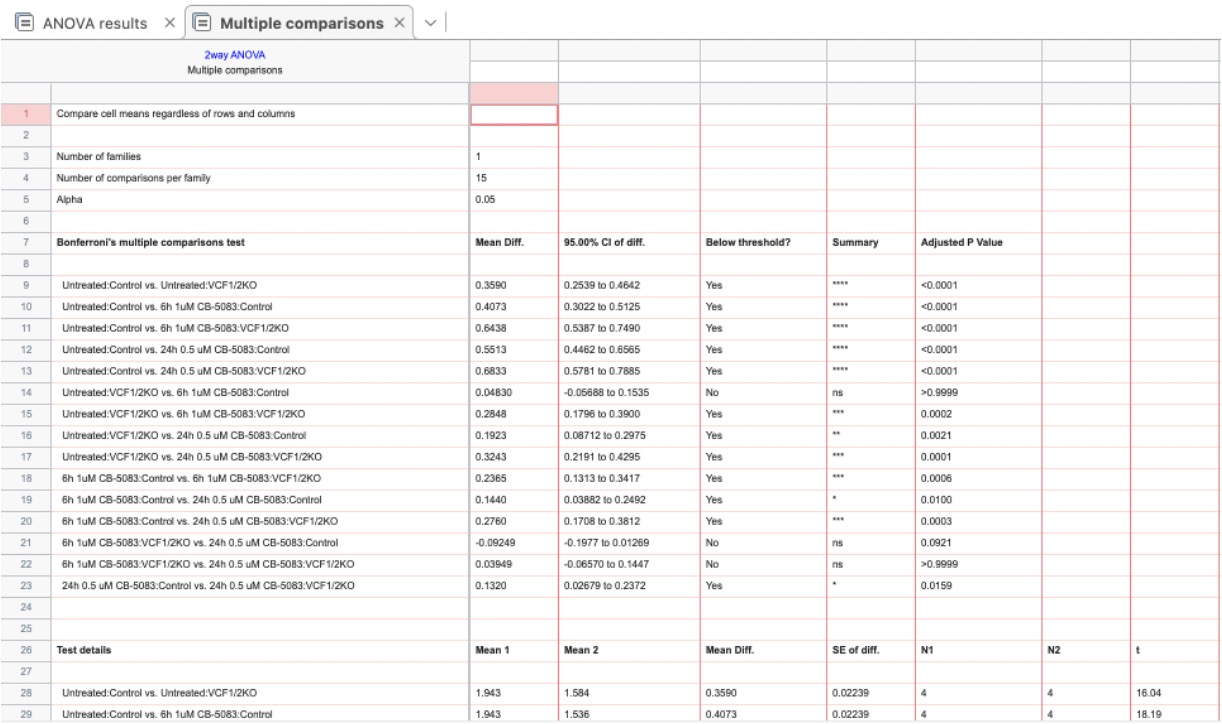

Supplement: Figure 7—source data 1. [file elife-92409-fig7-data1.zip › Figure 7-source data 1/2wayANOVA Figure 7F.jpg]

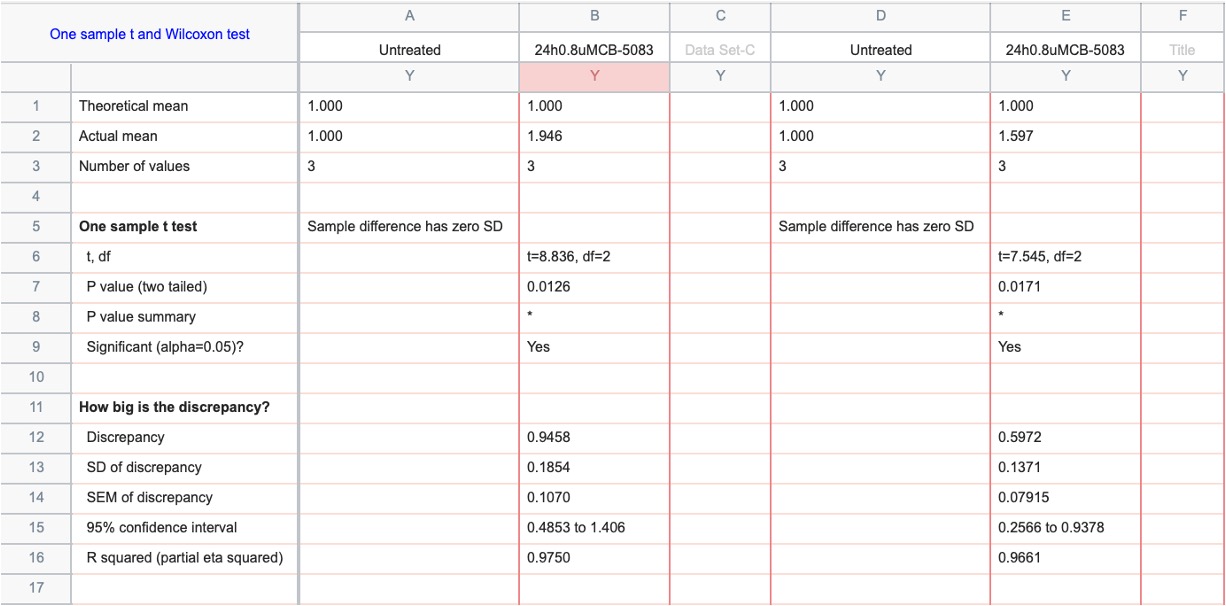

Supplement: Figure 7—source data 1. [file elife-92409-fig7-data1.zip › Figure 7-source data 1/Wilcoxon Figure 7D.jpg]

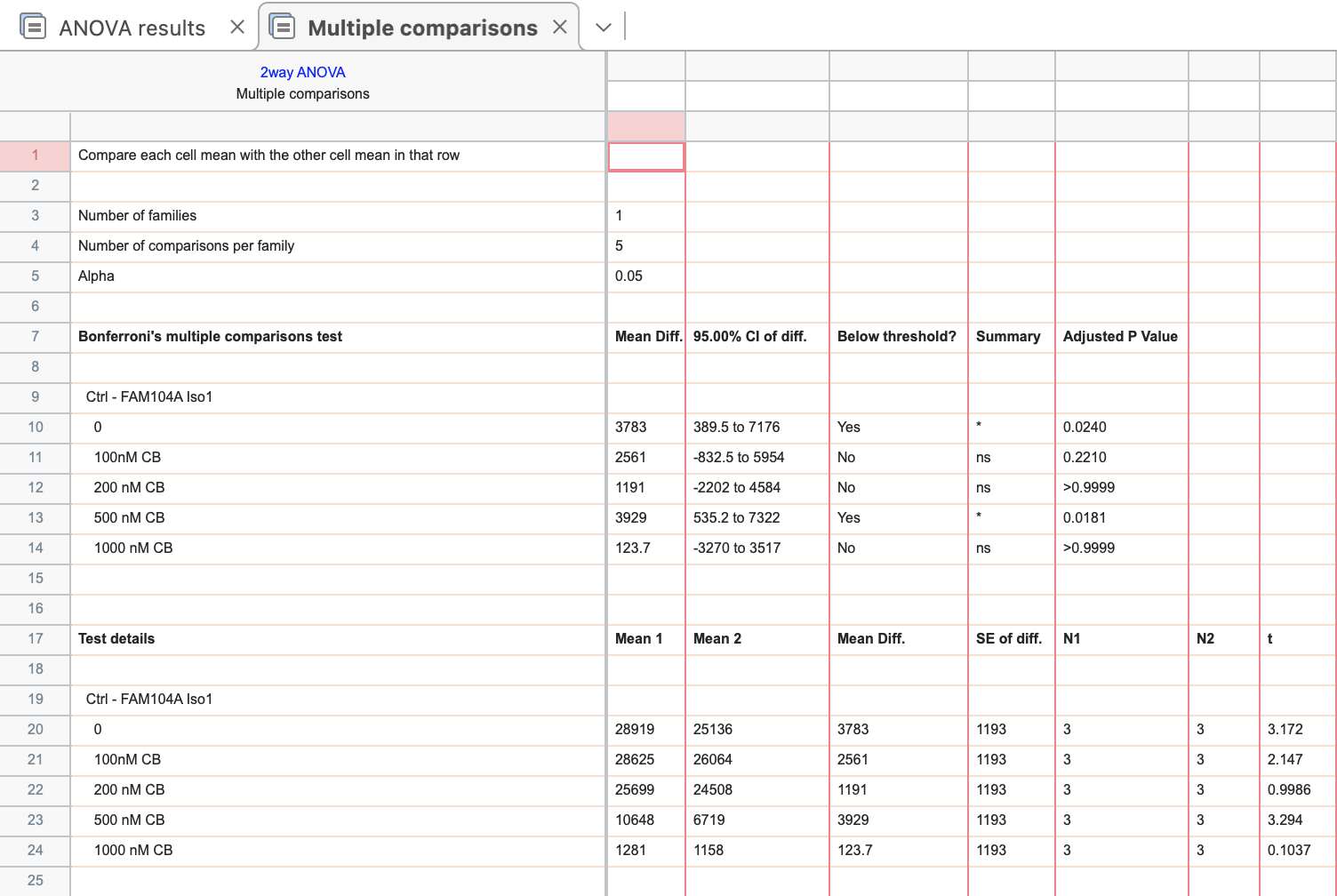

Supplement: Figure 7—figure supplement 1—source data 1. [file elife-92409-fig7-figsupp1-data1.zip › Figure 7-figure supplement 1-source data 1/2way ANOVA Figure 7 Supplement 1.png]
